# Repeat HIV testing practices in the era of HIV self-testing among adults in KwaZulu-Natal, South Africa

**DOI:** 10.1371/journal.pone.0212343

**Published:** 2019-02-22

**Authors:** Charlene Harichund, Pinky Kunene, Sinenhlanhla Simelane, Quarraisha Abdool Karim, Mosa Moshabela

**Affiliations:** 1 Centre for the AIDS Programme of Research in South Africa, Durban, South Africa; 2 Department of Epidemiology, Columbia University, New York, New York, United States of America; 3 School of Nursing and Public Health, University of KwaZulu-Natal, Durban, South Africa; 4 Africa Health Research Institute, KwaZulu-Natal, South Africa; Oregon State University, UNITED STATES

## Abstract

Repeat HIV testing is important in high HIV burden communities to enable sustainability of prevention initiatives; however, an understanding of repeat testing practices is limited. Additional HIV testing approaches may be required to increase testing. HIV self-testing is an additional testing approach, but knowledge on its potential for repeat testing is limited. This study explored repeat HIV testing practices and uptake of HIV self-testing among repeat testers, following exposure to HIV self-testing. HIV testing practices were explored at two time points. During Phase 1, eighty in-depth interviews were conducted among 40 consenting adults, and 30 telephonic contacts were completed during Phase 2. Framework analysis was used to analyse the transcripts from the in-depth interviews. The practice of repeat HIV testing is primarily influenced by HIV status awareness and risk exposure. Thirteen regular testers and one HIV naïve tester at baseline had undergone repeat testing through the use of a traditional testing approach such as HIV counselling and testing as reported in Phase 2. HIV self-testing has a role among repeat testers, but affordability and access are barriers.

## Introduction

Despite the availability of several HIV testing approaches, HIV counselling and testing (HCT) is most commonly utilized and has utility for people who test for the first time [1, 2]. However, the low self-perceived HIV risk, stigma, fear of social exclusion, inconvenient clinic hours and quality of HIV testing services are barriers to uptake of HCT [3], highlighting the need for additional testing options. The World Health Organization (WHO) recommends HIV self-testing (HIVST) as an additional HIV testing approach, to complement existing services associated with HCT [4].

The potential of HIVST to improve the uptake of HIV testing, when compared to traditional HIV testing approaches and its ability to increase the frequency of HIV testing among men who have sex with men (MSM) and male partners of heterosexual women, was recently reviewed [5]. While the uptake of HIV testing is primarily focused on individuals who test for the first time, enabling earlier diagnosis and management of participants, there is little data available on repeat HIV testing [6]. Repeat HIV testing is important, particularly for individuals in high HIV burden communities, and where partner change is frequent but safer sex practices are limited for multiple reasons [1, 7]. Repeat HIV testing is also important as it ensures sustainability and the continued success of prevention programmes [8].

Survey data from sub-Saharan Africa report repeat testing rates from 26% to 87% [8, 9]. Perceptions that a negative HIV result remains unchanged is a major barrier to repeat testing [10]. Offering HIVST as a testing approach together with HCT, may serve to increase repeat HIV testing rates [11]. This study assessed repeat HIV testing practices and the potential role of HIVST among repeat HIV testers, following exposure to HIVST.

## Materials and methods

### Participant selection

Purposive sampling was used to recruit volunteers from either a primary health care clinic offering HIV testing services, which were adjacent to the Centre for the AIDS Programme of Research in South Africa (CAPRISA), or the CAPRISA research clinic. Volunteers recruited from the primary health care clinic were either those who have never had an HIV test (HIV naïve) or who previously had an HIV test (experienced tester). Participants from the CAPRISA clinic who were previously enrolled in a research study with a predetermined frequency of testing based on the study protocol (research tester). Consenting men and women older than 18 years of age, from each of these groups, were eligible for participation in this study.

### Study design

The study was conducted in two phases. Phase 1 included a qualitative assessment of the participants’ HIV testing behaviour at two time points: baseline and after testing with both HIVST and HCT. Participants who tested positive for HIV were referred to appropriate facilities for further care and management. During Phase 2, participants were contacted telephonically, approximately three months after their study visit to determine their uptake of HIV testing and the testing approach used. Ethics approval (reference number: BFC326/15) for the study was obtained from the Biomedical Research Ethics Committee at the University of KwaZulu-Natal. Written informed consent was obtained prior to enrolment into the study.

### Data collection

During study visits, two researchers who were trained in qualitative interviewing, conducted the in-depth interviews (IDIs), using interview guides (S1 Fig). Qualitative discussions focused on HIV testing practices and user experience with both HIV testing approaches (HCT and HIVST), and were recorded and transcribed by the study team. The demographic characteristics of each participant were also collected during their study visit at each clinical research site (S2 Fig).

Telephonic follow-up interviews were undertaken by a research assistant using a structured interview schedule that included the following items: 1) Have you tested for HIV since your study visit? 2) Reason(s) for testing or not testing. 3) Have you considered HIV self-testing? 4) Reason(s) for HIVST or not. Participant responses were recorded in their study files. Four attempts at contacting participants at different time points over four weeks were completed before a participant was considered lost to follow-up.

### Data analysis

The framework analysis method described by Rabiee (2004) was used to analyse the IDI transcripts (S3 Fig) [12]. This method included familiarisation of the data, identification of a thematic framework, indexing, charting, as well as mapping and interpretation [4]. Researcher one developed an initial list of theoretical and emerging codes from a sub-set of four transcripts. Researcher one, who was well versed with literature around HIV self-testing and HIV testing, employed the assistance of researcher two to assist with coding of transcripts. Researcher two was provided with in-depth training about the project and coding. Thereafter, both researchers independently followed the framework analysis method described by Rabiee in 2004 with two transcripts to verify codes and identify new emerging themes. Thereafter analysis, outcomes were discussed and compared to clarify themes and address inter-code discrepancies before proceeding with data analysis of all available transcripts. Theoretical and emergent thematic frameworks were identified by writing memos in the column of the written transcripts in the form of short phrases, ideas or concepts arising from the transcripts. Highlighting and sorting out quotes and making comparisons both within and between cases was conducted through indexing. Data was then charted, which involved removing quotes from their original context and rearranging them under their appropriate thematic content. The final step of the process, mapping and interpretation, involved axial coding and analysis of the data to identify the relationship between the quotes and the links between the data as a whole.

The participants’ telephonic responses were collated, following the review of their study files and univariate analysis completed.

## Results

### Participants

During Phase 1 of the study, 12 male and 28 female participants were enrolled across two clinical research cites (Table 1), resulting in 80 IDIs being completed. The average age of the men and women were 25 and 27 years, respectively. The majority of participants were unemployed, and all participants had access to an HIV testing facility in their community (Table 1).

**Table 1:** Demographic characteristics of participants during Phase 1.

Demographics	Gender	
	Male	Female
**Participants (n)**	12	28
**Cohorts**		
HIV testing naïve	3	7
Research tester *(enrolment in a research study)*	0	12
Experienced tester *(previously tested for HIV)*	9	9
Mean age (years of age) (range)	25 (23–37)	29 (18–48)
**Marital status**		
Single	11	28
Married	1	0
**Employment status**		
Employed	3	8
Unemployed	9	20
**Access to HIV testing facility**		
Yes	12	28
No	0	0

**Fig 1:**
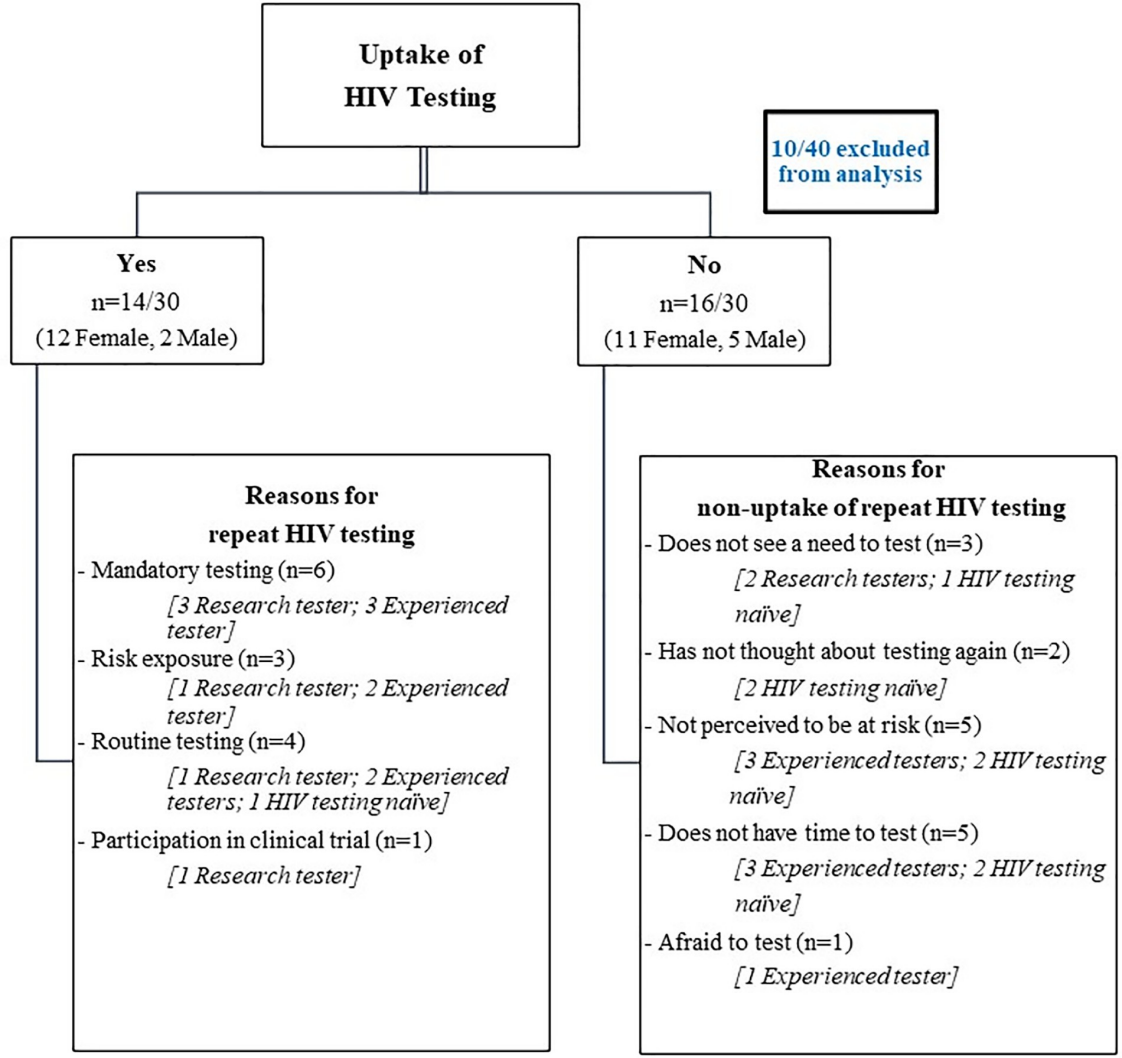
Uptake of HIV testing following exposure to two HIV testing approaches during Phase 2 of study and reasons for HIV testing behaviour.

Thirty (n = 30/40) participants were successfully contacted during Phase 2 of the study, approximately three months after their study visit (Fig 1). Ten participants were excluded from the analysis as eight were unreachable and two tested HIV positive at their study visit (Fig 1). Five male experienced testers, from a total of eight participants, were lost to follow-up. Two men and 12 women, who were experienced testers, underwent repeat testing during Phase 2, which included one female HIV testing naïve participant. Of the 16 participants who did not have a repeat test after their study visit, five were male (two HIV testing naïve and three experienced testers) and eleven females (five HIV testing naïve and six experienced testers).

### Factors that influence repeat HIV testing practice

**Fig 2:**
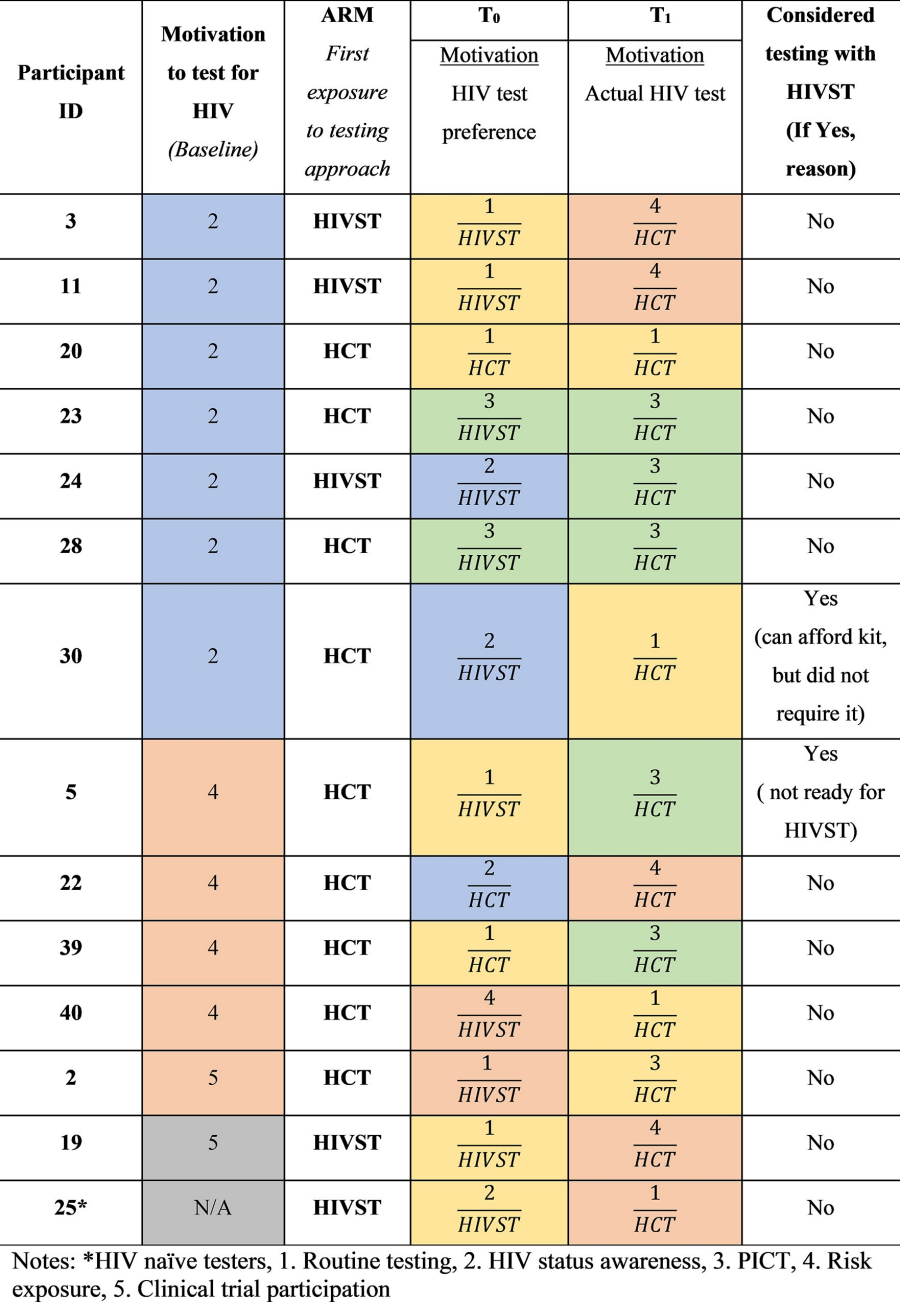
Overview of HIV testing behaviour of participants who tested for HIV post study visit.

In Fig 2, data on motivation to test for HIV at three time points including baseline (prior to their participation in the study), after exposure to HCT and HIVST in the study (T_0_) and during Phase 2, which followed their onsite study visit (T_1_) are presented. At T_0_, participant’s overall preference for either HCT or HIVST and motivation to test was examined. Whilst at T_1_, participants reported on their motivation to test and actual test (HCT or HIVST) used, which provided an important understanding of factors that influence repeat HIV testing behaviour.

**Fig 3:**
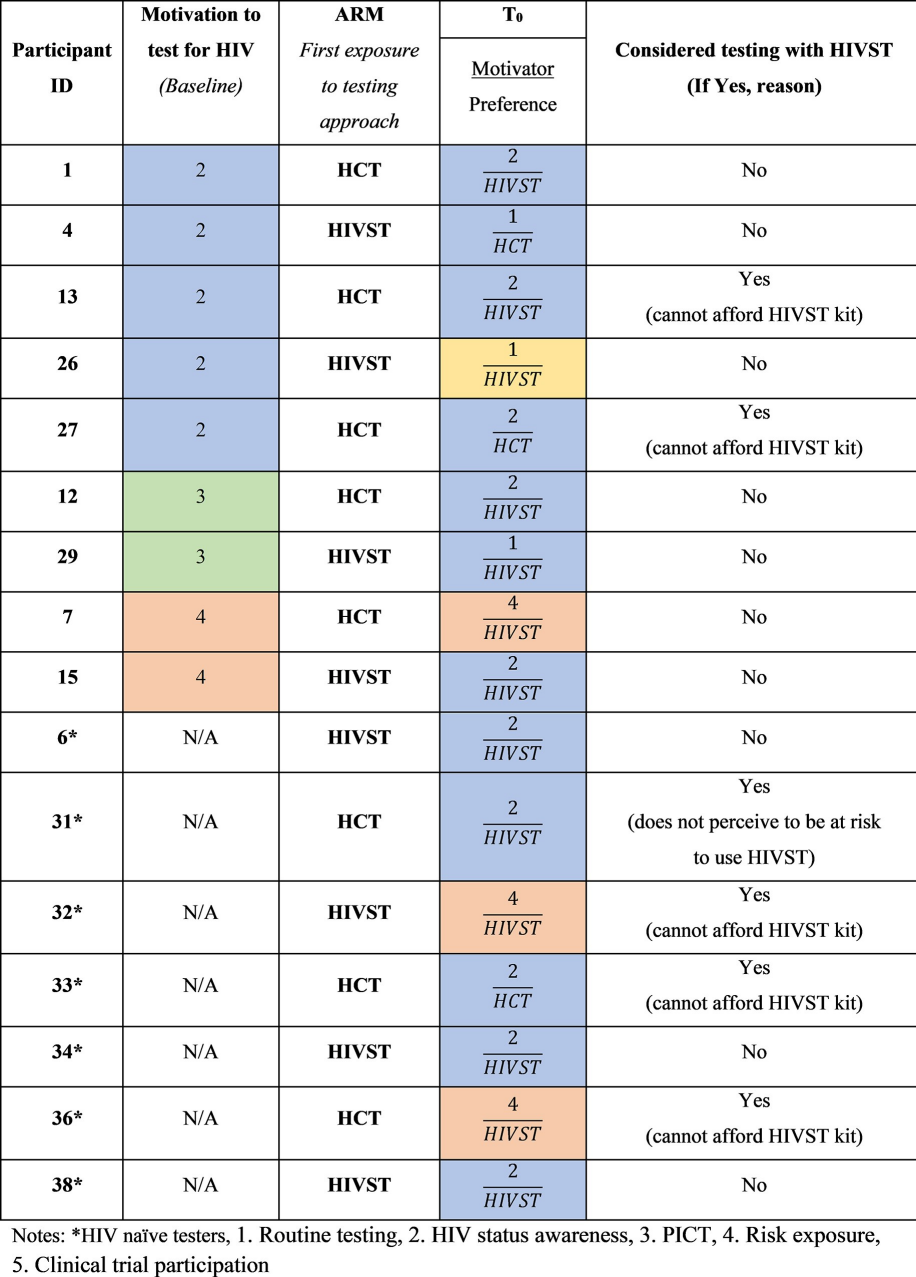
Overview of HIV testing behaviour of participants who did not test for HIV during Phase 2.

Data on HIV testing behaviour of participants who did not test for HIV during Phase 2 and analysed at two time points, baseline and following exposure to HCT and HIVST in the study (T_0_) are presented in Fig 3. HIV testing naïve participants were only assessed at T_0_ as they had no prior testing history. Overall, motivating factors for HIV testing included 1) HIV status awareness, 2) repeat testing as a precautionary measure in response to risk exposure, 3) extending repeat HIV testing to partners, 4) routine testing as part of ‘normal’ repeat HIV testing behaviour and 5) provider-initiated repeat HIV testing.

#### HIV status awareness

Twelve experienced testers and five HIV testing naïve participants reported a desire for HIV status awareness as their reason for an initial HIV test (Figs 2 and 3). However, during the study visits (Phase 1), HIV status awareness was not the primary reason for repeat HIV testing among the same participants (Figs 2 and 3). The desire for knowledge of one’s HIV status could be perceived as wanting to take control of their health by managing their HIV positive or negative status earlier. The pattern observed with HIV testing practice expressed in this study indicates HIV status awareness as the primary reason for routinely connecting an individual with HIV testing services, but secondary factors such as risk exposure may intermittently influence future testing (Fig 2). This behaviour may be driven by their desire to always be aware of their HIV status and was also indicative of their autonomy to test, as one participant mentioned.

*“I always have to know my HIV status*, *which is a good thing for me”* (IDI, Female, RT, 0026).

As participants increased their frequency of repeat HIV testing, they may have been better equipped to discern when they required subsequent HIV testing test as HIV status awareness was no longer a primary reason for testing. This practice was evident through phase 2 data analysis (Fig 2),

#### Repeat testing as a precautionary measure in response to risk exposure

HIV risk exposure influenced initial HIV testing among six experienced testers and two HIV testing naïve participants (Figs 2 and 3). Interestingly, these eight participants were able to understand their risk and seek HIV testing services without prior risk reduction counselling. During the study visits (Phase 1), the majority of participants whose initial HIV test was due to risk exposure, exhibited HIV testing behaviour that ensured they had regular HIV testing (Figs 2 and 3), perhaps as a precautionary measure to ensure that they were aware of their HIV status in light of their risk exposure. Some participants were interested in repeat HIV testing during Phase 1, as they felt they might have been in the window period or as a result of recent exposure to HIV. Despite their regular testing practice, these participants were concerned that their status could change and needed to ensure that they could seek care earlier to manage their result. Protecting oneself by testing for HIV, following risk exposure, is important as it affords an individual the option to manage their result earlier by accessing treatment or prevention services.

*“I would need to test because I don’t know maybe at this moment I could still be in the window period”* (IDI, Female, RT, 0022).

*“I needed to test*, *as I engaged in risky sex and the condom burst”* (IDI, Male, RT, 0008).

#### Routine testing as part of ‘normal’ repeat HIV testing behaviour

Seven participants who tested during the post study visit (Phase 2) reported routine testing as their reason for repeat testing upon entry into this study (Fig 2), which may be indicative of ‘normal’ testing behaviour. There was no direct link between reason for initial testing behaviour and routine testing practice during the study visit (Phase 1) testing (Fig 2). Although participants reported routine testing as their primary reason for repeat testing, the primary rationale for routine testing could be in keeping with testing as a precautionary measure linked to HIV status awareness and risk exposure.

#### Extending repeat HIV testing to partners for regular testers

One female participant believed that her status had always been HIV negative and she should include her partner in the testing process as this would encourage her to test again for HIV. This highlighted the participant’s ability to be cognisant of the relationship between risk exposure and partner testing, where one should always know their partner’s HIV status.

*“I’m so used to testing*, *maybe if I was testing with my boyfriend I would test again”* (IDI, Female, RT, 0028).

#### Provider-initiated HIV testing

**Fig 4:**
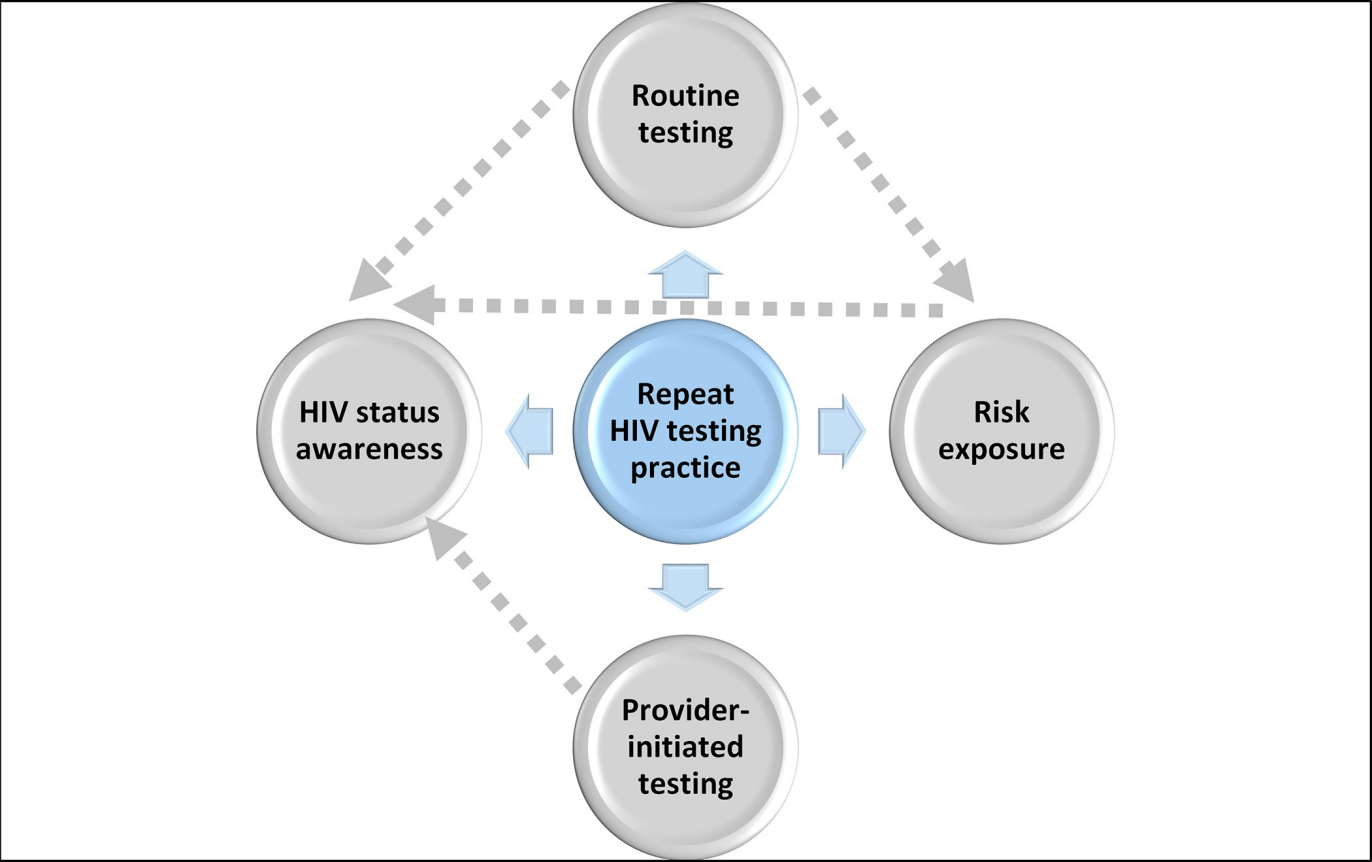
Interrelationship of factors that influence repeat HIV testing practices.

Only two participants reported provider-initiated HIV testing as part of their initial HIV testing practice. One participant underwent HIV testing at her antenatal clinic due to her pregnancy and the second participant tested as a prerequisite to circumcision. Provider-initiated HIV testing was an uncommon testing practice during enrolment into the study (Phase 1) (Figs 2 and 3). However, this testing practice was more frequent during follow-up repeat HIV testing (Phase 2) as six participants displayed this type of testing behaviour (Fig 2). Overall, the participants who accessed health care facilities, post study visit (Phase 2), for pregnancy outcome, contraception-related clinic visits and needle stick injury, reported provider initiated counselling and testing. Door-to-door HIV testing and testing at mobile clinics, and traditional healers who are trained to perform HIV tests and engage with individuals within households, contributed to uptake of HIV testing through community-based testing (Fig 4). Participants viewed community-based testing approaches as convenient opportunities for repeat testing as it was delivered to them and they did not have to leave their homes for testing.

#### Reasons for non-uptake of repeat HIV testing

Experienced testers and HIV testing naïve participants reported no perceived risk and not having time to test, as reasons for not testing during Phase 2 of the study. Risk exposure, as outlined earlier, influenced uptake of repeat testing. Promoting repeat HIV testing behaviour among HIV testing naïve participants would be important to ensure continuity of their HIV status awareness. Perhaps testing during the study was adequate for these participants as time and no desire for repeat HIV testing were highlighted as reasons for non-uptake of HIV testing.

#### Interrelationship of factors that influence repeat HIV testing practices

While four primary factors (Fig 4) influenced HIV testing and repeat testing behaviour among experienced and HIV testing naïve participants, an interrelationship between routine testing, HIV status awareness and risk exposure emerged through which more frequent testing may occur to primarily determine HIV status (Fig 4). Similarly, continuous risk exposure may lead to routine testing for awareness of a person’s HIV status, resulting in repeat testing (Fig 4). Provider-initiated testing (Fig 4) may influence repeat HIV testing but may not be entirely voluntary in high endemic areas where testing is essential to guide clinical management of patients. Therefore, provider-initiated testing may not be linked to voluntary HIV status determination which in turn may limit repeat HIV testing.

### Uptake of HIV self-testing as a repeat testing approach

All participants who tested during Phase 2 used traditional testing approaches such as HCT. It can be noted that one participant who regularly tested, purchased an HIVST kit from her pharmacy but did not use it as she gave it to a family member to use following their recent risk exposure (Fig 2). However, six participants who did not have a repeat HIV test reported a desire to test with HIVST for their next HIV test, of which four were HIV testing naïve participants (Fig 3). Accessibility to HIVST kits was influenced by affordability for five participants who did not have a repeat HIV test, as they were not in a financial position to purchase the kits (Fig 3). Importantly, HIVST could be included as a testing approach within the framework outlined in Fig 4 to prevent missed testing opportunities. For most participants who tested, reports that they were comfortable with their current testing approach and did not require testing with HIVST was noted.

## Discussion

The repeat HIV testing practice was influenced by the interrelationship between the desire for HIV status awareness, routine testing and risk exposure. Although initial HIV testing behaviour may begin with a desire for knowledge of HIV status, routine or regular testing behaviour may be due to risk exposure. This, in turn, leads to HIV status awareness or routine testing to allow constant awareness of HIV status to manage a HIV positive or negative result. HIV status awareness which has been highlighted as the primary reason for repeat testing, is important in reaching the first 90 of the 90-90-90 targets. Contrary to our study, sexual risk taking was proposed as the primary reason for repeat testing among people within a community in Hlabisa in KwaZulu-Natal [8]. Also, people who tested HIV negative were more likely to have a repeat HIV test, which contradicts evidence from other studies that highlighted that people were more likely not to have repeat testing if they tested negative [8, 10]. Therefore, the importance of repeat HIV testing practices should be encouraged during HIV testing campaigns in addition to first-time testing.

The potential of HIVST as a repeat HIV testing approach was highlighted in this study as some participants considered using it despite the availability of HCT; however, missed testing opportunities, mainly among HIV testing naïve participants, were noted due to affordability of HIVST which limited its use. The potential of HIVST can be gleaned from some participants considering use of HIVST for their subsequent HIV test in Phase 2. Several studies advocated for the use of HIVST to increase the frequency of HIV testing [5, 13, 14], and to encourage testing among people who have not tested [9]. Further to this, scale-up of biomedical HIV prevention initiatives such as Pre-Exposure Prophylaxis and microbicides will require regular repeat HIV testing which may cause undue burden on already strained human resources within primary healthcare facilities [15, 16]. Therefore, the potential repeat HIV testing potential of HIV self-testing could be extended to these HIV prevention approaches to reduce the HIV testing burden on primary healthcare facilities. To our knowledge, the use of HIVST as a repeat testing approach with HIV prevention approaches is not yet in existence within sub-Saharan Africa. Thus, future research to evaluate the feasibility of HIVST for repeat HIV testing during implementation of HIV prevention initiatives.

In South Africa, HIVST is primarily available from pharmacies at a cost. Thus, in addition to affordability, the accessibility of HIVST plays a role in the uptake of this HIV testing approach, as well as determining its potential effect on increasing the uptake of HIV testing. This study focused on motivation to test for HIV and preference for HCT or HIVST as a testing approach, but did not explore cost and accessibility of HIVST which are characteristic of feasibility studies. As the price of HIVST kits and access to these testing kits factored in participant’s decision to undergo repeat HIV testing, future research should be directed toward feasibility studies evaluating distribution models that will ensure cost-effective and easily accessible HIVST kits which, in turn, may promote the uptake of HIVST.

A study in Uganda found that high-risk populations who have high rates of HIV and sexual risk behaviour, displayed an increased preference for HIVST for repeat testing [17]. Since risk exposure was identified as a factor that influences the repeat testing practice, HIVST may be an important addition to the HIV testing framework to increase uptake of repeat HIV testing. This study has several limitations. Firstly, the small sample size together with the use of purposive sampling with its selectiveness bias limits the generalizability of these findings as, the cohort is not representative of the primary target of HIVST. The cohort selected does represent individuals who may have a desire to test for HIV but may face barriers to repeat testing associated with available testing approaches such as HCT. Secondly, the study may have benefited from participants presenting at clinical research sites for follow-up visits to determine their uptake of HIVST which would have enabled us to further explore HIV testing behaviour. Thirdly, the outcome of repeat HIV testing may have been limited by the strict adherence to the three-months follow-up period as participants may have been found to have undergone repeat testing if the follow-up period was extended.

## Conclusion

Repeat testing practice is influenced primarily by desire for HIV status awareness and risk exposure. The potential for HIVST as an additional repeat testing approach exists but is limited due to affordability and accessibility as HIVST is not available within public healthcare facilities. The cost of HIVST requires further consideration to expand its accessibility to individuals whose desire for repeat HIV testing with HIVST is restricted by affordability. This study provided supporting evidence around repeat HIV testing practices that includes HIVST as an additional testing approach within the HIV testing framework.

## Supporting information

S1 Fig Qualitative interview guide. (PDF)

S2 Fig Demographic and locator information tool. (PDF)

S3 Fig Transcripts from interviews. (DOCX)
