# Supplementary material for: Repeat HIV testing practices in the era of HIV self-testing among adults in KwaZulu-Natal, South Africa
Source: PLoS One. 2019 Feb 22;14(2):e0212343. doi: 10.1371/journal.pone.0212343 (PMC6386490; doi:10.1371/journal.pone.0212343)
Supplement: S1 Fig — (PDF) [file pone.0212343.s001.pdf]

PHASE 2

IN-DEPTH INTERVIEW GUIDE

Project Title: Acceptability and Feasibility of HIV self-testing in KwaZulu-Natal, South Africa.<br><br>Principal Investigator: Ms Charlene Harichund<br><br>Co-Investigators: Dr Mosa Moshabela and Professor Quarraisha Abdool Karim

WHAT IS THE PURPOSE OF THE INTERVIEW?

To determine participants perceptions about HIV, HIV testing and HIVST.

WHAT INFORMATION AM I INTERESTED IN EXPLORING?

- ✓ Perceptions on why people test for HIV or why they don't
- ✓ Reasons for frequent testing versus infrequent testing
- ✓ Concerns participants may have about HIVST
- ✓ Perceptions on acceptability of HIVST
- ✓ Usability of HIVST
- ✓ Need for counselling when testing for HIV
- ✓ Exploring participant's perception of linkage to care, how would they manage a positive/negative result, what would they do? (Access care, behaviour change, access prevention options for HIV
- ✓ Would participants prefer a choice of testing methods? Sometimes they may have a HIV test at a testing facility and sometimes have HIVST
- ✓ What would they perceive as benefits of HIVST
- ✓ What are possible perceived challenges associated with HIVST
- ✓ Disposal of testing kits
- ✓ Literacy – would people be able to read and understand instructions in HIVST kit
- ✓ Is the contact information provided with HIVST kit adequate for participants to manage their results?

WHO NEEDS THIS INFORMATION, AND WHAT ARE THEY GOING TO DO WITH IT?

Policy makers who will regulate HIVST, Community members

PROCEDURE:

Today you will undergo several interviews and undergo HIV testing using 2 different methods (HIV Counselling and Testing and HIV Self-Testing). During the interviews your opinion and experience related to the 2 HIV testing methods will be requested.

Overview of Phase 2

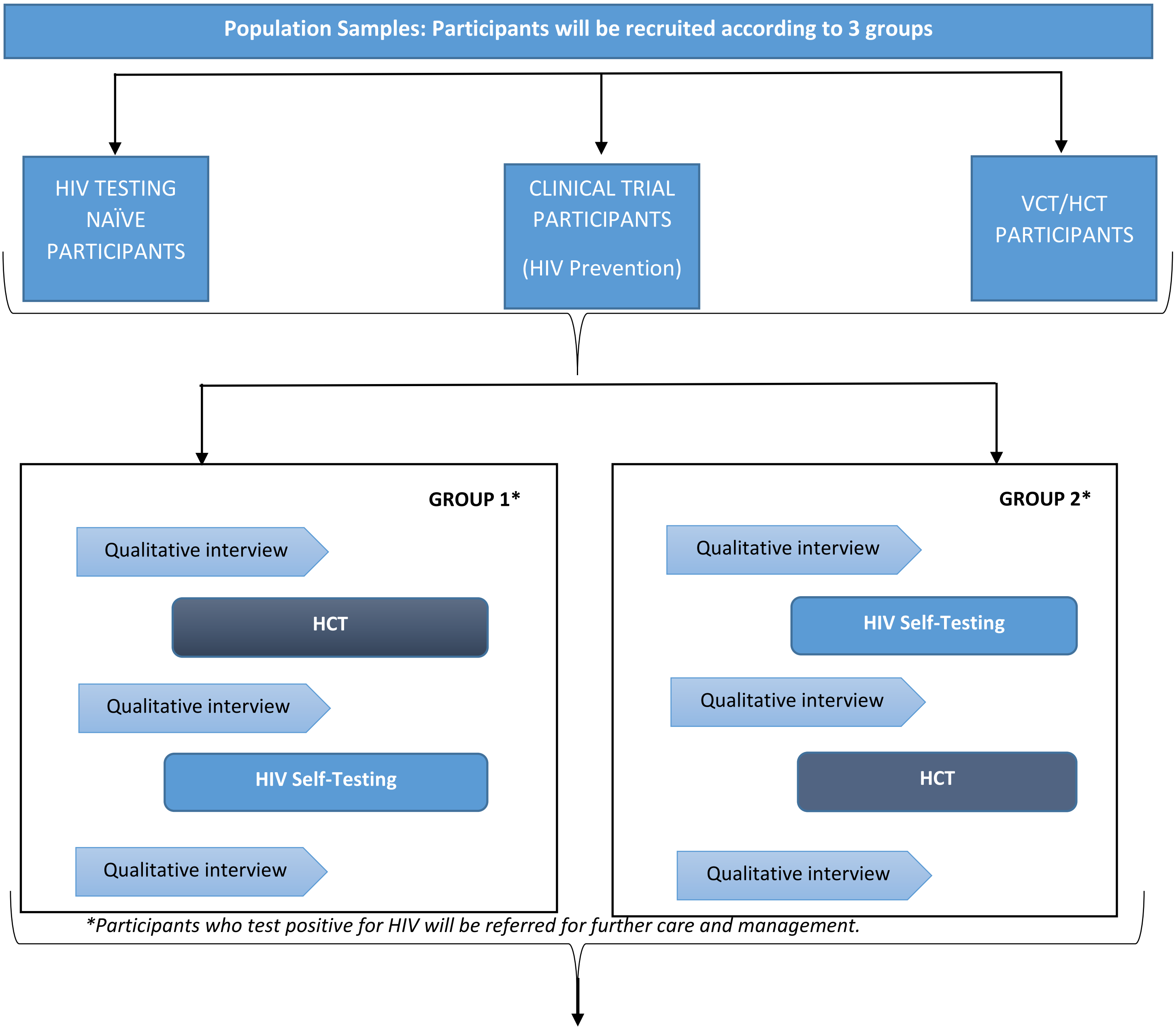

**Participants who test positive for HIV will be referred for further care and management.*

IMB MODEL FOR HIVST

INFORMATION	MOTIVATION	BEHAVIOUR
Provide information about HIV, HIV testing and HIVST	What would be motivators for testing	Monitor behaviour change through uptake of HIV Testing and willingness to use HIVST
What is HIV?<br>Importance of HIV testing<br>Challenges associated with poor uptake of HIV testing<br>What is HIVST<br>Benefits of HIVST	Personal motivators for testing?<br><br>Social motivators for testing?	Contact participant after 3 months to assess uptake of HIV testing<br><br>Would participant prefer HIVST or regular VCT

OVERALL OUTCOME: Did HIVST increase uptake of HIV testing

INTERVIEW 1: HCT/HIVST

1. Have you tested for HIV?
    a. If yes, what made you test?
    b. If no, what prevented you from testing?

2. What made you decide to test today?
    a. Know your status?
    b. Routine 3 monthly testing?

3. How big of an issue do you think HIV/AIDS is for people in your community and what makes it an issue of greater or lesser importance?
    a. PROBE: Has the importance of HIV/AIDS changed in the community over the past few years and if so why?
    b. PROBE: Is it more of an issue for some groups than for others, and if so, which groups and why?
    c. PROBE: Is there denial or do they accept that AIDS is an issue?

4. What is the reputation of the HIV testing locations in your community?
    a. PROBE: Is testing fully confidential? Explain.
    b. PROBE: What are providers like?

5. What do you think are the greatest barriers to HIV testing in your community?
    a. PROBE: Are people's attitudes and beliefs about HIV barriers?
    b. PROBE: What structural factors (e.g. availability of services, lack of resources, lack of privacy in the community, etc.)?

6. What do you know about HIVST?

7. Where did you hear about HIVST?

8. What do you perceive as possible challenges to HIVST?

9. What do you think would be the benefits of HIVST

INTERVIEW 2: HCT/HIVST

1. What did you think about HIV testing using this method? Describe your experience.

2. What would motivate you to test again for HIV using this method?

3. What would discourage you from testing for HIV using this method?

4. What were challenges you experienced or perceived challenges using this HIV testing method?

INTERVIEW 3: HCT/HIVST

1. After testing through HCT and HIVST, which method do you prefer?
    a. What makes you prefer this method?
    b. What discouraged you from the method you did not choose?

2. Which HIV testing method would increase uptake of HIV testing?

Now we will explore your experience with HIVST:

3. <u>Usability and Distribution of HIVST kits:</u>
    a. If you were to obtain a HIVST kit, where would you store it for use?
    b. How/where would you dispose of used HIVST?
    c. Where would you conduct your HIVST?
    d. How much education and training do you think you would need before you can do the HIVST on your own? What type of education and training would you require?
    e. Do you think community members will require training before scale-up of HIVST?
    f. What information should be included in instructions?
    g. What do you perceive as common errors that could be made when using the HIVST kit?
    h. What would be the ideal distribution point for HIVST kits?
    i. Would you access HIVST kits if you had to pay for them?
        i. If yes/no, why?

4. <u>Acceptability of HIVST:</u>
    a. Do you think people in your community would use HIVST kits?
        i. What would motivate/discourage them from using the HIVST kit?
        ii. What would motivate/discourage you from using HIVST kit?
    b. What population do you think would benefit from using HIVST kits for testing for HIV?
        i. Men
        ii. Women
        iii. Teenagers
        iv. Elderly
    c. If you believe HIVST is not acceptable, why do you think it is not acceptable and what can be done to make it acceptable?
    d. What are possible concerns/barriers associated with HIVST?
    e. How do you think people in your home and community would respond to finding out that you are using an HIVST kit?
    f. Would you encourage/ motivate people to test using HIVST kit within your family, your friends, and community members?

5. <u>Need for HIV counselling:</u>
    a. How important do you think HIV counselling is for HIV testing?
        i. What makes it important/not important?
    b. Would written counselling as part of instructions be adequate?
        i. If yes/no, why?

6. Social Harms associated with HIVST:
    a. What do you think you would do if you tested positive for HIV using HIVST kit?
    b. What do you think you would do if you tested negative for HIV using HIVST kit?
    c. What do you think your community reaction would be if they found out you testing using HIVST kit?
    d. Do you think there would be any social harms associated with HIVST?
        i. If yes, what type of social harm would be encountered?
        ii. If no, why not?

7. General:
    a. Do you thing HIVST would have any impact of health seeking behaviour of participants?
        i. Would people access prevention or treatment options sooner if they tested for HIV sooner?
    b. Do you think people would change their risky sexual behaviour if they tested for HIV using HIVST?
    c. Do you think there would be any stigma or discrimination associated with HIVST?
    d. What are possible barriers that would be associated with HIVST?
    e. What do you think would be possible advantages of using HIVST to test for HIV?
