# Supplementary material for: Repeat HIV testing practices in the era of HIV self-testing among adults in KwaZulu-Natal, South Africa
Source: PLoS One. 2019 Feb 22;14(2):e0212343. doi: 10.1371/journal.pone.0212343 (PMC6386490; doi:10.1371/journal.pone.0212343)
Supplement: S2 Fig — (PDF) [file pone.0212343.s002.pdf]

PHASE 2:

IN-DEPTH INTERVIEW DEMOGRAPHICS

Participant Identification (PID): ____

I will start by asking you some general questions about yourself:

Age or Date of Birth: ____

Gender:

☐ Male ☐ Female

Marital Status:

☐ Single ☐ Married ☐ Other (*Specify*):______________________________

Highest level of education:

☐ Primary School not complete ☐ Primary School complete

☐ Secondary School not complete ☐ Secondary School complete

☐ Attended University/College ☐ No Schooling

Employment status:

☐ Employed ☐ Unemployed

Name of your local clinic: ____

PHASE 2:

IN-DEPTH INTERVIEW LOCATOR INFORMATION

Please provide us with your contact details so that we can contact your for follow-up visits if required.

Name: ____

Address: ____

____

____

Contact Number 1 (participant): ____

Contact number 2 (name and contact number): ____________________________

____

Comments: ____

____

____

____
