# Supplementary material for: Repeat HIV testing practices in the era of HIV self-testing among adults in KwaZulu-Natal, South Africa
Source: PLoS One. 2019 Feb 22;14(2):e0212343. doi: 10.1371/journal.pone.0212343 (PMC6386490; doi:10.1371/journal.pone.0212343)
Supplement: S3 Fig — (DOCX) [file pone.0212343.s003.docx]

**INTERVIEW TRANSCRIPTION**

**Participant: 0026**

**INTERVIEW 1: HCT/HIVST**

**SQT**. Have you tested for HIV?

**NN**. yes

**SQT**. If yes, what made you test?

**NN.** I wanted to know my status.

**SQT.** Routine 3 monthly testing?

**NN**. I test every 3 months, but I last tested in June last year.

**SQT.** How big of an issue do you think HIV/AIDS is for people in your community and what makes it an issue of greater or lesser importance?

**NN**. The people I live with they see it as important but they are scared to test that what I hear from them. They say it’s better not to know because if they do know they will get ill and get stressed, I told a friend of mine about the importance of testing and she ended up testing and came back to tell me that the test went well, she saw the importance in the end.

**SQT**. Has the importance of HIV/AIDS changed in the community over the past few years and if so why?

**NN**. The way I see it people do not care to test, they always say that they are scared. Only when their bodies start to change, then they go for the test.

**SQT**. Is it more of an issue for some groups than for others, and if so, which groups and why?

**NN**. Teenagers, they are sexually active but do not protect themselves and also fall pregnant at a young age.

**SQT.** Is there denial or do they accept that AIDS is an issue?

**NN**. They accept it as an issue.

**SQT.** What is the reputation of the HIV testing locations in your community?

**NN**. What I can say, the location has a good reputation

**SQT.** Is testing fully confidential?

**NN**. Yeah I would say so.

**SQT.** What are providers like?

**NN.** They are good they make you comfortable and not to lose hope about the future.

**SQT.** What do you think are the greatest barriers to HIV testing in your community?

**NN**. Ok what I’ve heard, they will say I know how I carry myself and I also know how the person I’m with is carrying themselves and have that as an excuse not to test.

**SQT.** What structural factors (e.g. availability of services, lack of resources, lack of privacy in the community, etc.)?

**NN**. It’s a safe place that’s all right, but at times you go for testing there and you find in the room there’s a counsellor and another member of stuff in the room, and you do the test there while the other one is sitting listening in on the conversation and that made me so uncomfortable I actually wanted to stop because I knew that my results were not private.

**SQT.** What do you know about HIVST?

**NN.** I know nothing. – HIV status awareness

**SQT.** Where did you hear about HIVST?

**NN**. I heard for the first time from you guys.

**SQT.** What do you perceive as possible challenges to HIVST?

**NN**. Maybe its fear, and for me I didn’t think this would be a good thing for people ,but as time goes on people will find help in these kits. – Fear of testing with HIVST kits

**SQT**. What do you think would be the benefits of HIVST

**NN**. What could I say…uhhhhm it’s that people will know how their status is, and some people fear being tested by another person .I also think there will be harm ,if a person finds that they are positive it could be hard on them, who would they tell first? Maybe if they had been tested at a clinic they would have been told what to do next and given comfort.

**INTERVIEW 2: HIVST**

**SQT.** What did you think about HIV testing using this method? Describe your experience.

**NN.** I think HIVST is a good thing because as people we are scared to go test at the clinics, but as for other people they could be nervous about the kit and just go on believing that if their partner is still negative then they too must be negative.

**SQT.** What would motivate you to test again for HIV using this method?

**NN.** it would be that I always have to know my health status. Which is a good thing for me.

**SQT.** What would discourage you from testing for HIV using this method?

**NN.** I don’t see what could discourage me.

**SQT.** What were challenges you experienced or perceived challenges using this HIV testing method?

**NN**. I had a hard time with the lancet I do not know how to use it to prick myself, after that the rest of the process went well.

**INTERVIEW 3: HCT/HIVST**

**SQT.** After testing through HCT and HIVST, which method do you prefer?

**NN.** I liked HIVST.

**SQT.** What makes you prefer this method?

**NN.** Because I’m alone its safe and private.

**SQT.** What discouraged you from the method you did not choose.

**NN.** I can use the other testing method just to confirm.

**SQT.** Which HIV testing method would increase uptake of HIV testing?

**NN.** It’s HIVST

**Usability and Distribution of HIVST kits**

**SQT.** If you were to obtain a HIVST kit, where would you store it for use?

**NN.** I would put in my wardrobe where nobody can reach it.

**SQT.** How/where would you dispose of used HIVST?

**NN.** I would wrap it in tissue and throw it in the neighbours bin.

**SQT.** Where would you conduct your HIVST?

**NN.** If everyone at home is gone and I’m alone I would do it in, my room**.**

**SQT.** How much education and training do you think you would need before you can do the HIVST on your own?

**NN.** Ok maybe, I would need to be mentally prepared that I need to be strong, relax and tell myself that I’m going through with the test**.**

**SQT.** Do you think community members will require training before scale-up of HIVST?

**NN.** Yes, It’s being educated on the kit on what it does and how it does it, some people will not just automatically know how to use the kits we are not all the same, like I said there needs to be a paper with all the steps on it.

**SQT**. What information should be included in instructions?

**NN.** Hmmm the way I see it everything is fine with the instructions.

**SQT.** What do you perceive as common errors that could be made when using the HIVST kit?

**NN.** Maybe they could forget to use the alcohol swab to wipe off the germs on the finger**.**

**SQT.** What would be the ideal distribution point for HIVST kits?

**NN.** At stores like Shoprite, the chemist yes those shops.

**SQT**. Would you access HIVST kits if you had to pay for them?

**NN**. Yes

**SQT**. Why?

**NN.** I want to know what my status is.

**4. Acceptability of HIVST:**

**SQT.** Do you think people in your community would use HIVST kits?

**NN.** A lot

**SQT.** What would motivate/discourage them from using the HIVST kit? ii. What would motivate/discourage you from using HIVST kit?

**NN.** The fact that you test alone. - Privacy

**SQT.** What population do you think would benefit from using HIVST kits for testing for HIV?

**NN.** Males and females but especially females because men don’t like to test.

**SQT.** If you believe HIVST is not acceptable, why do you think it is not acceptable and what can be done to make it acceptable??

**NN.** I think the fear that it’s you who tests and there’s no one to advice you. What can be done is to run adverts that will change the mind set to believing that this is a good kit.

**SQT.** What are possible concerns/barriers associated with HIVST

**NN.** I do not know what could possibly be barriers.

**SQT.** How do you think people in your home and community would respond to finding out that you are using an HIVST kit?

**NN.** They would ask what the matter why do you always test . Do you not trust yourself?

**SQT.**  Would you encourage/ motivate people to test using HIVST kit within your family, your friends, and community members?

**NN.** A lot sister.

**5. Need for HIV counselling:**

**SQT.** How important do you think HIV counselling is for HIV testing?

**NN.** It’s so important because as people we take varied decisions, maybe if I receive counselling whatever wrong thing that I may have been thinking counselling may then change my mind.

**SQT.** Would written counselling as part of instructions be adequate?

**NN**. Yes

**SQT**. Yes why?

**NN.** Because it would have all the information on what to do, when and how.

**6. Social Harms associated with HIVST**:

**SQT.** What do you think you would do if you tested positive for HIV using HIVST kit?

**NN.** I would be stressed the whole day, angry and stressed**.**

**SQT.** What do you think you would do if you tested negative for HIV using HIVST kit?

**NN**. I would have been happy, no stress and proud.

**SQT.** What do you think your community reaction would be if they found out you testing using HIVST kit?

**NN.** They would judge me and ask if I’m 100% sure that that this kit really works. Most people do not believe in the over the counter methods they trust things that are done at the hospital, they will question if it’s right does it work.

**SQT.** Do you think there would be any social harms associated with HIVST? i. If yes, what type of social harm would be encountered?

**NN**. Ja but I think so in criticising the product, and just general questions why is this thing in shops, I tell you there will be negative thoughts on this kit.

**7. General**:

**SQT**. Do you thing HIVST would have any impact of health seeking behaviour of participants?

**NN.** Yes, I think so.

**SQT.** Would people access prevention or treatment options sooner if they tested for HIV sooner?

**NN.** Yes sister

**SQT**. Do you think people would change their risky sexual behaviour if they tested for HIV using HIVST?

**NN.** It would be due the availability of the kit, you know that u can access an HIV test at any given time after sexual activity.

**SQT.** Do you think there would be any stigma or discrimination associated with HIVST?

**NN**. I’m not sure about that one.

**SQT.** What are possible barriers that would be associated with HIVST?

**NN.** I would think issues would be there for people that lack privacy in their homes and know that they cannot find a quiet space to test they would discriminate it maybe**. –** Private testing space

**SQT**. What do you think would be possible advantages of using HIVST to test for HIV?

**NN.** Advantages would be privacy and convenience, but for this kit to help people it needs to be relatively cheap.

**Participant: 0022**

**INTERVIEW 1: HCT/HIVST**

**SQT**. Have you tested for HIV?

**KN**. Yes I have

**SQT**. If yes, what made you test?

**KN**. I was sick, and when I would urinate it would be painful, and I knew that with my boyfriend at the time I wasn’t his only girlfriend.

**SQT**. What made you decide to test today?

**KN**. Because I want to know where I stand

**SQT**. Routine 3 monthly testing?

**KN**. Truth be told I have never left home randomly and said I will go test at the clinic, I test because I have to and when I joined the gel study they told me I had to test.

**SQT**. How big of an issue do you think HIV/AIDS is for people in your community and what makes it an issue of greater or lesser importance?

**KN**. As I see it, HIV is not the issue but seeing the sick people especially the males because if you tell them about testing they don’t want to hear about it .We ladies if we feel sick we go test first.

**SQT**. Has the importance of HIV/AIDS changed in the community over the past few years and if so why?

**KN**. I see that it’s changed now before people did not understand HIV, you would be isolated in the community.

**SQT**. Is it more of an issue for some groups than for others, and if so, which groups and why?

**KN**. I know people who come up to you and tell they have HIV, so you can live with it and it’s manageable with pills. It’s the males that still get sick, they even believe they are bewitched.

**SQT**. Is there denial or do they accept that AIDS is an issue

**KN**. They accept it now before there was denial.

**SQT**. What is the reputation of the HIV testing locations in your community?

**KN**. Ok I will start with the clinic nearest to me, they don’t like going there because there is a lot of people who know you there, and they know if you there for HIV related matters because you get sent to a white house and everyone knows why people go there .So people then prefer to go to clinics that are far from their home.

**SQT**. Is testing fully confidential?

**KN**. Yes, I think so, I’ve never heard that a nurse has spread rumours.

**SQT.** What are providers like?

**KN**. I think they are ok they keep the secrets of the patients, and the clinics you have to be separated to different areas.

**SQT**. What do you think are the greatest barriers to HIV testing in your community?

**KN**. Its fearing what people will say, if they see you going to test then what will they think, before they use to complain that Thiers too many pill

**SQT**. What structural factors (e.g. availability of services, lack of resources, lack of privacy in the community, etc.)?

**KN**. No I don’t see that.

**SQT**. What do you know about HIVST?

**KN**. I did not know anything

**SQT**. Where did you hear about HIVST?

**KN**. Firstly the way I see it, the thing to prick yourself with is painful, they could buy the kit and not use it.

**SQT**. What do you perceive as possible challenges to HIVST?

**KN**. It’s that only you will know your status nobody else will know, and some people don’t trust the nurses so.

**SQT**. What do you think would be the benefits of HIVST?

**KN**. Its private and I can even test my child at home.

**INTERVIEW 2: HCT/HIVST**

**SQT**. What did you think about HIV testing using this method? Describe your experience.

**KN**. For me HCT is fine because I’m scared of needles, because you need someone to talk to you.

**SQT.** What would motivate you to test again for HIV using this method?

**KN**. It’s because I don’t know maybe at this moment I could still be within the window period.

**SQT.** What would discourage you from testing for HIV using this method?

**KN**. I think if I were being tested by someone I know they could go and tell other people.

**SQT.** What were challenges you experienced or perceived challenges using this HIV testing method?

**KN**. Its fear that they will know your results.

**INTERVIEW 3: HCT/HIVST**

**SQT**. After testing through HCT and HIVST, which method do you prefer?

**KN**. It’s being tested by you, HCT

**SQT.** What makes you prefer this method?

**KN**. It’s because I know that there will be advice that you will give me before and after my results, and I’m scared to poke myself.

**SQT**. Which HIV testing method would increase uptake of HIV testing?

**KN**. I see HIVST, because people will know that their results are a secret, that’s the better way.

**SQT.** If you were to obtain a HIVST kit, where would you store it for use?

**KN**. Ja, it must not be just anywhere it has to be a hidden place.

**SQT.** How/where would you dispose of used HIVST?

**KN**. Because there’s sharps and needles I would not throw it in an area where there are kids

**SQT.** Where would you conduct your HIVST?

**KN**. I would sit on my bed and close, lock the door, ja in my room to make sure that I am alone and ensure that I dispose properly of the things that I used.

**SQT**. How much education and training do you think you would need before you can do the HIVST on your own?

**KN**. It’s a lot like being told how to do the test, because if you’ve never done an HIV test you can’t think that you can, you could put the blood sample in the wrong hole.

**SQT.** Do you think community members will require training before scale-up of HIVST?

**KN**. I think they do, you can’t just do this thing without being taught.

**SQT.** What information should be included in instructions?

**KN**. I think its fine because they even included the time, when I look at it if you follow the instructions you can do it.

**SQT**. What do you perceive as common errors that could be made when using the HIVST kit?

**KN**. Firstly I see that they can put the blood sample in the wrong place.

**SQT.** What would be the ideal distribution point for HIVST kits?

**KN**. It’s the clinic because everyone has a clinic near to them that they use.

**SQT.** would you access HIVST kits if you had to pay for them?

**KN**. Yes, I would be able to buy it

**SQT**. If yes/no, why?

**KN**. Yes, I would be able to buy it, but in my buying it there danger because there won’t be anyone to teach me how to use it when I buy it.

**SQT**. Do you think people in your community would use HIVST kits?

**KN**. If they first get educated on them then they will use them

**SQT**. What would motivate them to use the HIVST kit?

**KN**. Firstly it’s knowing that only they will know their results, so in that way it’s private.

**SQT**. What population do you think would benefit from using HIVST kits for testing for HIV?

**KN**. It’s the males because they do not like to test at clinic so I think they would be happy for something like this, but again on their testing alone they could end up committing suicide because a person will find results they did not expect, but ultimately they will gain.

**SQT**. If you believe HIVST is not acceptable, why do you think it is not acceptable and what can be done to make it acceptable?

**KN**. When I think of it, could be that you will test alone and no one knows what decision you could take after that and people need to be taught first, unlike at the clinic you go to a person who knows how its done already and they can advise you.

**SQT.** What are possible concerns/barriers associated with HIVST?

**KN**. Others will be scared to prick themselves, others will fear that once they know that they will have stress.

**SQT**. How do you think people in your home and community would respond to finding out that you are using an HIVST kit?

**KN**. I think they would be happy to get their own HIVST kits.

**SQT**. Would you encourage/ motivate people to test using HIVST kit within your family, your friends, and community members?

**KN**. Yes if they are taught first on how to use the kits then I would encourage them.

**SQT**. How important do you think HIV counselling is for HIV testing?

**KN**. It’s very imported, because when you come here you already know your ways sexually but you do not tell yourself what the result be will for sure. So after counselling I know for sure what to do moving forward.

**SQT**. would written counselling as part of instructions be adequate?

**KN**. Reading something is not the same as being spoken to by a person, maybe there will be more you will be more that you will tell me like my cd4 count the paper can’t tell me that but here you can.

**SQT**. What do you think you would do if you tested positive for HIV using HIVST kit?

**KN**. I would have cried, I don’t know but I would have felt bad, I could have even fainted, you don’t tell yourself that it can be that way. I would have felt bad.

**SQT.** What do you think you would do if you tested negative for HIV using HIVST kit?

**KN**. I would have been happy

**SQT.** What do you think your community reaction would be if they found out you testing using HIVST kit?

**KN**. I think they would also like it, because I don’t think there’s people that do not want to know their status, it’s just the difficulty of going there.

**SQT**. Do you think there would be any social harms associated with HIVST?

**KN**. Yes

**SQT**. If yes, what type of social harm would be encountered? ii. If no, why not?

**KN**. Thing is like you don’t know what a person will think after testing, another person could think to kill them self if the result is positive or if they do not have adequate information.

**7. General:**

**SQT**. Do you think HIVST would have any impact of health seeking behaviour of participants?

**KN**. Yes there is, after you test you think of ways to take better care of yourself sexually, you then know that you must use a condom always.

**SQT.** Would people access prevention or treatment options sooner if they tested for HIV sooner?

**KN**. Yes, I think so because now you get given treatment sooner unlike before when you had to wait for your CD4 count to reach a certain level before you could access treatment.

**SQT**. Do you think people would change their risky sexual behaviour if they tested for HIV using HIVST?

**KN**. I think they could, because when you find out that things are not so well with your health I think the caregiver would have told you how to carry yourself.

**SQT**. What are possible barriers that would be associated with HIVST?

**KN**. Where would we dispose of them after using them?

**SQT**. What do you think would be possible advantages of using HIVST to test for HIV?

**KN**. It would be that people will be happy to use them because of the privacy no one else will know and you do it when you want to, no one forces you. So it’s easy to then go seek treatment because you know that if you get such a result then what the next step is.

**Participant: 0008**

**INTERVIEW 1: HCT/HIVST**

**SQT**. Have you tested for HIV?

**TTM**. Yes I have

**SQT.** what made you test?

**TTM**. It’s because I wanted to get circumcised

**SQT**. What made you decide to test today?

**TTM**. It’s because now I want to always know my status

**SQT**. What is your testing routine?

**TTM**. I would say that at the moment it has been a while since I last tested.

**SQT**. How big of an issue do you think HIV/AIDS is for people in your community and what makes it an issue of greater or lesser importance?

**TTM**. The way I see it they don’t see it as an issue, but I see it as a big issue.

**SQT**. What makes you say that they see it as an issue?

**TTM**. It’s t way they are behaving and being careless sexually

**SQT**. Has the importance of HIV/AIDS changed in the community over the past few years and if so why?

**TTM**. I would say the importance of it has changed because even the deaths it’s no longer the same, it’s unlike in the past.

**SQT**. Is it more of an issue for some groups than for others, and if so, which groups and why?

**TTM**. I would say the group of 20 to 30 year olds

**SQT**. Is there denial or do they accept that AIDS is an issue?

**TTM**. People accept that there is an Aids issue.

**SQT**. What is the reputation of the HIV testing locations in your community?

**TTM**. Ay they are not good the lines are differentiated, so it’s well known that if you are standing in a certain line what it is that you have come to do

**SQT.** Is testing fully confidential?

**TTM**. I’m not sure

**SQT**. What are providers like?

**TTM**. I think they are all right

**SQT**. What do you think are the greatest barriers to HIV testing in your community?

**TTM**. Ay I wouldn’t know, but I will say that most people test only when they get sick, only then do they want to know where they stand in terms of their status.

**SQT.** Are people’s attitudes and beliefs about HIV barriers?

**TTM**. Ay no

**SQT.** What structural factors (e.g. availability of services, lack of resources, lack of privacy in the community, etc.)?

**TTM**. No, I don’t think so because this clinic is not that far you can reach it, and the infrastructures are all right.

**SQT**. What do you know about HIVST?

**TTM**. I know that you either get a positive or a negative result

**SQT**. Where did you hear about HIVST?

**TTM**. It’s my first time hearing about it here

**SQT**. What do you perceive as possible challenges to HIVST?

**TTM**. Lack of counselling, and if they test positive it’s then the fear of coming to the clinic to start on treatment, its better with a counsellor they encourage you to take pills. With HIVST can hide their status while knowing that they are HIV positive.

**SQT.** What do you think would be the benefits of HIVST?

**TTM**. The benefit would be that they then are able to give themselves time to disclose and know which person they will tell first, and not having the pressure of counsellor so and so knows so they then feel pressured to disclose they status although they may not necessarily be ready.

**INTERVIEW 2: HCT/HIVST**

**SQT**. What did you think about HIV testing using this method? Describe your experience.

**TTM**. It was quiet easy and is a process I can get used to over time, I see HIVST as being quiet all right.

**SQT**. What would motivate you to test again for HIV using this method?

**TTM**. Maybe if I have engaged in risky sex or if the condom bursts.

**SQT**. What would discourage you from testing for HIV using this method?

**TTM**. I don’t see what could discourage me

**SQT**. What were challenges you experienced or perceived challenges using this HIV testing method?

**TTM**. It’s just that I had not received any counselling I wasn’t ready, so I don’t know how it would have gone had my results been positive.

**INTERVIEW 3: HCT/HIVST**

**SQT**. After testing through HCT and HIVST, which method do you prefer?

**TTM**. I prefer HIVST

**SQT.** What makes you prefer this method?

**TTM**. It’s because my test results will only be known by me.

**SQT.** What discouraged you from the method you did not choose?

**TTM**. It’s because they might put me on ARVs whilst I’m still not ready.

**SQT.** Which HIV testing method would increase uptake of HIV testing?

**TTM**. Its Hivst it would encourage them to know their status.

3**. Usability and Distribution of HIVST kits:**

**SQT**. If you were to obtain a HIVST kit, where would you store it for use?

**TTM**. In a safe place in the house where I know it will not be seen by another person, under bed where it is hidden

**SQT.** How/where would you dispose of used HIVST?

**TTM**. In the toilet or burn it.

**SQT.** Where would you conduct your HIVST?

**TTM**. In my bedroom.

**SQT.** How much education and training do you think you would need before you can do the HIVST on your own?

**TTM**. It’s a lot because you must be counselled before you test prior to you feeling ready.

**SQT**. What type of education and training would you require?

**TTM**. It would be pamphlets and videos that show how one goes about testing.

**SQT**. Do you think community members will require training before scale-up of HIVST?

**TTM.** Yes, they require it a lot.

**SQT**. What information should be included in instructions?

**TTM**. Nothing needs to be added it seems complete to me, because when you follow the instructions you then get a result at the end.

**SQT.** What do you perceive as common errors that could be made when using the HIVST kit?

**TTM**. It would be maybe that the person could put both buffer and blood in the same hole instead into the different holes, or use to little or too much buffer

**SQT.** What would be the ideal distribution point for HIVST kits?

**TTM**. Clinic where they display the condoms

**SQT.** would you access HIVST kits if you had to pay for them?

**TTM**. Yes

**SQT**. Why?

**TTM**.I think it is something I could afford

**4. Acceptability of HIVST:**

**SQT.** Do you think people in your community would use HIVST kits?

**TTM**. Yes, they would

**SQT**. What would motivate them from using the HIVST kit?

**TTM**. Well because there are those who do not know their status, and that some are afraid to go test at the local clinic.

**SQT**. What would motivate/discourage you from using HIVST kit?

**TTM**. I would say it would be those that are scared to know their status

**SQT**. What population do you think would benefit from using HIVST kits for testing for HIV?

**TTM**. It’s the teenagers because they are not fully educated on HIV.

**SQT**. If you believe HIVST is not acceptable, why do you think it is not acceptable and what can be done to make it acceptable?

**TTM**. I just think this kit is good.

**SQT**. What are possible concerns/barriers associated with HIVST?

**TTM**. I would think fear and just not being ready to test using the kit.

**SQT**. How do you think people in your home and community would respond to finding out that you are using an HIVST kit?

**TTM**. Maybe some of them would also like to use the kit to test themselves and some would be scared because people are not the same and could need to be counselled first.

**SQT**. Would you encourage/ motivate people to test using HIVST kit within your family, your friends, and community members?

**TTM**. Yes I would encourage them.

**5. Need for HIV counselling:**

**SQT**. How important do you think HIV counselling is for HIV testing?

**TTM**. Its important because it could happen that some people could kill themselves, if they find they are positive, because you don’t know how every person would react to a HIV positive result so counselling is important.

**SQT**. would written counselling as part of instructions be adequate?

**TTM**. I think it would be enough because the time you read the counselling you are encouraged to test, maybe the result is not what you expected the counselling will give you motivation moving forward.

**6. Social Harms associated with HIVST:**

**SQT**. What do you think you would do if you tested positive for HIV using HIVST kit?

**TTM**. I would try find someone I trust and disclose my status to them, then go to the clinic so I can start with the ARVs.

**SQT**. What do you think you would do if you tested negative for HIV using HIVST kit?

**TTM**. Then I would take care when it comes to my sexual life, and test more regularly.

**SQT**. What do you think your community reaction would be if they found out you testing using HIVST kit?

**TTM**. I think some would like to join me, and also test themselves in the comfort of their homes,

**SQT**. Do you think there would be any social harms associated with HIVST?

**TTM**. There could be social harms for instance, I could get this kit and then go and force another person to test or even a partner, or maybe even an beat them up if they refuse to test

**7. General:**

**SQT**. Do you think HIVST would have any impact of health seeking behaviour of participants?

**TTM**. Yes

**SQT**. would people access prevention or treatment options sooner if they tested for HIV sooner?

**TTM**. Yes that should be what happens, it would be a good thing and not wait, because now in any case once you test you treat.

**SQT**. Do you think people would change their risky sexual behaviour if they tested for HIV using HIVST?

**TTM**. I don’t think so because most people are educated about aids.

**SQT**. Do you think there would be any stigma or discrimination associated with HIVST?

**TTM**. Maybe they fear or are not ready to know their status

**SQT**. What do you think would be possible advantages of using HIVST to test for HIV?

**TTM.** I think by knowing your status you can change your ways, and be well behaved sexually.

**Participant: 0028**

**INTERVIEW 1: HCT/HIVST**

**SQT**. Have you tested for HIV?

**NG.** Yes

**SQT**. If yes, what made you test?

**NG.** I just wanted to know my status

**SQT**. What made you decide to test today?

**NG.** It’s a requirement for this study

**SQT**. Routine 3 monthly testing?

**NG.** I test myself at work, I’ve tested maybe 4 time in the past year.

**SQT**. How big of an issue do you think HIV/AIDS is for people in your community and what makes it an issue of greater or lesser importance?

**NG.** It’s important, it’s just that the stigma that was there before is no longer there, there’s an acceptance and knowledge of which channels to follow to get treatment.

**SQT**. Has the importance of HIV/AIDS changed in the community over the past few years and if so why?

**NG.** The importance has not changed people are also aware of the opportunistic infections so if one has TB they also test for HIV.

**SQT**. Is it more of an issue for some groups than for others, and if so, which groups and why?

**NG**. It’s an issue for everybody.

**SQT**. Is there denial or do they accept that AIDS is an issue?

**NG.** There is acceptance.

**SQT**. What is the reputation of the HIV testing locations in your community?

**NG.** Its ok it’s just that they divide the people and give different coloured stickers for different places, so everyone knows that if you have a green sticker that means that you are going to fetch your treatment. There’s no secret as to what you came to do.

**SQT**. Is testing fully confidential?

**NG.** Yes

**SQT.** What are providers like?

**NG.** They are ok, I have worked with them and found them to be ok.

**SQT**. What do you think are the greatest barriers to HIV testing in your community?

**NG.** I think its fear, and when you go get your treatment everyone at the clinic will see that you came to get HIV treatment**.** So people end up not testing at the clinics nearest to them due to that fear.

**SQT**. What structural factors (e.g. availability of services, lack of resources, lack of privacy in the community, etc.)?

**NG.** That could also be a factor because the clinic is quite small and is surrounded by bushes and skwatta camps, so there is a lot of robbery that happens around there**.**

**SQT**. What do you know about HIVST?

**NG**. I already knew that its effective and you can trust the results.

**SQT**. Where did you hear about HIVST?

**NG.** I heard in my class 2 weeks back.

**SQT**. What do you perceive as possible challenges to HIVST?

**NG.** For instance you get one kit, and you get home and put the samples in the wrong holes.

**SQT**. What do you think would be the benefits of HIVST?

**NG.** It keeps your business private if you are the kind of person that like to keep your results secret.

**INTERVIEW 2: HCT**

**SQT**. what did you think about HIV testing using this method? Describe your experience.

**NG**. I see it being good because the counselling gives you the different methods of coping.

**SQT**. What would motivate you to test again for HIV using this method?

**NG.** I’m not sure because I’m so used to testing myself, maybe if I was testing with my boyfriend I would test like this again.

**SQT**. what would discourage you from testing for HIV using this method?

**NG**. I do not know, I have no issues with the method.

**SQT**. What were challenges you experienced or perceived challenges using this HIV testing method?

**NG**. I do not perceive any**.**

**INTERVIEW 3: HCT/HIVST**

**SQT**. After testing through HCT and HIVST, which method do you prefer?

**NG.** HIVST

**SQT**. What makes you prefer this method?

**NG.** Because I can know myself first where I stand with regards to my status.

**SQT**. Which HIV testing method would increase uptake of HIV testing?

**NG.** HIVST, but what is better is HCT because they receive the relevant pre and post-test counselling.

**3. Usability and Distribution of HIVST kits**.

**SQT**. If you were to obtain a HIVST kit, where would you store it for use?

**NG.** In my cosmetics drawer.

**SQT**. How/where would you dispose of used HIVST?

**NG.** In the bin

**SQT**. Where would you conduct your HIVST?

**NG.** In my bedroom

**SQT**. How much education and training do you think you would need before you can do the HIVST on your own?

**NG.** I think I would first need to go through counselling so I know how to proceed and the emotions I may feel.

**SQT**. Do you think community members will require training before scale-up of HIVST?

**NG.** Yes, because I do not think doing this test will be simple for everyone.

**SQT**. What information should be included in instructions?

**NG.** To add isiZulu as well, many in the township do not understand English.

**SQT**. What do you perceive as common errors that could be made when using the HIVST kit?

**NG.** Like the buffer liquid it is meant to be kept in a cool place, a person could put it in a hot place and then receive results that are not accurate**.**

**SQT.** What would be the ideal distribution point for HIVST kits?

**NG.** Supermarkets, next to the tills that way it’s easily accessible**.**

**SQT.** Would you access HIVST kits if you had to pay for them

**NG.** No it has to be free, yes I would buy it.

**SQT.** Yes, why?

**NG.** Because I think government would fund them so they could be cheaper.

**4. Acceptability of HIVST**:

**SQT**. Do you think people in your community would use HIVST kits?

**NG.** Yes.

**SQT**. What would motivate/discourage them from using the HIVST kit?

**NG.** Privacy would encourage them.

**SQT**. What population do you think would benefit from using HIVST kits for testing for HIV?

**NG.** I think it’s the youth, high school all the way to 25 years ,they are busy and do not have the time to go to these testing centres.

**SQT**. If you believe HIVST is not acceptable, why do you think it is not acceptable and what can be done to make it acceptable?

**NG.** Maybe the question of how is this test different to the one they use at clinics? Is it more effective, I think providing education on the kit can address that issue.

**SQT.**  How do you think people in your home and community would respond to finding out that you are using an HIVST kit?

**NG.** They would have no issue they could even ask for me to bring them some kits**.**

**SQT**. Would you encourage/ motivate people to test using HIVST kit within your family, your friends, and community members?

**NG**. Yes.

**5. Need for HIV counselling**:

**SQT**. How important do you think HIV counselling is for HIV testing? i. What makes it important/not important?

**NG**. It’s so important especially for those testing for the very first time, it puts you at ease and the process to follow is explained fully, like the window period and so on.

**SQT.** Would written counselling as part of instructions be adequate? i. If yes/no, why

**NG**. No it would not be enough, they would need to go out into the communities and teach them about HIV because people are not paying attention to HIV anymore. Then they can do the roll out of the kits with the written counselling.

**6. Social Harms associated with HIVST:**

**SQT**. What do you think you would do if you tested positive for HIV using HIVST kit?

**NG.** I would be in shock, then denial for a while then go to the clinic.

**SQT**. What do you think you would do if you tested negative for HIV using HIVST kit?

**NG.** I would dispose of the kit.

**SQT**. What do you think your community reaction would be if they found out you testing using HIVST kit?

**NG.** Some would talk because they love to judge, and judge me why I’m so bothered with my health, am I behaving badly?

**SQT**. Do you think there would be any social harms associated with HIVST? If yes, what type of social harm would be encountered?

**NG.** One could get angry and go kill their boyfriend suspecting that they could have infected them and come to find that the person had tested using this kit.

**7. General**:

**SQT**. Do you think HIVST would have any impact of health seeking behaviour of participants?

**NG.** Oh yes

**SQT**. Would people access prevention or treatment options sooner if they tested for HIV sooner?

**NG.** Yes

**SQT**. Do you think people would change their risky sexual behaviour if they tested for HIV using HIVST?

**NG**. People will always be people, Kit or no kit it will be up to the individual if they want to change or not.

**SQT**. Do you think there would be any stigma or discrimination associated with HIVST?

**NG.** There could be.

**SQT**. What are possible barriers that would be associated with HIVST?

**NG.** Fear of the results mostly and maybe if the kit is expensive that too could be a barrier, and fear of being spotted by someone you know when you go purchase the kit.

**SQT**. What do you think would be possible advantages of using HIVST to test for HIV?

**NG.** It’s also convenient for couples, you can go at any time with your partner to buy it and use in private.

**Participant: 0001**

**INTERVIEW 1**

**PK:** Have you tested for HIV?

**DMN**: yes

**PK**: What made you test?

**DMN:** I was seeing many people being infected with HIV

**PK**: What made you decide to test today?

**DNM**: My reason is, because I want to know my status

**PK:** How often do you came test?

**DNM:** I test when I come to the research study, otherwise I do not come

**PK**: How big of an issue do you think HIV/Aids is for the people in your community and what makes it an issue of greater or lesser importance?

**DNM**: Aw people don’t care, because we know that if we get infected we will get the ARV’s

**PK:** so the reason people don’t care is, even if they get infected they know they will get ARV’s so its fine?

**DNM**: yes

**PK**: Has the importance of HIV changed in the community in the past few years if so why?

**DNM**: The change I see has been since the start of the research study here at Caprisa

**PK:** So how did this study help?

**DNM**: It helped because when we were using the gel, there were those who come here and used the gel, then if their partner got the virus (HIV), then the one that uses the gel doesn’t get infected.

**PK:** when you look at it in general for which groups within your community is HIV an issue?

**DNM:** It’s a big problem for the older people, but with the younger people even if it is a problem for them they do not try and show that there is a problem.

**PK:** So if you say older people do you mean mothers and grandmothers?

**DNM:** Yes mothers and grandmothers

**PK:** so how is it a problem to them?

**DNM:** well in their hearts they believe that this is a disease for the young so when they get infected it’s then a problem.

**PK:** so they do not see themselves being infected because this is an illness for the young?

**DNM:** Yes

**PK**: is there denial or do they accept that AIDS is an issue?

**DNM**: yes there people in denial they say its cancer and diabetes that are now a problem, they say Aids is no longer a problem.

**PK:** What is the reputation of the HIV testing locations in your community do you test at Mafakatini clinic?

**DNM:** I do go to the Mafakathini clinic but not to test.

**PK**: So since you test at Caprisa what is the reputation Caprisa has within the community in terms of HIV resting?

**DNM**: Well here at Caprisa in the past sick people would come, those with the HIV virus, and it was nice because you knew that if you test here and find that you have HIV then you get treatment easily. It is a place well known for treating the virus.

**PK**: This site is well known for treatment and how about HIV testing?

**DNM**: The people that test you here Test you nicely it’s not the same as at the Mafakatini clinic. The workers here are better than the ones at the clinic, which is why we like to test here.

**PK**; In terms of privacy how do about your results, is it confidential

**DMN**: No your information is confidential, information is safe

**PK**: The way you see it what are the greatest barriers to HIV testing in your community

**DNM**: It’s because people talk at the clinic some of the stuff are our neighbours, you are then scared of that, but its better here (Caprisa), here even if you do have neighbours it’s not the same as the clinic

**PK:** In this area is there places where one can test

**DNM:** It’s just here and at the clinic but at the clinic you go there when you’ve become really sick. You don’t just go

**PK**: Do you know about HIVST?

**DNM**; No

**PK:** where did you hear about HIVST?

**DNM**: was my first time hearing from you.

**PK:** what do you perceive as the possible challenges to HIVST?

**DNM:** Maybe when I see that I am infected I may not really believe the results

**PK**: what do you think are the benefits of testing yourself

**DNM**: I don’t see the benefits like I said I may not believe the results.

**Participant: 0002**

**INTERVIEW 1**

**SQT:** Have you tested for HIV?

**ZAN**: yes I’ve been testing here since 2008, even if I’m not in any study I still go and test, 2 weeks back I went to test.

**SQT:** If yes, what made you test

**ZAN**: hmmm what could I say, my starting studies here at Caprisa made me realise how important life is. So even when I’m not engaging in sex. It is in my blood I like to test, even when the 3 months are not up, its encouraging, I would say it’s the type of clinic, it’s not about Caprisa working but it’s about our lives. Health seeking behaviour, HIV status awareness

**SQT**: What made you decide to test today?

**ZAN:** what would I say, It’s because I have no fear, and I love my life, and I was happy when I got a call from Caprisa because it encourages you to take care of your life, I have no problem I have no fear, which made it easy for me to test today.

**SQT:** what is your testing routine?

**ZAN**: when I was in a research study with Caprisa we tested every month, I test maybe 6 times a year, Ja

**SQT**: How big of an issue do you think HIV/AIDS is for people in your community and what makes it an issue of greater or lesser importance?

**ZAN**: I would say you can see that it is a problem, but then people are scared. Most of them are not open, and you find that a person finds out they are HIV positive but they do not disclose. Sometimes you find that it’s only when the person starts losing weight then only then do they start speaking up, and others do get the treatment but have nothing to eat. Others are scared because they know that they are not using protection. So a person has shame to say. It’s a problem and there’s still stigma associated with it.

**SQT**: Has the importance of HIV/AIDS changed in the community over the past few years and if so why?

**ZAN**: No people’s attitudes are now a bit better, because some are able to disclose. It’s not the same as before because a person would even change their clinic and you find the person does not use that clinic, they use the clinics in town, because the person would be scared to associate with their community to even que in local clinics for ARV’s .it’s not the same now most of them are able to gather at the clinic and discuss ways to take care of themselves.

**SQT**: Is it more of an issue for some groups than for others, and if so, which groups and why?

**ZAN**: Ja it’s a big problem in the youth, because they do not use protection, so if they find out their HIV positive, what will people say.

**SQ**T: Is there denial or do they accept that AIDS is an issue?

**ZAN:** Even though there is some denial it’s not like before, it was a problem before to accept that it’s a disease that’s real. Right now there is a sense of HIV is real and you have to protect yourself.

**SQT:** What is the reputation of the HIV testing locations in your community?

**ZAN**: some clinics here are all right, even though especially this one Caprisa, the reputation is not right because even with the gel, they (people) know that it has HIV on it. If your partner knows that you use the gel even the relationship can end because you go to Caprisa. So until the person gets more information from the Caprisa stuff, there’s stigma that a person who goes to Caprisa is HIV positive.

**SQT:** Is testing fully confidential? Explain.

**ZAN**: Ja it is confidential, I’ve never heard that a stuff member has spoken about a patient, I think they keep it private. I think its people outside the gate that undermine and spread gossip if they see you leave Caprisa

**SQT:** What are providers like?

**ZAN:** The providers are all right , there’s communication but you find at times a lot of us come to the clinic and you find that they work to push the job. The providers work well and have knowledge.

**SQT:** What do you think are the greatest barriers to HIV testing in your community?

**ZAN:** fear, and also knowing that they did not use protection, or they cheated with a person whose status they do not know, so because of that a person ends up not going to test and you find that the virus is doing at it pleases in their body and they go for testing when its late and their body is showing signs of HIV or are sick.

**SQT**: What structural factors (e.g. availability of services, lack of resources, lack of privacy in the community, etc.)?

**ZAN:** In our community its issues with transport, poverty people don’t have jobs and if they miss their transport they have nothing to eat while waiting for the next transportation. These factors cause the persons health to deteriorate.

**SQT**: What do you know about HIVST?

**ZAN:** To self-test you need to be brave, and know that you need to counsel yourself should the results be positive, you must know it is not the end of your life, you need to find a way to carry on with life. Self-testing calls for a person to be able to accept the situation as it is. And be brave even if the needle is sore.

**SQT**: Where did you hear about HIVST?

**ZAN**: today is the first time I hear about HIVST

**SQT:** What do you perceive as possible challenges to HIVST?

**ZAN:** The problem could be if the person receives results they were not expecting, that would lead to issues because we do not all respond to bad news the same way. Which could lead to an increase in people harming themselves because of this self-testing.

**SQT** What do you think would be the benefits of HIVST

**ZAN:** Not being worried about having to go to the clinic to test if you have not been in a while, you can save on transport money, then use that money to buy healthy food, even though the result can be positive it depends on how the mind-set of the person using the test is, But I think it would be a benefit.

**Participant: 0003**

**PVK:** If I may ask you, have you tested for HIV?

**V003**: Yes

**PVK:** Please speak louder so that I can hear your response

**V003**: Yes

**PVK:**  What made you get test for HIV?

**V0003**: I always want to know my status.

**PVK:** Okay, so today what makes you want to get tested?

**V0003**: I don’t see why I should not get tested today because it has been more than a month since I last tested.

**PVK:** Okay. How often do you normally get tested for HIV?

**V0003**: I get tested every month.

**PVK:** When last did you get tested?

**V003**: I last tested in September?

**PVK:** Where do you usually go for HIV testing?

**V0003:** I get tested here at the clinic.

**PVK:** Here at Mafakatini?

**V0003:** Yes

**PVK:** Ok**.** How big of an issue do you think HIV is for people in your community?

**V0003:** It is a big problem**.**

**PVK:** How so? What are the things that makes you think it is a big problem?

**V0003:** Because there are so many people who are already infected, who don’t condomise.

**PVK:** When it comes to HIV/AIDS in your community, do you think the importance of HIV has changed over the past few years or maybe there is no change?

**V0003:** For me I think there is no change because even before, people were counselled about HIV, there was education in schools, in the community but still there are so many people who are getting infected with the virus. For me, I think people don’t listen to what they are being taught about.

**PVK:** Ok. Which group do you think is more affected by HIV than others? For an example, older group or younger group. Which group do you think it is more of an issue for them than others, and if so why do you think so?

**V0003:** I think it is more of an issue for youth.

**PVK:** Why do you think so?

**V0003:** Because they are the ones infected with HIV the most.

**PVK:** Do you think there is a denial in your community that AIDS is an issue? Have people accepted or they have not accepted that this is an issue in the community?

**V0003:** They do accept that there is AIDS in the community.

**PVK:** What is the reputation of HIV testing facilities that you use in your community? You said you use Mafakatini clinic because they provide HIV testing, what is the reputation of this clinic? How do people feel about doing HIV testing in Mafakatini?

**V0003:** The reputation of Mafakatini clinic is good because whatever they do is kept confidential. You don’t hear people talking about people who got tested and about their results. It is kept as a secret.

**PVK:**  How are the people who provide HIV counselling like?

**V0003:** There are good people. They are friendly and they are open. They are not intimidating even if you enter the room, they smile.

**PVK:** What do you think is the reason people don’t use their local clinics for HIV testing?

**V0003**: I think it is being coward. People are scared that maybe they are already infected. Sometimes some of them are already infected and they are scared to know the results because they don’t know how to handle the situation.

**PVK:** In terms of location, for example, Mafakatini clinic, is it easy for people to access this clinic for HIV testing or it is difficult for some because of different reasons, for example, maybe it is far for some people, or maybe there are other reasons?

**V0003:** This clinic is better and sometimes they do have mobile clinics once a month. If people are lazy to come to the clinic or they feel it is far, they can go to the mobile clinic to get tested.

**PVK:** Ok. Do you know anything about HIVST?

**V0003**: Yes

**PVK:** So you knew about it even before I explained to you earlier?

**V0003**: No, I did not know about it. It was the first time I hear about it when you told me today.

**Participant: 0004**

**PVK:** Have you tested for HIV?

**E004**: Yes

**PVK:**  What made you get tested for HIV?

**E0004**: It is because I always want to know my status.

**PVK:** Okay, so as I have told you that today you are going to get tested, what made you agree to get tested today?

**E0004**: It is because I like getting tested for HIV.

**PVK:**  How often do you get tested for HIV?

**E0004**: After every 3 months.

**PVK:** Ok**.** How big of an issue do you think HIV is for people in your community? If it is an issue.

**E0004:** I am not sure.

**PVK:** You don’t know if it is an issue or not?

**E0004:** I don’t know.

**PVK:** Do you think the importance of HIV has changed in your community over the past few years?

**E0004:** Yes, there is a change. Things are not as they used to be. People are not scared of HIV anymore.

**PVK:** The change is that people are not scared of HIV anymore.

**E0004**: Yes

**PVK:** Do you think HIV is more of an issue for certain groups? Which group do you think is more affected by HIV than others?

**E0004:** It is more on an issue for older people.

**PVK:** Why do you think so?

**E0004:** Older people are afraid of AIDS. For younger people, it is easy for them to accept HIV. Older people are still scared.

**PVK:** Do you think there is a denial in your community that AIDS is an issue?

**E0004:** Not that I know of. I don’t think so.

**PVK:**  You think people have accepted that AIDS is an issue?

**E0004**: Yes most of them have accepted now.

**PVK:** Where do you usually go for HIV testing?

**E0004**: At the clinic.

**PVK:** Your local clinic?

**E0004**: Yes, at Illovu C. Clinic.

**PVK:** What is the reputation of HIV testing facilities that you use in your community?

**E0004:** They are good people.

**PVK:** Is there a full confidentiality there?

**E0004**: Yes but people are scared to go to that room.

**PVK:** Which room?

**E0004**: Because everybody knows that when you go to room number 7 you are going for HIV testing.

**PVK:** How are the people who provide HIV testing like?

**E0004:** There are good people.

**PVK:** Are they open and friendly?

**E0004**: Yes, they are friendly and they are open. You can easily talk to them.

**PVK:** What do you think is the reason people don’t like to use their local clinics for HIV testing?

**E0004**: I think they may be scared to go to that room because there is only one room for HIV testing. Everybody knows that room number 7 deals with HIV and AIDS. Some people don’t understand that going to that room does not necessary mean you are already HIV positive. Sometimes people go there just to get information.

**PVK:** Do you think people’s attitudes and beliefs are barriers to HIV testing or are the the reasons people don’t get tested?

**E0004**: We don’t have same beliefs, people have different beliefs, so I would not know.

**PVK:** In terms of structural factors, it easy for people in your community to access the local clinic for HIV testing?

**E0004**: Yes, it is easy to access the clinic.

**PVK:** You don’t find yourself going for HIV testing and you are told they don’t have kits to perform HIV test?

**E0004**: No I don’t think so. It has never happened.

**PVK:** Ok. Do you know anything about HIVST?

**E0004**: Which one is that?

**PVK:** As I mentioned that you buy a kit and test yourself at home.

**E0004**: No, I have heard people talking about something like that, that you can buy and do self-testing but I have not seen it, I don’t know how it looks like. I have not bought it.

**PVK:** As you have heard that there is something like that, what do you perceive as a possible challenge to this HIVST kit? Do you foresee any challenges**?**

**E0004:** Maybe people would find it difficult to use it. Maybe they would not be able to prick themselves. – Usability of HIVST kit

**PVK:** Ok, the challenges would be that people would not be able to prick themselves, but what do you think would be the benefits of using this HIVST kit?

**E0004:** If we would be able to get it for free. If you would have it at home for free, it would be a good thing to have. You would be able to use it at any time you want to test yourself for HIV.

**Participant: 0005**

**SQT:** Have you tested for HIV?

**TTM:** Yes, hmmm the first time I took the test it was a must because I had been raped so they had to get me on pills. Since then I’ve been testing. So it’s easier to test if you have tested before. From then I needed to know my status and also the person I was with at the time had many girlfriends.

**SQT**: What made you decide to test today?

**TTM:** Today well, it’s because I need to test, I last tested in July or August and I don’t get the time to test or go to the clinic and I hate clinics, so I decided let me do it, it’s after 3 months, my routine to test is every 6 months or so, or when I remember it has been a while since I got tested and I then test.

**SQT:** How big of an issue do you think HIV/AIDS is for people in your community and what makes it an issue of greater or lesser importance? Has the importance of HIV/AIDS changed in the community over the past few years and if so why?

**TTM:** Ahhhhm I would say maybe before it was an issue, but now they know more about HIV but some do not care, and others don’t care because people will always talk anyway. Ja so I don’t think it is that much of an issue, but if one was to hear that so and so has HIV maybe then you might get a fright because you don’t know anyone personally with HIV

**SQT:** Is it more of an issue for some groups than for others, and if so, which groups and why?

**TTM:** I would say so, I would say the elders because HIV for them is still a frightening thing, with the youth most have it so they don’t care

**SQT**: Is there denial or do they accept that AIDS is an issue?

**TTM:** I would say there in denial because I don’t know, it’s like people don’t care anymore, like HIV is there or not there they get it or they don’t, as I see in my conversations with friends and they tell you that so and so slept with so and so, and you are shocked that these people are still sleeping around without a condom while the situation is like this, you see. I don’t think they believe until they get infected and get sick.

**SQT**: What is the reputation of the HIV testing locations in your community?

**TTM:** Im not that sure in my community because most of the time I test by the doctor.

**SQT:** What makes not test at the community clinic?

**TTM:** Firstly people don’t understand why you go for testing or go to places that have services for HIV. They then think you have HIV. Secondly the nurses are from your community, so when you go there you fear that they will tell people what services you go for at the clinic. Nurses don’t treat you the same.

**SQT**: Is testing fully confidential?

**TTM:** I doubt

**SQT:** Why would you doubt, what makes you doubt?

**TTM:** No I doubt because, people like to talk, especially in the location, there’s so many of us and they visit each other, they do stuff, and you find yourself hearing other things. I have a friend that is a nurse, so I wouldn’t hear from her but I would hear things from a person she had been talking too, like you know so and so why they were at the clinic and telling them things that are private and they not supposed to say such things.

**SQT:** What are providers like?

**TTM:** In HIV testing, I think it’s not the same they have to treat people in a certain way, like I said nurses are not the same, but mostly because I remember when I was pregnant and we were being educated and others being tested, the nurses were all right. I think that the nurses that do HIV testing are told that ‘’you have to be like this’’, so they try but it’s not all nurses that are on that level

**SQT:** What do you think are the greatest barriers to HIV testing in your community?

**TTM:** Hmmm well mostly, Im speaking of the girls, let’s say they slept with a person without using a condom, some are scared of the results and ja what if I got it what will I do?

**SQT:** What structural factors (e.g. availability of services, lack of resources, lack of privacy in the community, etc.)?

**TTM:** Hmmm, maybe I’m not really sure that could also be a factor, because some people when they go there they know what they came to do and are not looking at other things.

**SQT:** What do you know about HIVST?

**TTM:** I’ve never done HIVST, but I think to self-test is private and I think it comes with instructions that you have to do this in a particular way.

**SQT:** Where did you hear about HIVST?

**TTM**: I heard about it here (CAPRISA clinic)

**SQT**: What do you perceive as possible challenges to HIVST?

**TTM:** Firstly, there would be doubt in the results since you do it yourself, or let’s say the results are not what they wanted and then they take it in a bad way, cause there is no counselling close by.

**SQT**: What do you think would be the benefits of HIVST?

**TTM:** It’s easier, because the results stay private, but I do not know how you would then know your cd4 count, if you want counselling you can then go for counselling where you want to.

**Participant: 0006**

**PVK:** Have you tested for HIV before?

**E0006**: As I have mentioned earlier, I have never tested for HIV before.

**PVK:**  What prevented you from getting tested?

**E0006**: What prevented me from testing was fear. I was not confident enough. I did not know how I will cope with the results.

**PVK:**  So today you are going to be tested, what made you decide to agree to get tested today?

**E0006**: Today I realised that I cannot hide forever. I took a courage to get tested because I know eventually I will have to know my status.

**PVK:** Ok**.** How big of an issue do you think HIV is for people in your community?

**E0006:** I would say it is a big issue in the community because most people are already infected and not only are they infected, they are also dying. They don’t want to make use of the clinic to know where they stand in terms of their HIV status. In general, it is important to know your HIV status nowadays.

**PVK:**  Do you think the importance of HIV has changed in your community over the past few years? Do you think HIV/AIDS is still important in your community or it is not important?

**E0006:** I can say there is a change because most people who did not want to get tested are now getting tested because now they now know that there is something to supress the virus in the body. They are now motivated to get tested because they know they will get help. However there are still those that are not willing to know their status due to fear. But as time goes on, there are more and more people coming forward to get tested, that is why I think there is a change.

**PVK:** Do you think HIV is more of an issue for some group, if there is one? For example, does it affect older people, youth, men or women? Which group do you think is more affected by HIV than others?

**E0006:** In most case HIV is more on an issue in youth because of their risk behaviours. They can’t control themselves on most of the things. That is why I think it is an issue for young people. For older generation, it is very rare, not many of them are affected. But there are those few that are affected. Youth is the one that is affected more.

**PVK:** Do you think there is a denial in your community that AIDS is an issue? Or nowadays people do accept that AIDS is an issue in the community?

**E0006:** I don’t think there are people who are still denying that AIDS does not exist. Sometimes when you tell someone about condoms to prevent getting infected, the person would just say “oh don’t worry, people don’t die of AIDS anymore because there are ARVs to control HIV”. So people know there is treatment and they don’t deny AIDS. They accept AIDS as part of life.

**PVK:** What do you think are the greatest barriers to HIV testing in your community? Something that prevent people from getting HIV testing?

**E0006**: I am going to talk about myself. The reason I was scared to go for HIV testing is that I did not trust myself. I have that feeling of how I am going to deal with it, if I have to find out of that I am positive.

**PVK:** Ok. Do you know anything about HIVST? Have you heard about it before? Or it is the first time you heard about something like?

**E0006**: It is the first time I am hearing about this that people can perform HIV self-testing. I have heard that people do get HIV testing but they go to the clinic. I know that you can ask the counsellor to test you but not about testing yourself.

**PVK:** What do you perceive as a possible challenges to this HIVST kit?

**E0006:** They could be challenges because they say if you go for HIV testing, you have to get counselling first before you get tested. You need someone to talk to, someone to make jokes, someone to prepare you and make you feel comfortable and tell you what to expect. The challenge is that if you do self-testing, no one would prepare you. You would be on your own. Even if you have a kit, you can decide not to go ahead with the test because no one is advising you and motivating you, giving you courage to go ahead with the test. You need someone to tell you that life goes on no matter what the results are. But at the same time, as you mentioned that this is something that you can use at home on your own, for people who are busy, working most of the time, even if you have time off, you have to do house chores, you can’t go to the clinic, because some clinics do not open on Sundays, having this kit would help because you can use it, especially those who are prepared to know their status and those that are brave, just like me. I would use this test because I am brave. It is good for people who are brave and who are willing to know their status but are unable to go to clinic.

**Participant: 0007**

**SQT:** Have you tested for HIV?

**DS**: Yes I’ve tested

**SQT:** If yes, what made you test?

**DS:** It’s because there was no trust with the father of my child and I.

**SQT:** What made you decide to test today?

**DS**: It’s the problems I’ve faced in the past with the father of my child, he (Avela) has cheated on me so I didn’t know what was going on with me, so wanted to know my status

**SQT**: what is your testing Routine?

**DS**: This year I’ve tested 3 times

**SQT:** How big of an issue do you think HIV/AIDS is for people in your community and what makes it an issue of greater or lesser importance?

**DS**: Its seems to be an important issue

**SQT:** Has the importance of HIV/AIDS changed in the community over the past few years and if so why?

**DS:** It is more of a problem now

**SQT:** Is it more of an issue for some groups than for others, and if so, which groups and why?

**DS**: It’s the teenagers

**SQT:** Is there denial or do they accept that AIDS is an issue?

**DS**: Others don’t take it seriously

**SQT:** What is the reputation of the HIV testing locations in your community?

**DS:** Ay people say there’s no issues they are all right

**SQT:** Is testing fully confidential?

**DS**: Yes

**SQT:** What are providers like?

**DS**: They are all right people

**SQT:** What do you think are the greatest barriers to HIV testing in your community?

**DS**: I think it’s because they are scared I don’t know

**SQT:** Are people’s attitudes and beliefs about HIV barriers?

**DS:** Ja maybe they are scared

**SQT:** What structural factors (e.g. availability of services, lack of resources, lack of privacy in the community, etc.)?

**DS**: No, structural factors are all right

**SQT:** What do you know about HIVST?

**DS**: That I have to know my status

**SQT:** Where did you hear about HIVST?

**DS**: It’s just I’ve never tested myself, I heard about it at the hospital

**SQT:** What do you perceive as possible challenges to HIVST?

**DS**: I don’t know

**SQT:** What do you think would be the benefits of HIVST?

**DS**: It’s that if you are positive your status remains with you don’t go around telling people

**Participant: 0008**

**SQT:** Have you tested for HIV?

**TTM:** Yes I have

**SQT:** what made you test?

**TTM:** It’s because I wanted to get circumcised

**SQT:** What made you decide to test today?

**TTM:** It’s because now I want to always know my status

**SQT:** What is your testing routine?

**TTM:** I would say that at the moment it has been a while since I last tested.

**SQT:** How big of an issue do you think HIV/AIDS is for people in your community and what makes it an issue of greater or lesser importance?

**TTM:** The way I see it they don’t see it as an issue, but I see it as a big issue.

**SQT**: What makes you say that they see it as an issue?

**TTM**: It’s t way they are behaving and being careless sexually

**SQT**: has the importance of HIV/AIDS changed in the community over the past few years and if so why?

**TTM:** I would say the importance of it has changed because even the deaths it’s no longer the same, it’s unlike in the past.

**SQT:** What is the reputation of the HIV testing locations in your community?

**TTM:** Ay they are not good the lines are differentiated, so it’s well known that if you are standing in a certain line what it is that you have come to do

**SQT**: Is testing fully confidential?

**TTM**: I’m not sure

**SQT**: What are providers like?

**TTM**: I think they are all right

**SQT**: What do you think are the greatest barriers to HIV testing in your community?

**TTM**: Ay I wouldn’t know, but I will say that most people test only when they get sick, only then do they want to know where they stand in terms of their status.

**SQT**: Are people’s attitudes and beliefs about HIV barriers?

**TTM**: Ay no

**SQT**: What structural factors (e.g. availability of services, lack of resources, lack of privacy in the community, etc.)?

**TTM:** No I don’t think so because this clinic is not that far you can reach it, and the infrastructures are all right.

**SQT**: What do you know about HIVST?

**TTM:** I know that you either get a positive or a negative result

**SQT**: Where did you hear about HIVST?

**TTM:** It’s my first time hearing about it here

**SQT**: What do you perceive as possible challenges to HIVST?

**TTM:** Lack of counselling, and if they test positive it’s then the fear of coming to the clinic to start on treatment, its better with a counsellor they encourage you to take pills. With HIVST can hide their status while knowing that they are HIV positive.

**SQT**: What do you think would be the benefits of HIVST?

**TTM:** The benefit would be that they then are able to give themselves time to disclose and know which person they will tell first, and not having the pressure of counsellor so and so knows so they then feel pressured to disclose they status although they may not necessarily be ready.

**Participant: 0009**

**PVK:** Have you tested for HIV before?

**V0009**: Yes

**PVK:** What made you get test for HIV?

**V0009**: First, I tested because I was going for MMC. The last time I tested because I had small rash all over my body that I did not understand, so I decided to get tested for HIV.

**PVK:** In total, how many times have you been tested for HIV, if you can remember?

**V0009**: Many times, maybe 4 times, I think.

**PVK:**  Okay, so today what makes you agree to get tested?

**V0009**: When I last tested, my status was right (negative). I am not scared to get tested again.

**PVK:** Where do you usually get tested for HIV?

**V0009:** Here at Mafakatini.

**PVK:** Ok**.** Do you think HIV is an issues in your community? If so, how big of an issue do you think HIV is for people in your community?

**V0009:** I don’t think it is a big problem because the people I know, who have HIV, are taking treatment**.**  We live together in the community. We don’t have a problem with them.

**PVK:** Do you think the importance of HIV has changed over the past few years, if so why do you think so?

**V0009:** I think it has changed because there is a lot of programmes currently being conducted about HIV and AIDS.

**PVK:** Ok. If HIV was an issues in the community, do you think it would have been more of an issue for some groups than for other groups? For an example, more of an issue for adults, young, men or women?

**V0009:** I think it is more of an issue for people my age, especially young men. From my experience, because I spend most of the time with people my age, and we talk about these things, I think we are more affected.

**PVK:** So it is more of an issue in youth and mostly men?

**V0009:** Yes.

**PVK:** Do you think there is a denial in your community that AIDS is an issue? Are people still in denial that there is AIDS in the community and it is a problem?

**V0009:** I don’t think there are people who are still denying it.

**PVK:** You said you usually get tested in Mafakatini Clinic?

**V0009**: Yes

**PVK:** What is the reputation of Mafakatini clinic as an HIV testing facility you use in your community? Is it a place that people feel comfortable to go to for HIV testing?

**V0009:** Maybe if you are going there for the first time. For me I was scared but I was forced because I had to go for circumcision, it was compulsory that I go for HIV testing first.

**PVK:** What made you scared to go for HIV testing at Mafakatini clinic?

**V0009**: I did not know my status. I was not sure what to expect. I was scared.

**PVK:** But when it comes to the facility itself, is it easy for people to access the clinic? Are people comfortable that their status will be kept confidential?

**V0009**: I have not heard people talking bad things about the clinic.

**PVK:** How are the people who provide HIV counselling like in Mafakatini clinic?

**V0009:** There are good people. They are open.

**PVK:** People in the community are comfortable to go to them for HIV testing?

**V0009**: Yes there are open.

**PVK:** What do you think are the reasons people don’t use their local clinics for HIV testing?

**V0009**: People don’t want to face reality. They are scared to know their status especially young people, because they don’t know they will be treated by people in the community if they find out that they are positive.

**PVK:** Do you think maybe people’s attitudes and beliefs are the greatest barriers to HIV testing? Like you are saying that people are scared because they don’t know how they will be treated by their communities, do you think people’s attitude is a greatest barrier to HIV testing?

**V0009:** Yes I think so. People are scared.

**PVK:** Do you think lack of resources or maybe lack of privacy in the community is also a barrier to HIV testing in your community?

**V0009**: For us, the clinic is closer, we don’t have the issue with the distance but there are people who are lazy to go to the clinic because of the distance. We always get help when we go to the clinic. We have never been chased away because of lack of resources.

**PVK:** Do you have another clinic around this area other than Mafakatini?

**V0009**: This is the only clinic we have.

**PVK:**  So even people from far use this clinic?

**V0009**: Yes they use this clinic. There are people who come as far as Haza and they use the same clinic.

**PVK:** Ok. Have you heard about HIVST before? The one I briefly spoke about at the beginning of the interview?

**V0009**: No.

**PVK:** So you heard it for the first time today?

**V0009**: Even though I heard about something like that but I was not sure and I did not believe that there is such.

**PVK:** What did they say about it?

**V0009**: There were saying that there is going to be a time when people would have to buy something that they can use to test themselves at home. But I did not believe them. As far as I know when you do HIV testing, you have to get counselling first before you know your results.

**PVK:** So you did not believe that someone can just buy something from the pharmacy and do testing without counselling?

**V0009**: I did not believe that is possible.

**PVK:** What do you perceive as a possible challenges to this HIVST, things that might be a barrier when it comes to using this HIVST kit?

**V0009:** I don’t think people would be able to disclose their status if they find out that they are positive. Some people might even commit suicide especially if they get shocked.

**PVK:** What exactly do you think would make people to commit suicide?

**V0009**: They would not know what to do, how to deal with the status.

**PVK:** What if they were tested at the clinic and found out to be positive, do you think they would still commit suicide?

**V0009**: No because they would get counselling. Unlike when you find out when you are alone, nobody would tell you what to do and how to handle the situation. With self-testing you can decided not to disclose your status and when you get sick it becomes difficult to tell people the reason you are sick. But at the same time, there are people who do not want other people to know their status, so for those, they can use this kit. I also think it is a good idea to test yourself.

**PVK:** Ok, you are saying you think it is a good idea to do self-testing, what do you think are the benefits of using this HIVST kit?

**V0009:** Like I am saying if you don’t want to disclose your status, you can do so without feeling embarrassed that other people know that you are positive. If you decided to take treatment, it will only be the people in the clinic that know that you are positive. If you get tested at the clinic, the counsellor might tell their colleagues and the colleagues tell others, in the end you find that all people now know about your illness. If you get treatment at the clinic, you only get to speak to the person who is giving you the treatment, she is the only people who would know about your illness. Sometimes counsellors are from the area, if you know them, you do not feel comfortable because of fear that they might tell other people. For example, I know a lot of counsellors at the clinic, so it becomes uncomfortable to know that someone you know knows your status and you don’t know if that person is going to tell other people about it. I think it is better to do self-testing and you only go to the clinic to get treatment.

**Participant: 0010**

**SQT:** Have you tested for HIV?

**LN:** Yes

**SQT:** If yes, what made you test?

**LN:** The first time I tested they came to school in standard 8 to test us, and after that I’ve been testing to see if it’s still going ok, and I tested again before I got circumcised. The last time I tested was here at the clinic in October.

**SQT:** What made you decide to test today?

**LN**: Today you guys called us and I trust myself and I want to always know my status.

**SQT:** Routine 3 monthly testing?

**LN:** I test maybe 4 times a year, when I was in school they would come test us maybe twice a year.

**SQT:** How big of an issue do you think HIV/AIDS is for people in your community and what makes it an issue of greater or lesser importance?

**LN:** Ay the way I see it within my community it’s not that much of an issue the people seem relaxed, it’s almost like they are not scared of it the way they carry on.

**SQT:** has the importance of HIV/AIDS changed in the community over the past few years and if so why?

**LN:** Ja seems to have changed. It’s as if it’s a style they carry on as if you don’t have it you are a fool.

**SQT:** Is it more of an issue for some groups than for others, and if so, which groups and why?

**LN:** Think mostly for those who drink, they seem careless, the young ones seem to be afraid of it.

**SQT:** Is there denial or do they accept that AIDS is an issue?

**LN:** I think most are in denial they not even taking care of themselves, you find that some have HIV but they continue to drink.

**SQT:** What is the reputation of the HIV testing locations in your community?

**LN:** Ay no this clinic does not have a bad reputation.

**SQT:** Is testing fully confidential?

**LN:** Yes it is confidential.

**SQT:** What are providers like?

**LN:** There’s this one particular guy Sbu Mthalane he does counselling and his a good person, because some would say they tested with Sbu and so on and so forth and he wasn’t the type of guy to disclose your status to others. His a good guy. Other providers are all right they work well.

**SQT:** What do you think are the greatest barriers to HIV testing in your community?

**LN:** Maybe fear and not trusting yourself because you know you have not behaved well sexually, or after drinking and sleeping with a girl you met while drinking and then being scared to test because of the fear that you might already have HIV.

**SQT:** What structural factors (e.g. availability of services, lack of resources, lack of privacy in the community, etc.)?

**LN:** Structures are fine, the issue is the lines at the clinic are separated so if you stand in a particular line then everyone knows what you came to do. For instance my sister had Tb and while at the clinic saw one of our neighbours, and she came home and told me that whilst at the clinic she saw this guy and for sure his going to talk and say she has HIV, and at times you can find that maybe I’ve been sent by my mom to fetch her pills but once I’m seen in that line people will talk and say I’ve got HIV.

**SQT:** What do you know about HIVST?

**LN:** Maybe the person is scared to be tested at the clinic and may want to know their status first before I go to the clinic.

**SQT:** Where did you hear about HIVST?

**LN:** There’s an aunt of mine who works at the clinic, and my cousin was scared to go test so he asked her to bring him the kit so he could test at home.

**SQT:** What do you perceive as possible challenges to HIVST?

**LN:** Counselling because there would be nobody there to advise you, and you have major stress, its better when you test and have someone to advise you should you get results that upset you.

**SQT:** What do you think would be the benefits of HIVST?

**LN:** I don’t see where the benefit there is because when you find out that you have HIV then you will hide it and that can eat you up inside until you eventually die.

**Participant: 0011**

**SQT:** Have you tested for HIV?

**SK:** Yes

**SQT:** If yes, what made you test?

**SK:** I wanted to know where I stood in regards to my HIV status

**SQT:** What made you decide to test today?

**SK:** I wanted to check again if everything is still going good

**SQT:** Routine 3 monthly testing?

**SK:** I test twice a year

**SQT:** How big of an issue do you think HIV/AIDS is for people in your community and what makes it an issue of greater or lesser importance?

**SK:** It’s a big issue due to carelessness and lack of education with the people

**SQT**: Has the importance of HIV/AIDS changed in the community over the past few years and if so why?

**SK:** Ja they recognise that it’s an issue, they are aware of aids within the community

**SQT:** Is it more of an issue for some groups than for others, and if so, which groups and why?

**SK:** I would say the ones that are in puberty around the age of 18 to 20, it’s the way they carry themselves they don’t show that they fear HIV they are careless, you see them Friday evenings the way they behave.

**SQT:** Is there denial or do they accept that AIDS is an issue?

**SK:** Ja they accept it as an issue

**SQT:** What is the reputation of the HIV testing locations in your community?

**SK:** We don’t have many locations it’s just the clinic, they treat us well and they do their jobs

**SQT:** Is testing fully confidential?

**SK:** Yes it is confidential

**SQT:** What are providers like?

**SK:** Well the providers I can’t lie, they sometimes don’t want to work, because you find that the clincs at times are full but you find them sitting doing nothing.

**SQT:** What do you think are the greatest barriers to HIV testing in your community?

**SK:** I wouldn’t know I won’t lie I don’t know why.

**SQT:** What structural factors (e.g. availability of services, lack of resources, lack of privacy in the community, etc.)?

**SK:** The services sometimes are not good, you find at times that they do not have medicine

**SQT:** What do you know about HIVST?

**SK:** Well I do know that you have to know your status

**SQT:** Where did you hear about HIVST?

**SK:** The way I see it will help so that you do not need to go to the clinic, I heard about HIVST for the first time here at Caprisa

**SQT:** What do you perceive as possible challenges to HIVST?

**SK:** When a person is alone at home with the kit they may get scared, secondly if they find out they have HIV they may have a problem

**SQT:** What do you think would be the benefits of HIVST?

**SK:** It saves time you will not need to travel to a particular location to get tested

**Participant: 0012**

**SQT:** Have you tested for HIV?

**KM:** Yes

**SQT:** I what made you test?

**KM:** I wanted get circumcised

**SQT:** What made you decide to test today?

**KM:** I trust myself I have no problem, even if I find that I am positive u see, I wouldn’t have an issue I will go to counselling get pills and carry on.

**SQT:** When you say you trust yourself what does that mean?

**KM:** I trust that I have nothing, I’m not sick.

**SQT:** How big of an issue do you think HIV/AIDS is for people in your community and what makes it an issue of greater or lesser importance?

**KM:** It’s an issue because let’s just say I get sick and I need to go get treatment, then the people will say I thought I was clever, now I’m fetching pills and I’m sick. That is why it is an issue for people.

**SQT:** Has the importance of HIV/AIDS changed in the community over the past few years and if so why?

**KM:** The way I see it now, the people I live with they say if you don’t have HIV then you are not in style. So it is something they do not care about.

**SQT:** Is it more of an issue for some groups than for others, and if so, which groups and why?

**KM:** It is the teenagers, because I will tell you about where I come from as boys we play soccer ,so if a guy has a new girlfriend they will say you have such a beautiful girl ,so they then influence you to sleep with her without a condom

**SQT:** I hear you, can you help me understand the reason why they would tell him to sleep with her without a condom?

**KM:** It starts from the beauty, if she’s beautiful then they think she doesn’t have HIV.

**SQT**: Is there denial or do they accept that AIDS is an issue?

**KM:** Ja it’s an accepted issue

**SQT:** What is the reputation of the HIV testing locations in your community?

**KM:** They are all right, for instance, there’s the males that work here with the mobile trailer and we play soccer with they check me for HIV and I’ve never heard that they speak about what I do. Then its Sbu from the clinic him also he never talks about our test results.

**SQT:** Is testing fully confidential?

**KM**: I wouldn’t be sure, but I trust them that it stays with them

**SQT:** What are providers like?

**KM:** You see I don’t come to the clinic often, but when I hear family members at home talking, for instance my aunt comes to the clinic to get her TB medication, so there is a nurse lady that treated her badly to a point where she said she will wait for this lady at the bus stop and beat her up, she really treated her badly.

**SQT**: It was just this one particular nurse that treated her badly not the stuff as a whole?

**KM:** yes

**SQT**: What do you think are the greatest barriers to HIV testing in your community?

**KM:** They fear what others will say if u are now sick and you thought you were clever

**SQT**: Even though it’s not known what the result will be, just because you are seen going to test they will think something must be wrong for you to do that?

**KM**: Ja exactly like if you swap girls around, they joke around saying that one must have HIV. So when your guys say that you will put up a front like you don’t care, but surely when you are alone you think I did such and such?

**SQT:** What structural factors (e.g. availability of services, lack of resources, lack of privacy in the community, etc.)?

**KM:** No everything is good, its peoples fear and knowing how they have been risky sexually. And it’s known that if you at a particular line they know that you are here for HIV pills, it was better when the line was behind where it is hidden.

**SQT:** What do you know about HIVST?

**KM:** No I know nothing because my soccer coach is a HIV counsellor he works at new start but he tells us to test at the clinic. It’s not allowed for him to give you a kit because if you find out that you are sick you may just shoot yourself.

**SQT:** Where did you hear about HIVST?

**KM:** Here with you

**SQT:** What do you perceive as possible challenges to HIVST?

**KM:** People could kill themselves, like for me earlier I said I trust myself but when you showed me the kit, I realised it could have been a different story.

**SQT:** What do you think would be the benefits of HIVST?

**KM**: For me if it comes out that I am sick, I would like for my family to know so they can take care of me, if I need to take pills to encourage me.

**Participant: 0013**

**PVK:** Have you tested for HIV before?

**V0013**: Yes

**PVK:** What made you get test for HIV?

**V0013**: I wanted to know my health status.

**PVK:**  Okay, so today you are going to get tested, right?

**V0013**: Yes I agree to get tested

**PVK:** What makes you agree to get tested?

**V0013**: It is because I want to know if my status is still the same.

**PVK:** How often do you usually get tested for HIV?

**V0013:**  I don’t get tested often, it has been couple of times but now I am trying to make sure I get tested at least once in three months.

**PVK:** Ok**.** How big of an issue do you think HIV is for people in your community?

**V0013:** It is a big issue because lots of us are affected by it but we are scared to get tested. But it has affected a lot of people but they rather keep to themselves instead of seeking help.

**PVK:** Do you think the importance of HIV has changed over the past few years? Has it changed for better or people still don’t see it as an important issue? If so, why do you think so?

**V0013:** For me I think it has not changed much people are still not keen to get tested. They don’t even want to come to CAPRISA to get tested. They don’t take it as something serious.

**PVK:** Ok. But do you think if HIV was an issues in the community, it would have been more of an issue for some groups than for other groups, like the youth, men or women?

**V0013:** It is of an issue for the youth, especially young men. They don’t want to get tested because they are scared that they will be laughed at if they tested positive.

**PVK:** So it is more of an issue in youth and mostly men?

**V0013:** Yes.

**PVK:** Do you think there is a denial in your community that AIDS is an issue?

**V0013:** Yes majority of people are still in denial. The reason they are on denial is that they have not been tested yet that is why they are on denial. They don’t know where they stand in terms of their HIV status.

**PVK:** Where do you get tested for HIV?

**V0013**: Here at Mafakatini Clinic?

**PVK:** Ok, what is the reputation of Mafakatini clinic as an HIV testing facility used by the people in your community? Are people comfortable using the clinic for HIV testing?

**V0013:** It is scary to get tested for HIV because it is known as the diseases that kills but once you get tested, you are relieved.

**PVK:** But do you think people are comfortable coming to Mafakatini clinic to get tested for HIV testing?

**V0013**: People don’t have a problem coming to the clinic because they know they get counselling. They are counselled and encouraged on what to do, how to live their lives when they get infected with HIV.

**PVK:** But when it comes to the facility itself, is it easy for people to access the clinic? Are people comfortable that their status will be kept confidential?

**V0013**: I have not heard bad things about the clinic. People are comfortable to come to this clinic.

**PVK:** What do you think are the reasons people don’t use their local clinics for HIV testing?

**V0013**: I would say it is the fear. It is just the fear.

**PVK:** Do you think people’s attitudes and beliefs are the greatest barriers to HIV testing? Like if you go for HIV testing, people are going to think otherwise about you, they start treating you differently, like they believe that maybe you have AIDS, something like that?

**V0013:** I don’t think so because when you go to the clinic you get counselling. Even friends can support you and make you feel comfortable. I don’t think that stops people from doing HIV testing.

**PVK:** Is it easy for people in your community to access this clinic?

**V0013**: Yes

**PVK:** Are resources courses for HIV testing always available when you go to the clinic for HIV testing?

**V0013**: Resources are always available. There is no time when people get to the clinic and don’t they are told to come back some other time.

**PVK:** Ok. Have you heard about HIVST before? The kit I told you about before we started this interview?

**V0013**: No. I am hearing about this for the first time.

**PVK:** So as I have explained to you how you can access it and how it works, what do you perceive as a possible challenges to this HIVST kit?

**V0013:** I think it is something that has to be approved because the reason people don’t come to clinics for HIV testing is the fear that people will see them coming out of the room crying, other people might suspect that they are positive, I think it is better that people can access it at pharmacy so that people can test themselves then decide whether they want to talk to their family members on what to do.

**PVK:** Ok, what do you think are other benefits of using this HIVST kit other than that people can decide to test themselves?

**V0013:** Like I said, I think people would be more comfortable to test themselves other than being tested at the clinics.

**Participant: 0014**

**PVK:** Have you tested for HIV before?

**V0014**: Yes

**PVK:** What made you get test for HIV?

**V0014**: Because I had to go for circumcision.

**PVK:** Okay, so today I asked you to get tested, right?

**V0014**: Yes

**PVK:** What makes you agree to get tested today?

**V0014**: I want to know if my status is negative or positive.

**PVK:** You said you only tested because you were going for male circumcision, other than that have you ever tested before?

**V0014:**  No.

**PVK:** Ok**.** How big of an issue do you think HIV and AIDS is for people in your community?

**V0014:** It is a big issue because there are lots of children who have grown up without parents. There are parents who are taking treatment together with their children. The mother needs someone to take care of her while she is also taking care of her sick child. It is a big problem.

**PVK: D**o you think the importance of HIV has changed over the past few years? Is it something that is a priority in the community or people don’t think it is that important?

**V0014:** Yes. I think it is something that needs to be dealt with. They need to find ways to prevent it from killing lots of people. Maybe they will find a better cure than what we currently have.

**PVK:** Ok. Do you think HIV is more of an issue for some groups than for other groups, if so, which groups and why?

**V0014:** I think it depends of individuals and their behaviour. Some people do stupid things when they are drunk and they regret later after they have messed up their lives, after they have gotten infected with HIV.

**PVK:** So that often happen to which group?

**V0014:** Usually it is teenagers, the youth. It mostly affects the youth.

**PVK:** Do you think there is a denial in your community that AIDS is an issue or people have accepted that AIDS is an issue in the community?

**V0014:** I live with someone who is HIV positive. He is my uncle. He is 60 years this year. When he told me the story, he said he dated 2 women who were HIV positive in 1998 he slept with both of them without a condom, I don’t know if condoms were available at that time but he said they were not using condoms during those days but he never got infected. He said since then he had slept with 7 different women who were HIV positive, they never used condoms, I but he never got infected. All the women he slept with have since passed away due to AIDS but he is still alive and negative.

**PVK:** Does he go for HIV testing regularly?

**V0014**: Yes he test regularly.

**PVK:** Do you think maybe he is one for the people that are in denial that AIDS is an issue?

**V0014**: I think so but at the same time I am happy that he goes for HIV testing regularly. He tells us that he will never get AIDS.

**PVK:** Does he believe that AIDS exists?

**V0014**: He believes that AIDS exist because he knows that all women he slept with died of AIDS, all 7 of them but he never got infected.

**PVK:** He is one of the few lucky people.

**V0014**: Yes but he used to tell us that he was strengthened by an old man who was a herbalist from Mhlabuyalingana area, who gave him medicine to take that would strengthen him and he told him that he will never contract any sexually transmitted disease in his life. He said after taking that medicine he never contracted any STIs. He said when he later met these girlfriends that were HIV positive, he was already strengthened by this herbalist. He said he visited the herbalist when he contracted cauliflower, the herbalist gave him the strengthening medicine that he used and he told him that he will never contract any disease in his life.

**PVK:** So he doesn’t use condoms during sex at all?

**V0014**: He does use condoms. It is not something that he is proud of, that he slept with 7 women with HIV that is why he said he is using condoms consistently now. But he acknowledges that he never used condoms with the 7 women who died of AIDS.

**PVK:** Ok. How many times or how often do you get tested?

**V0014**: I have tested once

**PVK:** Where did you get tested for HIV?

**V0014**: I tested in Cedara. We were recruited from school by Maykhethele Project and we went for camping for 3 days where we were trained about manhood before we went for circumcision. I was circumcised by the doctor, I can’t remember his name because there were many doctors who were there, but he was one of the doctors who were there.

**PVK:** So if it was not for circumcision, you would not have been tested for HIV yet?

**V0014**: I was going to get tested eventually. The thing is sometimes we get lazy. Also currently I don’t have an ID so it becomes difficult for me to do things. At least at the camp they only wanted birth certificate; that is why I was able to go there.

**PVK:** How come you don’t have an ID?

**V0014**: I lost my birth certificate, it was misplaced and I only got it recently in 2015. I am going to take my ID beginning of next year.

**PVK:** Ok, according to your knowledge, what is the reputation of HIV testing facilities used by the people in your community? Or where do most people in your community get tested for HIV?

**V0014:** I think most people get tested in Mafakatini clinic and sometimes they use mobile clinic.

**PVK:** But do you think testing is fully confidential to these facilities when people are going there for HIV testing?

**V0014**: It is confidential. It is kept between you and the person who conducts the test. I have never heard anyone talking about results of someone who got tested, because even in the community, you don’t hear people fighting about that.

**PVK:** What do you think are the greatest barriers to HIV testing in your community?

**V0014**: Sometimes people are scared because of the things they had done in the past. You think about people you have had sex with and you get scared. People are scared. I have another uncle whom I live with, he is positive and he knows but he doesn’t want to get tested. The family had to force him to go to the clinic to get the medicine, now he is taking treatment and he is recovering well because he had lost a lot of weight. Now he acknowledges that he nearly lost his life and he thank us for helping him. People are scared to be seen by other people standing in a queue for treatment. What I always tell people, even my friends is that I would be happy to find out that I am positive before I get sick because once you get sick, it takes time to recover.

**PVK:** Is it easy for people in your community to access these HIV testing facilities?

**V0014**: Yes it is easy. It is close by

**PVK:** Are resources for HIV testing always available in these facilities?

**V0014**: Resources are always available. People don’t have to be returned back home or told to wait.

**PVK:** Are people’s attitudes and beliefs the greatest barriers to HIV testing?

**V0014:** Like I said, people don’t want to be seen standing on queues. Sometimes they don’t even take treatment because they are thinking about what other people would think of them.

**PVK:** Ok. Have you heard about HIVST before?

**V0014**: I first heard about it from Thabane (one of the participant recruited). He told us that you are looking for people to talk to about it, he explained to us what it is all about.

**PVK:** Other than that, you have never heard about it before?

**V0014:** No.

**PVK:** What do you perceive as possible challenges to this HIVST?

**V0014**: I think people need to be trained properly about it before they can use it. For me I would prefer to go to the clinic because at least there is always someone who would assist you. Someone who is going to ask you questions, like what would you do if you tested positive. She will give you advise on what to do and what to do when you are tested positive and when to start taking treatment. If you test yourself, alone at home, there are so many things and thoughts that you might come to your mind, like you might even do things that you have not planned to do, things like committing suicide. I think it is dangerous to test yourself without a counsellor. You need someone to talk you. You need a counsellor.

**PVK:** Ok, what do you think are benefits of using this HIVST kit?

**V0014:** There are benefits. There are lots of benefits especially for those that are scared to go to the clinics. At least they can have this and test themselves at home.

**Participant: 0015**

**SQT:** Have you tested for HIV?

**MNZ:** Yes I have

**SQT**: If yes, what made you test?

**MNZ:** I got into a bicycle accident when I was younger with my cousin we were hurt, I tried helping him and touched his blood, so I had to get a test done.

**SQT:** What made you decide to test today?

**MNZ:** It’s that I know that they say in the end you have to know your status, so today I wanted to know.

**SQT**: Routine 3 monthly testing?

**MNZ**: I don’t test often, I last tested last year.

**SQT:** How big of an issue do you think HIV/AIDS is for people in your community and what makes it an issue of greater or lesser importance?

**MNZ:** what can I say, I notice that as the youth we don’t carry ourselves well.

**SQT:** Has the importance of HIV/AIDS changed in the community over the past few years and if so why?

**MNZ:** Ay some people don’t see it as that important since the pills are also readily available**.**

**SQT**: Is it more of an issue for some groups than for others, and if so, which groups and why?

**MNZ:** Ay maybe the women, the young ones from 19 to 30. It’s the way they do things some drink alcohol and lose control and end up having unprotected sex.

**SQT:** Is there denial or do they accept that AIDS is an issue?

**MNZ:** Yes it’s accepted

**SQT:** What is the reputation of the HIV testing locations in your community?

**MNZ:** Some there’s this thing of if you have it then you must start with the pills and you find that a person is scared to go fetch the pills at the clinic.

**SQT:** What could cause a person to be uncomfortable to fetch their treatment at the clinic**?**

**MNZ:** It’s because people talk so that makes the people sacred to go and get treatment**.**

**SQT:** Is testing fully confidential?

**MNZ**: I would not know for sure

**SQT:** What are providers like?

**MNZ**: They are all right

**SQT:** What do you think are the greatest barriers to HIV testing in your community?

**MNZ**: Its mostly fear of knowing

**SQT:** Are people’s attitudes and beliefs about HIV barriers?

**MNZ:** Yes people get influenced by other people’s opinions.

**SQT:** What structural factors (e.g. availability of services, lack of resources, lack of privacy in the community, etc.)?

**MNZ:** ay No

**SQT**: What do you know about HIVST?

**MNZ:** I did not know anything

**SQT:** Where did you hear about HIVST?

**MNZ**: It was my first time hearing about it here

**SQT:** What do you perceive as possible challenges to HIVST?

**MNZ:** When I self-test the result is my secret

**SQT:** What do you think would be the benefits of HIVST?

**MNZ:** It’s the privacy and only you knowing your status.

**Participant: 0016**

**SQT:** Have you tested for HIV?

**BPS:** Yes

**SQT:** what made you test?

**BPS:** My first test was with Caprisa because in order for me to join the study I had to do a HIV test.

**SQT:** What made you decide to test today?

**BPS:** Today it’s because it has been a while since I last tested and also in life you have to know how your health is.

**SQT**: what has been your testing routine ?

**BPS:** When I was still in the gel study I would test every month

**SQT**: How big of an issue do you think HIV/AIDS is for people in your community and what makes it an issue of greater or lesser importance?

**BPS:** I see it as a big issue and attitudes are such that they will say to those who are healthy and have not checked their status things like, just like the rest of us you too have the virus it’s just that you do not know as yet

**SQT:** Has the importance of HIV/AIDS changed in the community over the past few years and if so why?

**BPS:** I see acceptance with a lot of people, and it is important to know where it is that you stand in regards to your HIV status.

**SQT**: Is it more of an issue for some groups than for others, and if so, which groups and why?

**BPS:** Ja it is a problem for certain people, for those who go test and results are inconclusive. I think the issue is for older people those 40 and above.

**SQT:** Where did you hear about HIVST?

**BPS:** First time hearing it from you today.

**SQT:** What do you perceive as possible challenges to HIVST?

**BPS:** It’s not right, what if I test and last I knew I was negative, then the kit says I’m positive, so the decisions that I would make are many and also unknown.

**SQT:** What do you think would be the benefits of HIVST

**BPS**: Benefits are you can test at any time that you feel like.

**Participant: 0017**

**PVK:** Have you tested for HIV before?

**V0017**: Yes

**PVK:** What made you get tested for HIV at that time?

**V0017**: At that time the reason I got tested is because I was pregnant.

**PVK:**  Okay, how often do you get tested for HIV or how many times have you been tested for HIV, if you can remember?

**V0017**: Maybe about 5 times or so. Usually when you go to the clinic for HIV testing, they encourage you to come back at least every after 3 months. But it doesn’t happen like that, I usually get tested if there is something funny that I notice in my body or if I feel sick then I rush to the clinic for testing, Like last year October, I had this unidentified disease, I did not know what was happening in my body, it was really bad. I decided to get tested for HIV. At the clinic they told me to wait for another 3 months and come back to repeat HIV test. I was very very sick.

**PVK:** Were you not scared to get tested when you were sick?

**V0017**: Yes I was scared. In fact even if I was tested positive at that time, I would not have been surprised. I was expecting any results. Luckily my results were negative but I have been anxiously waiting for January to get tested again because I last tested in October. That is why I decided to come today

**PVK:**  Are you happy to get tested today?

**V0017**: Yes I am happy because I was advised to get tested after 3 months. Since I last tested in October, I am due for another test and I am happy to do it.

**PVK:** How big of an issue do you think HIV and AIDS is for people in your community?

**V0017:** It is a big issue in the area where I come from. In fact it is so big that people have now started talking openly about it.

**PVK:** The fact that people are talking about it openly, does it make it a big issue.

**V0017**: It is a big issue and it is real. There are so many people who are infected and they are not afraid to talk about. The way they openly talk about it, they even make one feel comfortable that even if you get infected, you know there are people who are living with HIV like you and they are living healthy lives and you see them in your community every day.

**PVK:** Do you think the importance of HIV has changed over the past few years?

**V0017:** Yes it has changed. A while ago, people who were infected used to get sick, they would not tell anyone, they would be sick until they die. Nowadays, people talk openly about it, they talk random people about being HIV positive and about taking treatment. This has made me realised that this is a disease that I can handle. Even if I get positive results, I would handle it because it is something that I am not longer afraid of. People no longer collapse if they find out that they are positive.

**PVK:** Ok. Do you think HIV is more of an issue for certain groups than for other groups, like youth, adults, men or women? If this is the case, which groups are more affected than others and why do you think so?

**V0017:** In my own opinion and from what I have learned through talking to different people, HIV affects all of us but women are more affected because they are not afraid to get tested. Men don’t like to get tested, this becomes an issue for women because they are the ones who always have to push that the couple get tested. Women are not afraid to get tested, men are very difficult even if they are in their death bed, they still don’t want to accept and they refuse to get tested.

**PVK:** So as you have mentioned that nowadays people are not scared to talk about HIV, do you think there is still a denial in some of your community members that AIDS is an issue?

**V0017:** People have accepted that AIDS exists.

**PVK:** Where did you often get tested for HIV?

**V0017**: I have tested in Mpophomeni clinic as well as Howick clinic.

**PVK:** Ok, what is the reputation of these HIV testing facilities used by the people in your community in terms of how people are treated as well as in terms of confidentiality?

**V0017:** The discussion is always between you and the counsellor. Nobody get to know about your results.

**PVK:** What are service providers like at these facilities? Are people free to go to them without any fear?

**V0017**: People there are really nice. They first introduce themselves, ask how you feel and if you are ready to get tested. They ask you all questions that make you feel comfortable even if you were scared.

**PVK:** What do you think are the greatest barriers to HIV testing in your community?

**V0017**: I think those people who maybe have not talk to the kind of people I have come across. They meet people who are negative, you say bad things about HIV, and such talks would scare you from getting tested because people are already saying horrible things about being HIV positive. I have met women who openly told me, “look at me, what is the difference between me and you. I am positive, I am taking treatment. If you look at the person, she is fine and she looks even better than you. That also gives you hope. If I think about how sick I was last year, I was praying to get positive results so that I can take the treatment because I wanted to get better so that I can take care of my children. I have seen how fresh people who are taking ARVs are, unfortunately when I get tested, my results came back negative.

**PVK:** So basically it depends on what you believe in and that determines whether you can have HIV testing or not?

**V0017**: Yes

**PVK:** Is it easy for people in your community to access these HIV testing facilities?

**V0017**: For Mpophomeni clinic, it is easy for us to access it even though it is a bit of a distance

**PVK:**  Are resources for HIV testing always available in these facilities?

**V0017**: There are instances where you get to the clinic and find that they don’t have medication. That is very common. Sometimes even if your child is sick and you take your child to the clinic, they tell you that they don’t have medication. It happens quite often.

**PVK:** Ok, but does it happen specifically for HIV testing? Like for example, you get to the clinic and they tell you they don’t have kits to perform HIV test or the counsellor is not available to do HIV testing?

**V0017:** No**,** I never had that experience. Whenever I go to the clinic the counsellor is always there.

**PVK:** Ok. Have you heard about HIVST before?

**V0017**: No.

**PVK:** So it was your first time to hear about it today?

**V0017**: Yes it is the first time.

**PVK:** So as I have explained briefly about it earlier, what do you perceive as possible challenges to this HIVST? I know you have noted used it but what do you foresee as challenges with this method?

**V0017**: Because people are different. I think for people who are still struggling to understand, I foresee some problem because even if they buy it and have it at home, then the person get positive results, maybe it would be difficult for that person to accept the results and the person might take stupid decision because they don’t understand HIV or not ready to accept that people live with HIV and people get treatment and I live longer lives. I think people who are not ready are going to be a problem.

**PVK:** Ok, what do you think are benefits of using this HIVST kit, if there are any benefits?

**V0017:** It is a good thing because you get an opportunity to test yourself. Yes the counsellor can do the same thing to you but the counsellor is in control of the kit but because you do everything yourself, you open the kit, you prick your finger yourself, you read results yourself, for that reason you are likely to believe the results and you are sure of the results.

**Participant: 0018**

**SQT**: Have you tested for HIV?

**QPM: Y**es I have

**SQT**: If yes, what made you test?

**QPM:** I wanted to know my status.

**SQT**: What made you decide to test today?

**QPM:** I haven’t tested in a long time, I would like to know my status.

**SQT**: What is your testing HIV Routine?

**QPM:** I last tested in 2012 when I was pregnant.

**SQT**: How big of an issue do you think HIV/AIDS is for people in your community and what makes it an issue of greater or lesser importance?

**QPM:** Most people don’t see it as an issue, and it seems not to affect them when they have not yet tested for HIV, and some are scared to test, and might even say why did I test? Now I’ve hurt myself emotionally.

**SQT**: Is it more of an issue for some groups than for others, and if so, which groups and why?

**QPM:** I see the problem for older people, they get HIV whilst when a person is old it’s a problem, because if you do not test as time goes on it eats away at you. Some old people they get into new relationships not knowing that the new person they are with is already on HIV treatment. So it’s not easy for the older people to go test because people do not expect people of a certain age to still be jolling.

**SQT**: When you say old people which age groups are you referring to?

**QPM:** Maybe 50 or 60

**SQT**: Is there denial or do they accept that AIDS is an issue?

**QPM:** Yes there is acceptance because people see people around them getting sick.

**SQT**: What is the reputation of the HIV testing locations in your community?

**QPM:** Well the issue is being tested by people who are your neighbours.

**SQT**: Is testing fully confidential?

**QPM:** I don’t think it’s confidential, the person could be unable to keep a secret**.**

**SQT:** What are providers like?

**QPM:** Well they treat us in a way that well doesn’t mean much.

**SQT**: What do you think are the greatest barriers to HIV testing in your community?

**QPM:** Some might fear the nurse exposing their status and others its just the fear of maybe having a heart attack if the results are not good maybe it was better if they didn’t know ,now all they can do is think and think all the time.

**SQT**: What structural factors (e.g. availability of services, lack of resources, lack of privacy in the community, etc.)?

**QPM:** There is especially in the community hall we use, it’s not a good space to conduct HIV testing.

**SQT**: What do you know about HIVST?

**QPM:** I won’t lie I know nothing.

**SQT**: Where did you hear about HIVST?

**QPM:** I first heard it from you.

**SQT**: What do you perceive as possible challenges to HIVST?

**QPM**: You could do the test wrong, maybe get results which are not true due to the fact that you cannot do the test.

**SQT:** What do you think would be the benefits of HIVST?

**QPM:** I see none, I mean what if I test and it’s positive now I’ve caused myself stress and my result could be anything especially since I last tested in 2012.

**Participant: 0019**

**SQT:** Have you tested for HIV?

**NFK:** Yes

**SQT:** What made you test?

**NFK:** My starting with Caprisa studies was because they said you get money it was the gel study, I came because of the money but from there I realized that testing is important.

**SQT:** What made you decide to test today?

**NFK:** I’m now used to testing and I’m not at ease if 3 months go by without me testing

**SQT:** Routine 3 monthly testing?

**NFK:** I test every 3 month, but should I get sick in between I then test as well.

**SQT**: How big of an issue do you think HIV/AIDS is for people in your community and what makes it an issue of greater or lesser importance?

**NFK:** Where I come from HIV is like fashion, if you don’t have it they say go test you also probably have it**.** Stigma

**SQT**: Has the importance of HIV/AIDS changed in the community over the past few years and if so why?

**NFK:** I think they see it as a less important issue.

**SQT**: Is it more of an issue for some groups than for others, and if so, which groups and why?

**NFK**: It’s more of an issue in our age group from 18 to 35. Those are the people that don’t have a problem with telling you so what if I have it. The young ones still at school, they wish to be protected from it.

**SQT**: Is there denial or do they accept that AIDS is an issue?

**NFK:** Yes they accept and see it

**SQT**: What is the reputation of the HIV testing locations in your community?

**NFK:** We only have one clinic to test, people fear each other the stuff and nurses are fine. If someone sees you going to check your status, if they see someone else there to test then they turn away ,or if you go get your HIV treatment and see someone else there for the same purpose then they go spreading the word about each other of how they saw so and so at the clinic for treatment

**SQT**: Is testing fully confidential?

**NFK:** I would think so

**SQT:** What are providers like?

**NFK:** Our clinic is not 100% good but last time I went there to test the nurse asked me when last I had sex with my boyfriend, I told her I’m abstaining at the moment, she then asked me why did I come to test. I can say that she failed to motivate me that testing is important. The stuff is unlike the Caprisa stuff they don’t show that much care.

**SQT**: What do you think are the greatest barriers to HIV testing in your community?

**NFK:** I think its people being fearful of each other. They fear others knowing their status**.**

**SQT**: Are people’s attitudes and beliefs about HIV barriers?

**NFK:** It could be because you find that 2 women shares a guy then one of the women tests and is positive, then word gets to the second women she will not even bother testing because of the fear that she too is positive and being scared to the face the truth.

**SQT**: What structural factors (e.g. availability of services, lack of resources, lack of privacy in the community, etc.)?

**NFK:** At our clinic you wait for a long time. If the line goes all the way outside and it’s raining you get wet.

**SQT:** What do you know about HIVST?

**NFK:** Nothing

**SQT:** Where did you hear about HIVST?

**NFK:** I heard from you when you called. I even told them at home that this is something else.

**SQT:** What do you perceive as possible challenges to HIVST?

**NFK:** There’s no challenges, but I think after a while people will get lazy, some could use it to test children**.**

**SQT**: What do you think would be the benefits of HIVST?

**NFK:** It would benefit me to have it at a clinic near me, like I told you this is not the clinic I use its too far. So if the clinic near me has it that would make things so much easier

**Participant: 0020**

**SQT:** Have you tested for HIV?

**NFK:** Yes I have.

**SQT:** If yes, what made you test?

**NFK:** I wanted to know where I stand. It’s because I always like to know what’s happening with my life

**SQT:** What made you decide to test today?

**NFK:** I wanted to know where I stand.

**SQT**: Routine 3 monthly testing?

**NFK:** I test every 3 months

**SQT**: How big of an issue do you think HIV/AIDS is for people in your community and what makes it an issue of greater or lesser importance?

**NFK:** I notice it’s an issue mostly for men, men don’t like to test they say their girlfriends must test in order for them to know their own status, which is wrong because it could be that the lady is negative while the man could be positive.

**SQT**: Has the importance of HIV/AIDS changed in the community over the past few years and if so why?

**NFK:** No I don’t think it has changed, attitudes are still the same**.**

**SQT**: Is it more of an issue for some groups than for others, and if so, which groups and why?

**NFK:** I see it as the 30 something men that still would like to get married.

**SQT:** Is there denial or do they accept that AIDS is an issue?

**NFK:** The way I see it there is still denial, they know full well that aids is out there but they still don’t protect themselves, they cheat, I notice that on both sides with males and females. They don’t protect themselves they drink and sleep around.

**SQT**: What is the reputation of the HIV testing locations in your community?

**NFK:** At our local hospital, there’s stigma, you will see with certain colour footprints on the floor mean that you are going to a particular place and people know what the different colours mean, and some will fear to go there and get their treatment.

**SQT**: Is testing fully confidential.

**NFK:** I would say so the way I see it**.**

**SQT**: What are providers like?

**NFK:** I see them as being ok.

**SQT**: What do you think are the greatest barriers to HIV testing in your community?

**NFK:** The way I see it, a barrier could be the fact that now you have to take medication for the rest of my life, rather I not know**.**

**SQT**: What structural factors (e.g. availability of services, lack of resources, lack of privacy in the community, etc.)?

**NFK:** No the structures are fine.

**SQT:** What do you know about HIVST?

**NFK:** I heard it on the radio in the morning, people had different views some said its good some said it’s not. The way I see it, it’s not ok when done without counselling.

**SQT:** Where did you hear about HIVST?

**NFK:** On the radio

**SQT**: What do you perceive as possible challenges to HIVST?

**NFK:** A person could find out they are positive and sleep with many people to spread it.

**SQT**: What do you think would be the benefits of HIVST?

**NFK:** The benefit is that only you will know your status.

**Participant: 0021**

**SQT:** Have you tested for HIV?

**NC**: Yes.

**SQT:** If yes, what made you test?

**NC:** I tested because I was going to start a study. Because I always want to know where I stand with my status.

**SQT:** What made you decide to test today?

**NC**: Because I always want to know where I stand with my status.

**SQT:** Routine 3 monthly testing?

**NC**: I test maybe twice a year

**SQT:** How big of an issue do you think HIV/AIDS is for people in your community and what makes it an issue of greater or lesser importance?

**NC**: Most of them don’t care about the issue, they think well it doesn’t really matter whether I test or not because life goes on, majority just don’t care. - Not an issue

**SQT:** has the importance of HIV/AIDS changed in the community over the past few years and if so why?

**NC:** Compared to before with HIV you would be isolated, now if a person is infected they are more willing to come out

**SQT:** Is it more of an issue for some groups than for others, and if so, which groups and why?

**NC:** Mostly, it’s the youth ages 16 to 35, most of the time they are careless there’s blessers, and do drugs and alcohol there you cannot remember that you have to protect yourself,

**SQT:** Is there denial or do they accept that AIDS is an issue

**NC:** There’s denial.

**SQT:** What is the reputation of the HIV testing locations in your community?

**NC:** The nurses there are rude, they will force you to test when with some things as a patient you have to want to do them, and not be forced, so they will say if you do not do the test then that means that you will be helped.

**SQT:** Is testing fully confidential?

**NC:** Ay no, the place we test at there’s no privacy and they don’t give you proper counselling, and if you test positive they do not tell of what to do next, most people who have been there complain about that.

**SQT**: What are providers like?

**NC:** I would say they are not right, they make you not want to go to the clinic they make you feel like oh if I go there I will be treated poorly, as an ill person that wouldn’t make me feel right.

**SQT:** What do you think are the greatest barriers to HIV testing in your community?

**NC:** Ok its thinking that even if I test what difference does it make because this is like all the other diseases that are out there .If I test what difference does it make.

**SQT:** What structural factors (e.g. availability of services, lack of resources, lack of privacy in the community, etc.)?

**NC**: I don’t think so

**SQT:** What do you know about HIVST?

**NC:** I did not know anything

**SQT:** Where did you hear about HIVST?

**NC:** Here from you.

**SQT:** What do you perceive as possible challenges to HIVST?

**NC**: Sometimes we live in homes that do not have privacy, and there’s nobody there to counsel you. I think testing at home is off.

**SQT:** What do you think would be the benefits of HIVST?

**NC:** To test at home the result is your knowledge, and it’s you who decides who you want to tell

**Participant: 0022**

**SQT**:Have you tested for HIV?

**KN**: Yes I have

**SQT:** If yes, what made you test?

**KN**: I was sick, and when I would urinate it would be painful, and I knew that with my boyfriend at the time I wasn’t his only girlfriend. – Mandatory testing, Risky sexual practice

**SQT:** What made you decide to test today?

**KN**: Because I want to know where I stand – HIV status awareness

**SQT:** Routine 3 monthly testing?

**KN**: Truth be told I have never left home randomly and said I will go test at the clinic ,I test because I have to and when I joined the gel study they told me I had to test.

**SQT:** How big of an issue do you think HIV/AIDS is for people in your community and what makes it an issue of greater or lesser importance?

**KN:** As I see it, HIV is not the issue but seeing the sick people especially the males because if you tell them about testing they don’t want to hear about it. We ladies if we feel sick we go test first.

**SQT**: has the importance of HIV/AIDS changed in the community over the past few years and if so why?

**KN:** I see that it’s changed now before people did not understand HIV, you would be isolated in the community.

**SQT**: Is it more of an issue for some groups than for others, and if so, which groups and why?

**KN**: I know people who come up to you and tell they have HIV, so you can live with it and it’s manageable with pills. It’s the males that still get sick, they even believe they are bewitched.

**SQT**: Is there denial or do they accept that AIDS is an issue

**KN**: They accept it now before there was denial.

**SQT:** What is the reputation of the HIV testing locations in your community?

**KN**: Ok I will start with the clinic nearest to me, they don’t like going there because there is a lot of people who know you there, and they know if you there for HIV related matters because you get sent to a white house and everyone knows why people go there .So people then prefer to go to clinics that are far from their home.

**SQT:** Is testing fully confidential?

**KN**: Yes I think so, I’ve never heard that a nurse has spread rumours.

**SQT**: What are providers like?

**KN:** I think they are ok they keep the secrets of the patients, and the clinics you have to be separated to different areas.

**SQT**: What do you think are the greatest barriers to HIV testing in your community?

**KN**: Its fearing what people will say, if they see you going to test then what will they think, before they use to complain that Thiers too many pill

**SQT:** What structural factors (e.g. availability of services, lack of resources, lack of privacy in the community, etc.)?

**KN**: No I don’t see that.

**SQT:** What do you know about HIVST?

**KN**: I did not know anything

**SQT:** Where did you hear about HIVST?

**KN**: Firstly the way I see it, the thing to prick yourself with is painful, they could buy the kit and not use it.

**SQT:** What do you perceive as possible challenges to HIVST?

**KN**: It’s that only you will know your status nobody else will know, and some people don’t trust the nurses so.

**SQT:** What do you think would be the benefits of HIVST?

**KN**: Its private and I can even test my child at home.

**Participant: 0023**

**SQT:** Have you tested for HIV?

**NB:** Yes

**SQT:** If yes, what made you test?

**NB:** I wanted to know about my life status – HIV status awareness

**SQT**: What is your testing routine?

**NB**: For now it’s every month because I’m 5 months pregnant.

**SQT:** How big of an issue do you think HIV/AIDS is for people in your community and what makes it an issue of greater or lesser importance?

**NB:** Ja its important they do have fear you hear people say I went to test when they see a sick person they think to test and not just look on, maybe I too am ill I should check. – Fear of testing

**SQT:** Has the importance of HIV/AIDS changed in the community over the past few years and if so why?

**NB**: Ja, or people accept that they need to test, maybe if I’m positive I’m not alone maybe there is 90 of us that have it. Before they was fear.

**SQT:** Is it more of an issue for some groups than for others, and if so, which groups and why?

**NB:** The youth it’s not usually an old aunty of 1960 something falling ill, but it’s the young childbearing kids that get sick.

**SQT:** Is there denial or do they accept that AIDS is an issue?

**NB**: The way I see it they accept that it’s an illness that’s there, maybe its 10% of the population that doesn’t have anyone that’s taking treatment.

**SQT:** What is the reputation of the HIV testing locations in your community?

**NB**: If I’m sick or have flue before they can even touch me I have to test first, I can say a person who does not know their status is a person who does not want to. I think it’s been made easier when you go to the clinic for whatever reason they test you first. Unlike before where you would plan for a specific day to go to the clinic and test.

**SQT:** Is testing fully confidential? Explain.

**NB**: Ja because in the room it’s just you and the counsellor and I think they would lose their jobs if they went around and disclosed your status.

**SQT:** What are providers like?

**NB**: We have no problems with the providers.

**SQT:** What do you think are the greatest barriers to HIV testing in your community?

**NB**: Its fear of knowing, and seeing others get terminally ill.

**SQT:** What structural factors (e.g. availability of services, lack of resources, lack of privacy in the Community, etc.)?

**NB:** No it’s an open space and divided into different departments.

**SQT:** What do you know about HIVST?

**NB**: I only knew that you can get the kit at the pharmacy.

**SQT:** Where did you hear about HIVST?

**NB**: From my friend who works at clicks she said they are available and are simple like pregnancy tests to use.

**SQT:** What do you perceive as possible challenges to HIVST?

**NB**: The way I see, it will be like condoms those that really want to know will take it and use it and those who don’t won’t even take it.

**SQT:** What do you think would be the benefits of HIVST?

**NB**: It will help those who want to know where they stand with regards to HIV.

**Participant: 0024**

**SQT:** Have you tested for HIV?

**NP:** Yes I have tested

**SQT:** If yes, what made you test?

**NP**: I wanted to always know my status, and if I do have HIV then to get treatment.

**SQT:** What made you decide to test today?

**NP**: I wanted to know my status

**SQT:** what is your testing Routine?

**NP**: I test every 3 months, and since I’m on the injectable contraceptive I have to test regularly.

**SQT:** How big of an issue do you think HIV/AIDS is for people in your community and what makes it an issue of greater or lesser importance?

**NP**: It’s an issue because people date many people at the same time, and at times it’s for money and they can be careless.

**SQT:** Has the importance of HIV/AIDS changed in the community over the past few years and if so why?

**NP**: There’s treatments now.

**SQT:** Is it more of an issue for some groups than for others, and if so, which groups and why?

**NP**: It’s more of an issue for teenagers, like from 12 years and have sex without protection,

**SQT:** Is there denial or do they accept that AIDS is an issue?

**NP**: I honestly think that a person that would deny that HIV is not real would be wrong, it’s an illness that’s there.

**SQT:** What is the reputation of the HIV testing locations in your community?

**NP:** The clinic by my house really works because many people go there some come from far to get their treatment for HIV there.

**SQT:** Is testing fully confidential?

**NP**: Yea it is, they write the results down on paper give it to you and throw away the testing kit that they had used.

**SQT:** What are providers like?

**NP**: You are treated well, because when you go to the clinic you must feel at home, so they shouldn’t criticise or discriminate against you so they treat you well till you leave.

**SQT:** What do you think are the greatest barriers to HIV testing in your community?

**NP:** First of All when people find out that you have HIV, they gossip about you and stay away from you and you are left all alone and you cannot communicate with anyone, they don’t really understand the HIV so people don’t go and test because they fear other people’s attitudes towards them should they find out that they are positive.

**SQT**: What structural factors (e.g. availability of services, lack of resources, lack of privacy in the community, etc.)?

**NP**: The structure is quiet good, and you all go to the same area the cards are all the same so it’s just you and the doctor that know what it is that you came to do.

**SQT:** What do you know about HIVST?

**NP**: I heard that you can buy a kit at the shop

**SQT:** Where did you hear about HIVST?

**NP**: I saw and heard about it at the cosmetic store focus.

**SQT**: What do you perceive as possible challenges to HIVST?

**NP**: It has a good and a bad side, at the clinic the counsellor tests you and they can advise you that it’s not the end of the world you can continue, with HIVST you can harm yourself or commit suicide because you fear telling your family that you have HIV in some families some don’t understand this HIV thing that you are still the same as everyone else it’s just that you have HIV

**SQT:** What do you think would be the benefits of HIVST?

**NP**: It’s that you test with your partner and if you are sick then both of you can go on and take treatment

**Participant: 0025**

**PVK:** Have you tested for HIV before?

**V0025**: Yes I have been tested. I have tested 3 times at the mobile clinic while I was hired as a temp in one of the shops in my area.

**PVK:**  What made you decide to get tested at that time?

**V0025**: I just decided to get tested because some of my colleagues used to tell us that we should get tested so that we know our status. They used to tell us that they are taking HIV treatment. So when the mobile clinic came around, because they used to park next to the shop, I decided to get tested because it is always a good thing to know your status.

**PVK:** Okay, when last did you get tested?

**V0025**: Last year.

**PVK:**  So you did all these tests last year?

**V0025**: Yes. I did the first one and they told me that I should come back again after 3 months, I waited for three months and went back again. I tested and waited for another three months and I tested again. I last tested in July if I am not mistaken.

**PVK:** Other than that, you have never been tested before?

**V0025**: I tested when I had my child in 2006. It has been a while. I can’t even remember what exactly happened.

**PVK:** How big of an issue do you think HIV and AIDS is for people in your community?

**V0025:** It is a big issue in my area. Lots of people I know are taking treatment from the clinic. There are also those people who are working, who are unable to go collect their medication at the clinic, they give us their IDs to go collect the treatment for them because sometimes they can’t get time off. The clinic does not have a problem as long as you have her ID and your ID to show them that you know the person.

**PVK:** Do you think the importance of HIV has changed over the past few years or it has not changed?

**V0025:** Things have changed now because people are now taking treatment. During our mother’s time, in 2001, there was no treatment to supress HIV. Things are better now because people take treatment and recover well, as long as you take the treatment correctly and consistently. If you mix tablets with alcohol then it doesn’t work. People die because of that.

**PVK:** If HIV is still an issue, do you think HIV is more of an issue for certain groups than for other groups, for example, youth, adults, men or women?

**V0025:** In my area it is mixed. It is an issue for adult, young, men and women. I know a lot of people in my area who are taking treatment. This thing does not discriminate.

**PVK:** Do you think there is still a denial in some of your community members that AIDS is an issue? Are there people who are still in denial about AIDS?

**V0025:** I don’t know of anyone who is still in denial.

**PVK:** You mentioned that you tested at the mobile clinic, where else do people go for HIV testing?

**V0025**: We used to have a mobile clinic that used to come and park at the store nearby, but I have not seen the car in a while. They used to come and announce on the speaker calling people to come and get tested for HIV. Mpophomeni clinic is quite a long distance from my area.

**PVK:** But clinic does provide HIV testing and counselling?

**V0025**: Yes

**PVK:**  What is the reputation of mobile clinic and Mpophomeni when it comes to HIV testing? Are people comfortable to go to these facilities and is confidentiality maintained?

**V0025:** Like in the clinic, there is a place that we all know that it is for people who are coming to collect treatment. If people see you there, they would start gossip that they saw so and so at the clinic collecting treatment. There is a place allocated for collection of treatment, HIV testing etc.

**PVK:** Does that make people feel uncomfortable to go to the clinic for HIV testing?

**V0025**: Yes because sometimes people are reluctant to go because they know if they are seen, they would be considered as people who have HIV. Even us, if we go collect medication for our family members, people think it is our treatment, but I don’t care because I know my status and I know I don’t take treatment. But once you are inside, nobody knows what you are talking about, it is between you and the counsellor. The thing is people assume that if you go to that room, because they know that people who go there are mostly those that are infected, they assume that everyone who goes there is HIV positive.

**PVK:** What are service providers like at these facilities, like counsellors or nurses, when you go there for HIV testing?

**V0025**: There are good people. They are nice.

**PVK:** What do you think are the greatest barriers to HIV testing in your community?

**V0025**: I have heard many people saying they are scared. I don’t understand why they are scared because it is important to know your status.

**PVK:** You don’t think that maybe people’s attitudes and beliefs contributes in people not getting tested for HIV?

**V0025**: I would not know. I am not sure. I have only heard that they are scared to get tested. That is what they say.

**PVK:** Is it easy for people in your community to access HIV testing facilities?

**V0025**: For Mpophomeni clinic, it is easy for us to access it even though it is a bit of a distance

**PVK:** Are resources for HIV testing always available at the clinic?

**V0025**: There are instances where you find that they don’t have medication, especially HIV treatment, they would tell you to come the next day. There are issues there and there.

**PVK:**  But when it comes to HIV testing, do they ever run out of HIV testing kits?

**V0025:** No**,** I don’t know about kits.

**PVK:** Ok. Have you heard about HIVST before?

**V0025**: No. This is the first time I am hearing about this. - *? education/ awareness of HIVST*

**PVK:**  So as I have explained, with this kit you test yourself, you don’t have to come to the clinic, but what do you perceive as possible challenges to this kit?

**V0025**: I don’t foresee any challenges. I think this method is also good.

**Participant: 0026**

**SQT**: Have you tested for HIV?

**NN**: yes

**SQT:** If yes, what made you test?

**NN:** I wanted to know my status. – HIV status awareness

**SQT:** Routine 3 monthly testing?

**NN**: I test every 3 months but I last tested in June last year.

**SQT:** How big of an issue do you think HIV/AIDS is for people in your community and what makes it an issue of greater or lesser importance?

**NN**: The people I live with they see it as important but they are scared to test that what I hear from them. They say it’s better not to know because if they do know they will get ill and get stressed, I told a friend of mine about the importance of testing and she ended up testing and came back to tell me that the test went well, she saw the importance in the end.

**SQT**: Has the importance of HIV/AIDS changed in the community over the past few years and if so why?

**NN**: The way I see it people do not care to test, they always say that they are scared .Only when their bodies start to change, then they go for the test.

**SQT:** Is it more of an issue for some groups than for others, and if so, which groups and why?

**NN**: Teenagers, they are sexually active but do not protect themselves and also fall pregnant at a young age.

**SQT:** Is there denial or do they accept that AIDS is an issue?

**NN:** They accept it as an issue.

**SQT:** What is the reputation of the HIV testing locations in your community?

**NN**: What I can say, the location has a good reputation

**SQT:** Is testing fully confidential?

**NN:** Yeah I would say so.

**SQT:** What are providers like?

**NN:** They are good they make you comfortable and not to lose hope about the future.

**SQT:** What do you think are the greatest barriers to HIV testing in your community?

**NN**: Ok what I’ve heard, they will say I know how I carry myself and I also know how the person I’m with is carrying themselves and have that as an excuse not to test.

**SQT:** What structural factors (e.g. availability of services, lack of resources, lack of privacy in the community, etc.)?

**NN**: It’s a safe place that’s all right, but at times you go for testing there and you find in the room there’s a counsellor and another member of stuff in the room, and you do the test there while the other one is sitting listening in on the conversation and that made me so uncomfortable I actually wanted to stop because I knew that my results were not private.

**SQT:** What do you know about HIVST?

**NN:** I know nothing.

**SQT**: Where did you hear about HIVST?

**NN**: I heard for the first time from you guys.

**SQT:** What do you perceive as possible challenges to HIVST?

**NN:** Maybe its fear, and for me I didn’t think this would be a good thing for people ,but as time goes on people will find help in these kits.

**SQT**: What do you think would be the benefits of HIVST

**NN:** What could I say…uhhhhm it’s that people will know how their status is, and some people fear being tested by another person .I also think there will be harm ,if a person finds that they are positive it could be hard on them, who would they tell first? Maybe if they had been tested at a clinic they would have been told what to do next and given comfort.

**Participant: 0027**

**PVK:** Have you tested for HIV?

**E0027**: Yes

**PVK:**  What made you get test for HIV?

**E0027**: I usually get tested because I want to know my status. – HIV status awareness

**PVK:** Okay, how often do you get tested?

**E0027**: Twice a year.

**PVK:** What made you agree to get tested today?

**E0027**: Because I like getting tested for HIV. – HIV status awareness

**PVK:** How often do you get tested for HIV?

**E0027**: After every 3 months.

**PVK:** Ok**.** How big of an issue do you think HIV is for people in your community?

**E0027:** Yes it is an issue because there are people that I know, some of them have since died because they did not take treatment. It is an issue. – Poor Health Seeking Behaviour

**PVK:** Do you think the importance of HIV has changed in your community over the past few years?

**E0027:** I think it has changed because there are lots of things that being done in the community to educate people on HIV.

**PVK:** Do you think HIV is more of an issue for certain groups? Which group do you think is more affected by HIV than others?

**E0027:** I think HIV affects everyone, but mostly it is the youth.

**PVK:** Why do you think youth is more affected than other groups?

**E0027:** Because I know lots of them who have died and some are sick.

**PVK:** Do you think there is a denial in your community that AIDS is an issue?

**E0027**: I don’t think so because everyone is aware of HIV**.** People learn about it all over radio, TV and newspapers.

**PVK:**  Where do you usually go for HIV testing?

**E0027**: I usually for to Polly Clinic.

**PVK:**  Your local clinic?

**E0027**: Yes.

**PVK:** What is the reputation of HIV testing facilities that you use in your community?

**E0027:** I wouldn’t comment on that.

**PVK:**  Do you think confidentiality is maintained at this facility when people go for HIV testing?

**E0027**: Yes I think so.

**PVK:** How are the people who provide HIV testing like at Polly Clinic?

**E0027:** They are good people.

**PVK:** What do you think is the reason people don’t like to use their local clinics for HIV testing?

**E0027**: I think they are scared that they might get positive results. They prefer not to know their status thus knowing.

**PVK:** Do you think people’s attitudes and beliefs are barriers to HIV testing?

**E0027**: I think that could be the factor because I have heard people saying sometimes they call your name to come and take your treatment while you are sitting in a queue with everybody around. Some people don’t like that as it identifies them to other people that they are positive.

**PVK:** In terms of structural factors, it easy for people in your community to access the local clinic for HIV testing?

**E0027**: Yes it is easy to access the clinic.

**PVK:** And the privacy is maintained?

**E0027**: But there is a notice outside the door that indicate that people who go there are coming for treatment. Even if you leave the room, people look at you suspiciously wondering whether you are positive or not.

**PVK:** Ok. Do you know about HIVST?

**E0027**: Yes

**PVK:** Where you learned about this method?

**E0027**: I read about it on the newspaper.

**PVK:** What do you perceive as a possible challenge to this HIVST kit?

**E0027:** The challenge would be that no one would give you counselling because you need to get counselling before you can do HIV testing. If you get positive results, the counsellor gives you counselling before giving you the results, if you get this kit and you use it at home without counselling, you might find it difficult to deal with the results because they will be no one to counsel you. Lack of counselling, Social Harm

**PVK:** Ok, what do you think would be the benefits of using this HIVST kit?

**E0027:** The benefits would be that for those people who are scared to go to the clinic for HIV testing, they would have a chance to do it at home, on their own without anyone knowing about it. People are scared to go to clinic because of how people would think of them if they find out that they are positive, it would be a good thing for them to do this at home.

**Participant: 0028**

**SQT:** Have you tested for HIV?

**NG:** yes

**SQT:** If yes, what made you test?

**NG:** I just wanted to know my status

**SQT:** What made you decide to test today?

**NG:** It’s a requirement for this study

**SQT:** Routine 3 monthly testing?

**NG:** I test myself at work, I’ve tested maybe 4 time in the past year.

**SQT:** How big of an issue do you think HIV/AIDS is for people in your community and what makes it an issue of greater or lesser importance?

**NG:** It’s important, it’s just that the stigma that was there before is no longer there, there’s an acceptance and knowledge of which channels to follow to get treatment. – reduced stigma

**SQT:** Has the importance of HIV/AIDS changed in the community over the past few years and if so why?

**NG:** The importance has not changed people are also aware of the opportunistic infections so if one has TB they also test for HIV.

**SQT:** Is it more of an issue for some groups than for others, and if so, which groups and why?

**NG**: It’s an issue for everybody.

**SQT:** Is there denial or do they accept that AIDS is an issue?

**NG:** There is acceptance.

**SQT:** What is the reputation of the HIV testing locations in your community?

**NG:** Its ok it’s just that they divide the people and give different coloured stickers for different places, so everyone knows that if you have a green sticker that means that you are going to fetch your treatment. There’s no secret as to what you came to do.

**SQT:** Is testing fully confidential?

**NG:** Yes

**SQT:** What are providers like?

**NG:** They are ok, I have worked with them and found them to be ok.

**SQT:** What do you think are the greatest barriers to HIV testing in your community?

**NG:** I think its fear, and when you go get your treatment everyone at the clinic will see that you came to get HIV treatment**.** So people end up not testing at the clinics nearest to them due to that fear.

**SQT:** What structural factors (e.g. availability of services, lack of resources, lack of privacy in the community, etc.)?

**NG:** That could also be a factor because the clinic is quiet small and is surrounded by bushes and skwatta camps, so there is a lot of robbery that happens around there**.**

**SQT:** What do you know about HIVST?

**NG**: I already knew that its effective and you can trust the results.

**SQT**: Where did you hear about HIVST?

**NG:** I heard in my class 2 weeks back.

**SQT:** What do you perceive as possible challenges to HIVST?

**NG:** For instance you get one kit, and you get home and put the samples in the wrong holes.

**SQT:** What do you think would be the benefits of HIVST

**NG:** It keeps your business private if you are the kind of person that like to keep your results secret.

**Participant: 0029**

**SQT:** Have you tested for HIV?

**LK**: Yes

**SQT:** If yes, what made you test?

**LK**: Uhhhm I was pregnant and they said I had to do a HIV test.

**SQT:** What made you decide to test today?

**LK**: It been a while since I last tested – routine testing

**SQT:** Routine 3 monthly testing?

**LK:** I don’t have a routine, maybe once or twice a year

**SQT:** How big of an issue do you think HIV/AIDS is for people in your community and what makes it an issue of greater or lesser importance?

**LK**: It’s an issue because in most households they have had someone sick or die from HIV, I think most people are affected.

**SQT**: has the importance of HIV/AIDS changed in the community over the past few years and if so why?

**LK**: Yes, I think people are taking it like it’s an illness that’s out there and it kills, I think now people know more about it than they did before.

**SQT:** Is it more of an issue for some groups than for others, and if so, which groups and why?

**LK**: I think it’s an issue for women and teens maybe because they take their health seriously and go the clinics to get treatment and tests. The men hide they fear being seen doing such things.

**SQT:** Is there denial or do they accept that AIDS is an issue?

**LK**: I think now there is acceptance because they all can see what HIV can do, in the past I don’t think people know what it really was and the proper ways to protect themselves.

**SQT:** What is the reputation of the HIV testing locations in your community?

**LK**: Well I would say they are ok.

**SQT:** Is testing fully confidential?

**LK**: I think so but you can never know that for sure

**SQT:** What are providers like?

**LK**: They are fine but some can be rude.

**SQT:** What do you think are the greatest barriers to HIV testing in your community?

**LK**: Eish I think most people are scared to know their status, you know especially the ones that have been behaving bad sexually, you know you fear that you could be sick and have to be on treatment for the rest of your life.

**SQT:** Are people’s attitudes and beliefs about HIV barriers?

**LK**: Ja I think so, there’s a lot of education about HIV but some people are just still in the dark

**SQT:** What structural factors (e.g. availability of services, lack of resources, lack of privacy in the community, etc.)?

**LK**: The clinic itself is good, at times there are shortages of medicines

**SQT:** What do you know about HIVST?

**LK**: I did not know anything – Knowledge about HIVST

**SQT:** Where did you hear about HIVST?

**LK:** From you guys when you called me.

**SQT:** What do you perceive as possible challenges to HIVST?

**LK:** I think suicide, I mean you will be doing this alone with no one to counsel you.

**SQT:** What do you think would be the benefits of HIVST?

**LK:** This can give you privacy, and you can decide when and who you want to disclose to**.**

**Participant: 0030**

**PVK:** Have you tested for HIV before?

**E0030**: Yes

**PVK:** What made you get test for HIV at that time?

**E0030**: I decided to do a random test after I heard from nursing school that now we can buy HIV testing kit from the pharmacy and perform test at home. I decided to go to the pharmacy and bought the kit and I tested myself at home.

**PVK:** Which pharmacy did you buy the kit from?

**E0030**: I can’t remember the name but it is situated next to Mr Price at The Wheel in town.

**PVK:** How was the reaction of the pharmacist when you ask for the self-testing kit?

**E0030**: I did not even notice their reaction, because I was wearing my nursing uniform, maybe they did not mind because they saw that I was wearing nursing uniform.

**PVK:** How much did you pay for it?

**E0030**: Just less than R100, I think. I can’t remember exact amount.

**PVK:** Okay, how often do you get tested?

**E0030**: Twice a year.

**PVK:** So what is the reason you agree to get tested today?

**E0030**: I still want to know my status because I have not tested this year.

**PVK:** Ok**.** How big of an issue do you think HIV is for people in your community, if it is an issue?

**E0030:** The issue that we have is that people are scared to get tested especially in clinics because they are afraid of being stigmatised by nurses who are going to talk about them to other people. That is the main problem we have especially at Adams, otherwise people do go to clinics for HIV testing; those who don’t mind what other people say about them.

**PVK: D**o you think the importance of HIV has changed in your community over the past few years?

**E0030:** I think it has changed because in the past, people were not going to clinics for HIV testing and even for treatment but nowadays infants are tested at birth to detect the virus so that they can start treatment early and live longer. Children are now going to start treatment early and they can live longer unlike previous years when people were not taking treatment even when the treatment was available because of lack of knowledge.

**PVK:** For those communities that HIV is an issue, do you think it is more of an issue for certain groups? Which group do you think is more affected by HIV than others?

**E0030:** For me I believe it is the youth. It is difficult to just wake up and decide to get tested. You want to go with a friend, especially if you know that you had unprotected sex, it is not easy. You need someone to give you support and if you are young and happen to get positive results, you can’t even disclose your status to your family. It becomes difficult.

**PVK:** Do you think there is a denial in your community that AIDS is an issue?

**E0030**: Nowadays, I don’t think so because majority of people are aware of HIV. If you go to clinics, you would realise that people do get tested for HIV. The clinic staff do explain the whole process even about the treatment. There is no issue if they get proper counselling but some people especially young people still don’t want to accept. It usually happens to those people who have had sex with one partner, they want to know how come one gets infected after sleeping with only one person.

**PVK:**  In general, what is the reputation of HIV testing facilities that people use in your community, whether clinic or mobile, etc?

**E0030:** I think the clinics are good though I think mobile clinics are more visible because they go to people in the community.

**PVK:** Do you think people’s attitudes and beliefs are barriers to HIV testing?

**E0030**: In the past we used to hear a lot about nurses who scold young people who come for HIV testing, they used to judge them, and that is the reason young people were not going to clinics for HIV testing because nurses would ask them why they are getting tested, and they used to accuse them of sleeping around. They even accuse them for coming for family planning.

**PVK:**  Has that changed now?

**E0030**: Yes it has changed.

**PVK:** Is confidentiality maintained at this HIV testing facilities?

**E0030**: I think majority of service providers do maintain confidentiality but some people, for some reason, I don’t know but they do share information about their clients. Like for example, if I come for HIV testing and the nurse is from my neighbourhood, she can discuss my result with her colleagues as soon as you leave the room.

**PVK:** What do you think is the greatest barriers to HIV testing in your community?

**E0030**: I think it is stigma attached to testing and also sometimes people believe that if you have one partner and you sleep with that partner only, they don’t see the need to get tested because they don’t see themselves getting infected. They always think that they are safe. Others don’t go for testing because of attitude of nurses at the clinics.

**PVK:** Do you think structural factors are also a barrier to HIV testing? Like how far one has to travel to get to the clinic and maybe when you get there are you happy with privacy provided at the clinics?

**E0030**: I think it is easy these days because of mobile clinics, people don’t have to walk long distances to get to the clinic, they can wait for mobile clinic to come around their area.

**PVK:** You said you already know about HIVST, right?

**E0030**: Yes

**PVK:** When and where did you first heard about it?

**E0030**: I heard about it at the training college, at Edington.

**PVK:** Now that you know about it, what do you perceive as a possible challenge to this HIVST kit?

**E0030:** I think the problem is going to be the denial. If people find out that their results are not in their favour, they will buy another kit thinking that maybe it is because have not been tested by the nurse. They would think maybe they made a mistake somewhere. – Usability of HIVST kit, Accuracy

**PVK:** Ok, what do you think would be the benefits of using this HIVST kit?

**E0030:** The benefits would be that you don’t have to worry about your results being discussed to other people that you don’t want them to know. Other benefits would be that you do this test at the comfort of your own private space. If you are tested positive, you can then go to the clinic just to get confirmation so that you can start treatment early.

**Participant: 0031**

**PVK:** Have you tested for HIV before?

**E0031**: I have never tested.

**PVK:** Not even once?

**E0031**: No, I have never.

**PVK:** What is the reason you never tested?

**E0031**: I am scared.

**PVK:** Today you have decided to come for HIV testing, why this time?

**E0031**: I have realised that it is wrong not to know my HIV status, so I think it is about time now.

**PVK:** Ok**.** How big of an issue do you think HIV is for people in your community, if it is an issue?

**E0031**: I don’t think it is an issue. No, it is not.

**PVK:** Do you think the importance of HIV has changed in your community over the past few years?

**E0031**: I think it has changed because people used to be scared of HIV, now everybody knows about it and they are not scared anymore.

**PVK:** For communities that HIV is an issue, do you think it is more of an issue for certain groups? Like men? Women? Young? Old? Which group do you think is more affected by HIV?

**E0031:** HIV does not discriminate. It affects everyone equally.

**PVK:** Do you think there is still a denial in your community that AIDS is an issue?

**E0031**: There are few people who are still in dial. I think they need more education on AIDS, more especially elders.

**PVK:** I know you have not been tested for HIV, but from what you have heard and maybe observed, what is the reputation of HIV testing facilities that people use in your community?

**E0031:** I have not heard much about it. I don’t know.

**PVK:** What do you think the service providers are like in the clinics?

**E0031**: I think they are good.

**PVK:** What do you think is the greatest barriers to HIV testing in your community?

**E0031**: I think maybe people are scared of knowing that they are infected. They say they are not ready, I know nobody would be ready for positive results but I think they get scared. Also lack of knowledge contributes to people not getting tested.

**PVK:** Do you think people’s attitudes and beliefs also are barriers to HIV testing?

**E0031**: I think so because sometimes people judge you and shun away from you if they know you are positive.

**PVK:** Do you think structural factors are also a barrier to HIV testing? For example, the distance, lack of resources, etc.

**E0031**: It is easy to get to the clinic. It is not far.

**PVK:** What do you think of privacy in this testing facilities?

**E0031**: I know someone who told me that counsellors are good and there is privacy.

**PVK:** Have you heard about HIVST?

**E0031**: No. I am hearing this for the first time.

**PVK:** Ok, as I have explained earlier that it is a kit that you can buy it at the pharmacy and you can use it at home, what do you perceive as a possible challenges to this HIVST kit?

**E0031:** The challenge would be that maybe I would conduct test without washing my hands.

**PVK:** What happens if you do test without washing your hands?

**E0031**: I think if I test myself without washing my hands, if maybe my hands are dirty, maybe that would affect the results, I am not sure if that is the case, I am just thinking.

**PVK:**  Anything else you think might be a possible challenge to this HIVST kit?

**E0031**: I think if people buy this HIVST kit they might be shocked if results are not in their favour. Some may lock themselves in the room or collapse, something like that. I think it will be better if everyone would start getting tested at the clinic then after that people can continue with self-testing every three months.

**PVK:** Ok, despite all these challenges, what do you think would be the benefits of using this HIVST kit?

**E0031:** There are benefits because some people don’t have time to go to clinics because they work during the week. Some clinic don’t open on weekends, so at least if you buy this kit you can use it on your spare time.

**Participant: 0032**

**PVK:** Have you tested for HIV before?

**E0032**: No

**PVK:** Not ever?

**E0032**: No.

**PVK:** Why you never tested?

**E0032**: I was scared.

**PVK:** What made you decide to get tested today?

**E0032**: I had unprotected sex.

**PVK:** When?

**E0032**: About a month ago.

**PVK:** Ok**.** How big of an issue do you think HIV is for people living in your community?

**E0032**: It is a big problem.

**PVK:** Why do you think it is a big problem?

**E0032**: Because maybe people don’t take care of themselves or maybe they are ignorant, I don’t know, but people don’t care.

**PVK:** Do you think the importance of HIV has changed in your community over the past few years?

**E0032**: I think it has not changed. It is still the same.

**PVK:** Why do you say so?

**E0032**: I am just saying this because I don’t think people care.

**PVK:** Have you heard about HIVST?

**E0032**: No. I am hearing this for the first time.

**PVK:** Ok, as I have explained what it is and where you can get it, what do you perceive as a possible challenges to this HIVST kit?

**E0032:** I don’t think they will be any challenges if people are trained on how to use this test. It is going to be up to them to buy the kits.

PVK**:** What do you think would be the benefits of using this HIVST kit?

**E0032:** People would benefit because they would be able to test themselves without going to the clinic.

**Participant: 0033**

**PVK:** Have you tested for HIV before?

**E0033**: Never

**PVK:** What made you decide to get tested today?

**E0033**: Someone recommended that I come to CAPRISA.

**PVK:** Why did that person recommend you to come to CAPRISA?

**E0033**: I have been wanting to get tested but I was scared. I realised at that time point I had to get tested because I am now grown up and I need to know my status.

**PVK:** Ok**.** How big of an issue do you think HIV is for people living in your community?

**E0033**: It is a big issue. Most people take it as a serious issue because we have seen so many people dying in front of us due to HIV. Most people are really worried and scared.

**PVK:** Do you think maybe the importance of HIV has changed in your community over the past few years?

**E0033**: I don’t think it has changed even though majority of people are still scared of it.

**PVK:** Do you think HIV is more of an issue for certain groups? Or it is the same for all groups?

**E0033**: It affects all of us equally.

**PVK:** Do you think there is still a denial in your community that AIDS is an issue?

**E0033**: In this day and age, I don’t think there are people who still denies that there is HIV. I don’t think so.

**PVK:** In general, what is the reputation of HIV testing facilities that people use in your community?

**E0033:** From the people I know, I don’t know anyone who has ever complained that they were ill-treated at the clinic while coming for HIV testing.

**PVK:** They never complain about service providers or even about privacy?

**E0033**: No they never complain about anything.

**PVK:** What do you think is the greatest barriers to HIV testing in your community?

**E0033**: People are scared of facing reality, especially if they know their past behaviour and all the things they have done, they don’t want to find out that they are positive.

**PVK:** Do you think maybe people’s attitudes and beliefs about HIV are also barriers to HIV testing?

**E0033**: Nowadays we know a lot about HIV. It is not like we used to hear about in the past, that if you have HIV it means you are going to die tomorrow. People understand.

**PVK:** Do you think structural factors are also a barrier to HIV testing? Is it easy for people to get to the clinic and are resources always available in these testing facilities?

**E0033**: Yes it is easy to access the clinic from where I come from. People never complained.

**PVK:** Have you heard about HIVST?

**E0033**: No. I am hearing this for the first time that there is something like that.

**PVK:** Ok, as I have explained what it is and where you can get it, what do you perceive as a possible challenges to this HIVST kit?

**E0033:** I think it is going to be a problem because you need to be tested by someone who is an expert and knows what she is doing by giving you proper counselling especially if your status is positive. If you do self-testing, you would not get that support that you get from the counsellor.

**PVK:** In terms of benefits for those would be using this kit, what do you think would be the benefits of using this HIVST kit?

**E0033:** I think majority of people are lazy to come to clinics. If you don’t want to come to clinic, you can buy this kit and do self-testing. I think I also like this method already because I know I can do this on my own, I don’t have to worry about someone finding out about my results first. I can keep my results to myself and be confident that no one would know. I think that is a benefit of using this test.

**Participant: 0034**

**SQT:** Have you tested for HIV?

**GPM:** No I’ve never tested for HIV before.

**SQT:** If no, what prevented you from testing?

**GPM:** Fear of knowing my status, it’s never really been a priority for me.

**SQT:** What made you decide to test today?

**GPM:** Well I felt encouraged because my room mates come here (Caprisa) and they say it’s good and they get to know their status, so I was the only one who was left out, so I decided to come today.

**SQT:** How big of an issue do you think HIV/AIDS is for people in your community and what makes it an issue of greater or lesser importance?

**GPM:** I’m not really sure but I think it is an issue within my community, I haven’t really been out there in the community so I haven’t paid much attention**.**

**SQT:** Has the importance of HIV/AIDS changed in the community over the past few years and if so why?

**GPM:** Well in my neighbourhood I don’t think so I don’t see people empowering HIV testing that much.

**SQT**: Is it more of an issue for some groups than for others, and if so, which groups and why?

**GPM:** ahhm I think it’s an issue for younger people they don’t test that much I haven’t seen anyone talk about it or in my age group besides my friends because they more smart I haven’t seen guys its mostly girls and stuff.

**SQT:** Is there denial or do they accept that AIDS is an issue?

**GPM:** Ja I think there’s denial.

**SQT:** What is the reputation of the HIV testing locations in your community?

**GPM:** Joh I don’t think there’s any I haven’t seen any in my community I haven’t seen any promos about HIV.

**SQT:** Do you know of any centre/clinic where you can test within your community?

**GPM:** No

**SQT:** What do you think are the greatest barriers to HIV testing in your community?

**GPM:** I think it’s the stigma attached and it feels like everyone will know your status when they see you going to test.

**SQT:** Are people’s attitudes and beliefs about HIV barriers?

**GPM:** Yes I think so.

**SQT:** What structural factors (e.g. availability of services, lack of resources, lack of privacy in the community, etc.)?

**GPM:** Yes I think so in my community there’s a lot of black people so I don’t think there is a lot of privacy.

**SQT:** What do you know about HIVST?

**GPM:** Nothing

**SQT**: Where did you hear about HIVST?

**GPM:** First time here with you.

**SQT:** What do you perceive as possible challenges to HIVST?

**GPM:** Firstly you are doing it alone so that means you the only one that knows and you will keep it to yourself and you are going to get stressed and it’s going to build up and you will become sick.

**SQT:** What do you think would be the benefits of HIVST?

**GPM:** Ok well you will know if you have HIV that will mean you will take care of yourself and use protection.

**Participant: 0035**

**SQT:** Have you tested for HIV?

**TTC:** No

**SQT:** If no, what prevented you from testing?

**TTC:** I was just scared.

**SQT**: What made you decide to test today?

**TTC:** Well it’s because I’ve had the problem of losing weight, so I wanted to know what’s going on, I always wished to test, so some lady told me I should come here

**SQT**: How big of an issue do you think HIV/AIDS is for people in your community and what makes it an issue of greater or lesser importance?

**TTC**: In my community I do not think that it is that much of an issue because most of the people with HIV take their ARV’s and do not fear telling others that they have HIV.

**SQT:** Has the importance of HIV/AIDS changed in the community over the past few years and if so why?

**TTC**: Yes it has because they saw many die right in front of them because of their fear of the disease. Now they take the disease more seriously

**SQT**: Is it more of an issue for some groups than for others, and if so, which groups and why?

**TTC:** It’s mostly the teenagers, because most of the people that I know who are infected are nearly in my age group.

**SQT**: Is there denial or do they accept that AIDS is an issue?

**TTC:** There is acceptance even though some are still scared to disclose their status.

**SQT:** What is the reputation of the HIV testing locations in your community?

**TTC:** The clinic in my community has a bad reputation, they do not treat the patients well and the nurses are not good.

**SQT**: Is testing fully confidential?

**TTC**: I do not know, what makes me fear testing there is because there are nurses there that are my neighbours.

**SQT**: What are providers like?

**TTC:** Most of them have an attitude, they do not want to be told anything they make you feel that they are so educated they are that sort of people.

**SQT**: What do you think are the greatest barriers to HIV testing in your community?

**TTC:** It’s the fear and people do not like taking pills**.**

**SQT:** What structural factors (e.g. availability of services, lack of resources, lack of privacy in the community, etc.)?

**TTC:** No

**SQT:** What do you know about HIVST?

**TTC:** Nothing

**SQT:** Where did you hear about HIVST?

**TTC:** I heard from you today.

**SQT**: What do you perceive as possible challenges to HIVST?

**TTC:** Testing alone a person could kill them self if the result is positive, where it’s much better if there’s another person to advise them.

**SQT**: What do you think would be the benefits of HIVST?

**TTC:** People are not the same some are able to bare the result if they do it on their own in private rather than going to a person where they could feel ashamed.

**Participant: 0036**

**PVK:** Have you tested for HIV before?

**E0036**: Never

**PVK:** You never tested before at all?

**E0036**: Never

**PVK:** What made you decide to get tested today?

**E0036**: I have a boyfriend whom I had sex with without a condom. – *risky sexual behaviour*

**PVK:** Was it your first time to have sex without a condom?

**E0036**: It was my first time to have sex without a condom.

**PVK:** Ok**.** How big of an issue do you think HIV is for people living in your community?

**E0036**: It is a big issue. There is someone I know, a woman who recently died due to HIV, we buried her recently. There is also another one who just passed away, she passed away day before yesterday, and she also died of HIV. So it is a big problem.

**PVK:** Do you think maybe the importance of HIV has changed in your community over the past few years?

**E0036**: It has changed.

**PVK:** It has changed in what way?

**E0036**: Most people are now taking care of themselves by taking treatment. People don’t wait until they are sick to get treatment.

**PVK:** Do you think HIV is more of an issue for certain groups? Or it is the same across all groups?

**E0036**: It affects mostly older people.

**PVK:** You mean old as in grandmothers and grandfathers?

**E0036**: Mothers especially.

**PVK:** What about males

**E0036**: Not, not that much.

**PVK:** Do you think there is still a denial in your community that AIDS is an issue?

**E0036**: No, I don’t think so.

**PVK:** In general, what is the reputation of HIV testing facilities that people use in your community?

**E0036**: I think they are good. They treat people well.

**PVK:**  Do you think your testing is kept fully confidential?

**E0036**: Yes they do keep confidentiality.

**PVK:** What are providers like?

**E0036**: They are good people.

**PVK:** What do you think is the greatest barriers to HIV testing in your community?

**E0036**: They are scared.

**PVK:** What are they scared of?

**E0036**: They are scared that people would see them and know that they are sick. They are scared that if they are tested positive, they would have to take treatment.

**PVK:** Do you think maybe people’s attitudes and beliefs about HIV are also barriers to HIV testing?

**E0036**: I think so because some people don’t want to associate with you if they know you are positive.

**PVK:** Do you think structural factors are also a barrier to HIV testing in terms of resources always available in these testing facilities?

**E0036**: Yes that is true.

**PVK:** Have you heard about HIVST?

**E0036**: No.

**PVK:** It is the first time you are hearing this.

**E0036**: Yes.

**PVK:** Ok, as I have explained what it is, what do you perceive as a possible challenges to this HIVST kit?

**E0036:** I think the reason people are not testing is because they are scared. If you do self-testing at home, you are not going to accept results if you are positive and maybe you would think of committing suicide. It is better to go to the clinic because you would have someone to talk to, giving you counselling and advise on how to take treatment unlike doing it at home.

**PVK:** In terms of benefits for those would be using this kit, what do you think would be the benefits of using this HIVST kit?

**E0036:** People don’t have to go to the clinic. They can buy the kit and do test at home. They can go to the clinic to get treatment if they are positive.

**Participant: 0037**

**PVK:** Have you tested for HIV before?

**E0037**: I have never tested before

**PVK:** You never tested before at all?

**E0037**: I am really scared of testing for HIV. There are times when I get stomach cramps, something like that, I think about things that I have done in the past and I get nervous if I think about getting HIV test because I wonder what if I get positive results. Those things make me nervous.

**PVK:** What really made you scared to get tested?

**E0037**: I have been worried that if I get tested positive, that would mean the end of my life. That made me really nervous.

**PVK:** But you know that is not the case anymore. With treatment available, people are now living longer and healthy. That used to happen long time ago when treatment was not available.

**E0037**: Yes I know. I grew up knowing that if you are positive, you only have few years to live. That is what I knew.

**PVK:** At least now you know that people can live even more than 20 years if they take treatment correctly and take care of themselves.

**E0037**: Yes

**PVK:** So what made you decide to get tested today?

**E0037**: I was chatting to my friend the other day, he told me that he was at the clinic for HIV testing and he even showed me the results. He told me that he has made the decision to get tested every 3 months. He encouraged me to get tested as well, so I decided that I was going to get tested. I told myself that if I get tested positive, I would have to accept it, at least I am not going to die, I will take treatment just like other people who are living with the disease. It all depends on how you take it. if you accept the situation, you can live longer.

**PVK:** Ok**.** How big of an issue do you think HIV is for people living in your community?

**E0037**: It is a big issue. I know a lot of people who are already infected. I am now even scared to date. Even girls who are younger than me are also infected because they drink alcohol and sleep around with anyone. I think majority of people are now infected, even younger people. You can not trust your girlfriend just because she is still young. Those are the most dangerous ones.

**PVK:** Do you think maybe the importance of HIV has changed in your community over the past few years?

**E0037**: It is not the same anymore. There is a difference. I remember I used to stay with one guy who was also positive. He used to take a lot of tablets and I used to help him in reminding him to take his tablets. I think he was taking about 8-10 tablets at a time. It really made me sad to see him taking tablets. I was wondering what he had done to deserve that. But now people are taking few tablets and they know the importance of taking treatment.

**PVK:** Do you think maybe HIV is more of an issue for certain groups or it is the same across all groups?

**E0037**: Nowadays we know that HIV affects everyone. It does not discriminate.

**PVK:** Do you think there is still a denial in your community that AIDS is an issue?

**E0037**: There are people who are still in denial. There are people who are still believe that HIV is spread by these people who are using injection.

**PVK:** You mean drug dealers?

**E0037**: No, people believe that if they go to the clinics they are going to get infected because clinics use injections with HIV. They believe that this is done in purpose to infect as many people as they can, especially black people.

**PVK:** In general, what is the reputation of HIV testing facilities that people use in your community?

**E0037**: I recently moved to Durban, but from where I come from, I know someone who recently tested. He told us that if you are sick and you go to the clinic, they now tell you that you have to get tested for HIV before they even treat you. They can’t even touch you unless they test you for HIV then they can treat you.

**PVK:** So people are forced to get tested for HIV in order for them to be treated?

**E0037**: Yes, it looks like that because they tell you that they have to test you before they treat you.

**PVK:** So do you think HIV testing is kept fully confidential in these clinics?

**E0037**: I think they do keep your results confidential. It is up to you to disclose your status to people you trust but they are not allowed to share your results with anyone else. That would be wrong. That would affect you emotionally knowing that other people know about your status and they are gossiping about you.

**PVK:** What do you think is the greatest barriers to HIV testing in your community?

**E0037**: They are scared to find out that they are positive. They don’t want to face reality. There are also other people who do not believe in clinics. They use traditional medicines if they get sick, so those people would never go to clinic for HIV testing.

**PVK:** Do you think maybe people’s attitudes and beliefs about HIV are also barriers to HIV testing?

**E0037**: That could be true. I do believe that sometimes people don’t get tested for HIV because they are scared that they would be judged that they got infected because they were sleeping around.

**PVK:** Are structural factors also a barrier to HIV testing in terms of availability of resources in these testing including privacy?

**E0037**: To tell you the truth, I don’t really use clinics. My mother used to ask me to take my younger brother to the clinic if he was sick, otherwise I don’t really use the clinic.

**PVK:** Have you heard about HIVST?

**E0037**: No, I have never heard about it. For me, I would not have thought that this is something that you can have access to so easily.

**PVK:** Ok, what do you perceive as a possible challenge to this HIVST kit?

**E0037:** I think it is not going to be easy to use this kit if you are someone who is already scared to get tested for HIV. The only person who can buy this kit is someone who has been tested before, who know his status. Someone who wants to use this kit in the future to make sure that he does not have sex with a partner who has not been tested. He can take this test and ask partner to do HIV before they have sex. But otherwise if you have not used this before, I think some people would be reluctant to use it.

**PVK:** For those who would like to use this kit, what do you think would be the benefits of using this HIVST kit?

**E0037:** I think for those it would be a good thing because you can buy this for you and your girlfriend, and you know that you would have to ask your girlfriend to get tested before you can have sex with her, as long as the girlfriend also understand and agree to use the kit.

**Participant: 0038**

**PVK:** Have you tested for HIV before?

**E0038**: No, I have never.

**PVK:** What is the reason you never tested?

**E0038**: I was scared.

**PVK:** What made you decide to get tested today?

**E0038**: Because I told my friend that I have never tested before and she encouraged me to get tested.

**PVK:** Ok**.** How big of an issue do you think HIV is for people living in your community?

**E0038**: It is not a big issue, people are more afraid of TB than HIV.

**PVK:** Why do you think people are more afraid of TB than HIV?

**E0038**: I know a lot of people who have TB.

**PVK:** Do you think maybe the importance of HIV has changed in your community over the past few years?

**E0038**: It is important that people know their status so that they can take treatment.

**PVK:** Do you think maybe HIV is more of an issue for certain groups or it is the same across all groups?

**E0038**: In most cases it affects certain groups, it affects the youth.

**PVK:** Why do you think so?

**E0038**: Because there are the ones who are more sexual active.

**PVK:** Do you think there is still a denial in your community that AIDS is an issue?

**E0038**: There are people who are still in denial. Those who are not infected believe that there is no HIV. I was also in denial that HIV exist.

**PVK:** In general, what is the reputation of HIV testing facilities that people use in your community?

**E0038**: They say it is not scary to get tested in the clinics.

**PVK:** What do they say about service providers in these facilities? Like counsellors who provide HIV testing.

**E0038**: They say they are nice people.

**PVK:** Is HIV testing kept fully confidential in these HIV facilities?

**E0038**: Yes.

**PVK:** What do you think is the greatest barriers to HIV testing in your community?

**E0038**: They are scared to get HIV positive results. It is scary to know that you are infected with HIV because you worry that you are going to live with it for the rest of your life. It is also difficult to disclose your status to your family.

**PVK:** Do you think maybe people’s attitudes and beliefs about HIV are also barriers to HIV testing?

**E0038**: Yes I think so. Some people don’t know that you can get infected with HIV even if you don’t sleep around.

**PVK:** Do you think structural factors also a barrier to HIV testing? For example, are HIV testing kits always available in these facilities and is there privacy?

**E0038**: Of course there is privacy but the disadvantage is that it is always full because it is in rural areas.

**PVK:** Have you heard about HIVST?

**E0038**: No, I have never heard about it.

**PVK:** Ok, what do you perceive as a possible challenge to this HIVST kit?

**E0038:** The challenge would be to interpret the results in order to be sure that the results are positive or negative. Are instructions included in this kit? – *usability of HIVST kits*

**PVK:** Yes instructions are included in the package.

**E0038**: Do I have to come back to the clinic to get results?

**PVK:** If you buy the kit, you don’t have to come to the clinic because you perform the test at home and you have to interpret your results by reading instructions. The instructions show when results are negative and when they are positive.

**E0038**: You said I can buy it is stores?

**PVK:** It is only in pharmacies for now.

**E0038**: Ok

**PVK:** What do you think would be the benefits of using this HIVST kit?

**E0038:** I don’t think that people are going to buy this kit if they are going to test themselves at home. They will be scared. Maybe they would not use it even if they buy it.

**PVK:** But if you buy it, it shows that you are willing to use it because it is not free, you spend your money to buy it.

**E0038**: Okay, I understand because some people don’t want to use clinics.

**Participant: 0039**

**SQT:** Have you tested for HIV?

**KM:** Yes

**SQT:** What made you test?

**KM:** Hmmm, It’s the kind of life we live you know it tends to happen sometimes that one doesn’t use a condom. So you have to know where you stand health wise.

**SQT**: What made you decide to test today?

**KM:** My problem is I feel my chest is not good, so I decided to check if everything is going ok overall with my health.

**SQT:** What is your testing routine?

**KM:** I can’t say I test every 3 months but I do test twice a year.

**SQT:** How big of an issue do you think HIV/AIDS is for people in your community and what makes it an issue of greater or lesser importance?

**KM:** People don’t care that much, it’s something that they are used to, and that you need to seek treatment. – Poor Health Seeking Behaviour

**SQT:** has the importance of HIV/AIDS changed in the community over the past few years and if so why?

**KM:** A lot, before if a person had HIV it was well known that the treatment was not that well developed, so you knew that if you had it there was no escaping it you will die. Now there’s treatment and you only take one pill and it’s more manageable that way.

**SQT:** Is it more of an issue for some groups than for others, and if so, which groups and why?

**KM:** It’s with the youth and here and there with adults but within the youth is where things are happening (sexually).

**SQT:** Is there denial or do they accept that AIDS is an issue?

**KM:** They accept that there’s an issue’

**SQT:** What is the reputation of the HIV testing locations in your community?

**KM:** I won’t lie because I don’t attend the clinic that much I think I went there twice and that was a long time ago.

**SQT:** Is testing there fully confidential

**KM:** Well I think so because I have not heard of anyone who tested there having their results shared with others.

**SQT:** How is the staff at the clinic?

**KM:** I won’t lie because I don’t really know them. I do not know how the workers are.

**SQT:** What do you think are the greatest barriers to HIV testing in your community?

**KM:** Its fear, like what if I test and the result is positive.

**SQT:** Are people’s attitudes and beliefs about HIV barriers?

**KM:** Yes and the fear of the unknown

**SQT:** What structural factors (e.g. availability of services, lack of resources, lack of privacy in the community, etc.)?

**KM:** No the clinic structure is fine

**SQT:** What do you know about HIVST?

**KM:** I didn’t know that much

**SQT:** Where did you hear about HIVST?

**KM:** From the people that pitch tents and do HIV testing around the community.

**SQT:** What do you perceive as possible challenges to HIVST?

**KM:** I don’t see challenges, except just the fear of doing the test alone

**SQT**: What do you think would be the benefits of HIVST

**KM:** It could help a person to know first then be able reason and counsel themselves should the result be positive

**Participant: 0040**

**SQT:** Have you tested for HIV?

**ZG:** yes

**SQT:** what made you test?

**ZG:** I had this thing that was like a pimple that use to grow right above my lip all the time so I decided to get tested. – Health Seeking Behavior

**SQT:** What made you decide to test today?

**ZG:** The last time I tested I was negative and at the time I used protection. But now I’m not sure because with the work I do I sometimes come into contact with blood. – HIV status awareness

**SQT:** How big of an issue do you think HIV/AIDS is for people in your community and what makes it an issue of greater or lesser importance?

**ZG:** It’s no longer a big issue the way I see it people talk about HIV and they disclose their status while in the past very few people spoke openly about HIV – *Reduced stigma and discrimination*

**SQT:** has the importance of HIV/AIDS changed in the community over the past few years and if so why?

**ZG:** I think it has, I mean people are more educated which has empowered them, they know more and most of them don’t see HIV as a death sentence anymore.

**SQT:** Is it more of an issue for some groups than for others, and if so, which groups and why? I would say it’s an issue for all, old and young it affects everyone

**ZG:** I see it as an issue that affects us all, young and old.

**SQT:** Is there denial or do they accept that AIDS is an issue?

**ZG:** Theirs no longer denial there’s acceptance.

**SQT:** What is the reputation of the HIV testing locations in your community?

**ZG:** Though I’ve only been there twice but I can say that their service is really bad, people wait for a long time, to point where people don’t use that clinic they now go to Makhuta clinic.

**SQT:** Do you know of any centre/clinic where you can test within your community?

**ZG:** Ja it is confidential.

**SQT:** What do you think are the greatest barriers to HIV testing in your community?

**ZG:** Ay they do not care, they sit around they talk to you however they like that clinic is bad.

**SQT:** Are people’s attitudes and beliefs about HIV barriers?

**ZG:** Most people in my area are lazy and when they get there and see so many of their neighbours there at the clinic, which is why they end up going to Makhuta clinic. At times they (nurses) would unintentionally ask a patient with others around when last they did their cd4 count.

**SQT:** What structural factors (e.g. availability of services, lack of resources, lack of privacy in the community, etc.)?

**ZG:** The structure is fine it’s the workers that are the issue to the point that there were rumours that Adams Clinic will close down.

**SQT:** What do you know about HIVST?

**ZG:** I do not know much.

**SQT**: Where did you hear about HIVST?

**ZG:** What I do know I’ve heard from work meetings and also on the radio.

**SQT:** What do you perceive as possible challenges to HIVST?

**ZG:** I do not know how and by whom the individual that will be buying the kit would have been educated on how to use the kit. When they buy it will they be educated prior to the purchase? - Education

**SQT:** What do you think would be the benefits of HIVST?

**ZG:** It will help that the clinics will not be full, the clinic work load will decrease and the community will benefit greatly.
